# Inclusion Complexations of Levamisole and Cocaine With Molecular Baskets. Fluorescence Displacement Assay for Detection of Cocaine and Its Adulterant Levamisole in Aqueous Media

**DOI:** 10.1002/chem.71216

**Published:** 2026-06-03

**Authors:** Christopher T. Mortensen, Carson E. Ward, Nitesh Kumar, Logan Willemann, Ryan Lutz, Kailey Hughes, Zheng Zheng, Jovica D. Badjić

**Affiliations:** ^1^ Department of Chemistry & Biochemistry The Ohio State University Columbus Ohio USA

**Keywords:** chemosensors, cocaine, fluorescence displacement assay, inclusion complexation, levamisole

## Abstract

We investigated inclusion complexation of levamisole (LEV^+^), a common adulterant of cocaine (COC^+^), with six molecular baskets **1** ^3−^ **–6** ^18−^ of increasing cavity depth and anionic charge at the portal. ^1^H NMR and mass spectrometry showed that **1** ^3−^ to **6** ^18−^ bind to LEV^+^ to form binary complexes in water, with the stability increasing across the series and highest for dendritic basket **6** ^18−^ (*K*_d_ = 10 µM). From ^1^H NMR magnetic shielding and OPLS4 calculations, we deduced that LEV^+^ inserts its benzene in the binding pocket of **1** ^3−^–**6** ^18−^ while the protonated heterocycle remains near the anionic portal. The stability of [LEV⊂**6**]^17−^ arises from C−H^…^π/cation^…^π/π−π contacts, ion pairing, and hydrophobic effect (Δ*H°*< 0, ITC). Dendritic basket **6** ^18−^ was subsequently used to develop a fluorescent displacement assay (FDA) for the detection of LEV^+^ with alizarin red S (ALZ^−^) as a fluorescence reporter. LEV^+^ displaced the dye to generate a fluorescence response suitable for its detection in water over circa 10–300 µM range. In contrast, COC^+^ bound more strongly to basket **6** ^18−^, forming binary (*K*_d_ = 1 µM) and ternary complexes (^1^H NMR, calorimetry, and FDA). The binding mode of COC^+^ differs from that of LEV^+^, with the drug's cationic fragment occupying the basket's cavity.

## Graphical abstract

Levamisole is a common adulterant found in street‐distributed cocaine. Molecular baskets have been shown to form stable inclusion complexes with both substances. While levamisole inserts its benzene into the basket's cavity, cocaine utilizes its bicyclic ring for encapsulation. Both drugs displace a fluorescent dye, initially occupying the basket's cavity, to produce a fluorescence signal enabling their identification.

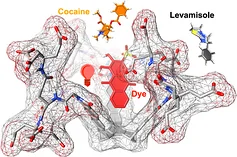

## 1 Introduction

Levamisole (Figure 1A) is a veterinary antiparasitic medicine that removes nematodes by binding to their nicotinic acetylcholine receptors in muscles, thereby causing spastic paralysis (i.e., involuntary muscle stiffness) [1]. In the nineties, levamisole was used as a clinical agent, Ergamisole, along with fluorouracil, to treat colorectal cancer in humans [2]. Due to adverse side effects [3], the Food and Drug Administration (US) pulled the drug from the market in 2000. Interestingly, one of levamisole's metabolites is aminorex [4]. The molecule is a strong psychostimulant that, akin to amphetamine, blocks the uptake of dopamine transporters for, allegedly, enhancing the stimulative effects of cocaine (Figure 1A) [5]. This could be a reason that levamisole has, for more than two decades, been used as the main adulterant in illicit samples of cocaine, apart from the cost and availability [6]. Importantly, the consumption of levamisole‐tainted cocaine causes serious and toxic side effects (e.g., agranulocytosis, leukopenia, purpura, and visible necrotized skin tissue) [7]. A need to detect and quantify the drug in street‐distributed samples or biological liquids [8] prompted the development of an array of electrochemical, chromatographic, and mass spectrometric methods [8, 9, 10, 11]. With regard to our study, DNA aptamers were recently shown [12] to selectively bind to cocaine in the presence of levamisole, whereas a mixture of hosts could be used for chemosensing and differentiation of common illicit drugs and adulterants by visible spectroscopy and principal component analysis [13]. In line with these findings, we wondered: Do molecular baskets (Figure 1B,C), developed in our laboratory [14, 15, 16] and holding negative amino acids at the portal, form inclusion complexes with levamisole and cocaine? If so, do the stability of such [levamisole⊂basket] or [cocaine⊂basket] complexes (Figure 1B,C) differ? At last, can we develop a rapid and facile method for the detection of these drugs, with their presence triggering a detectable change in color/luminescence of the solution via an indicator displacement assay [17, 18] (IDA, Figure 1D)? While the inclusion complexation of cocaine has been studied with abiotic hosts [19, 20, 21], there are only a few reports [22, 23, 24] that describe the binding of levamisole.

**FIGURE 1:**
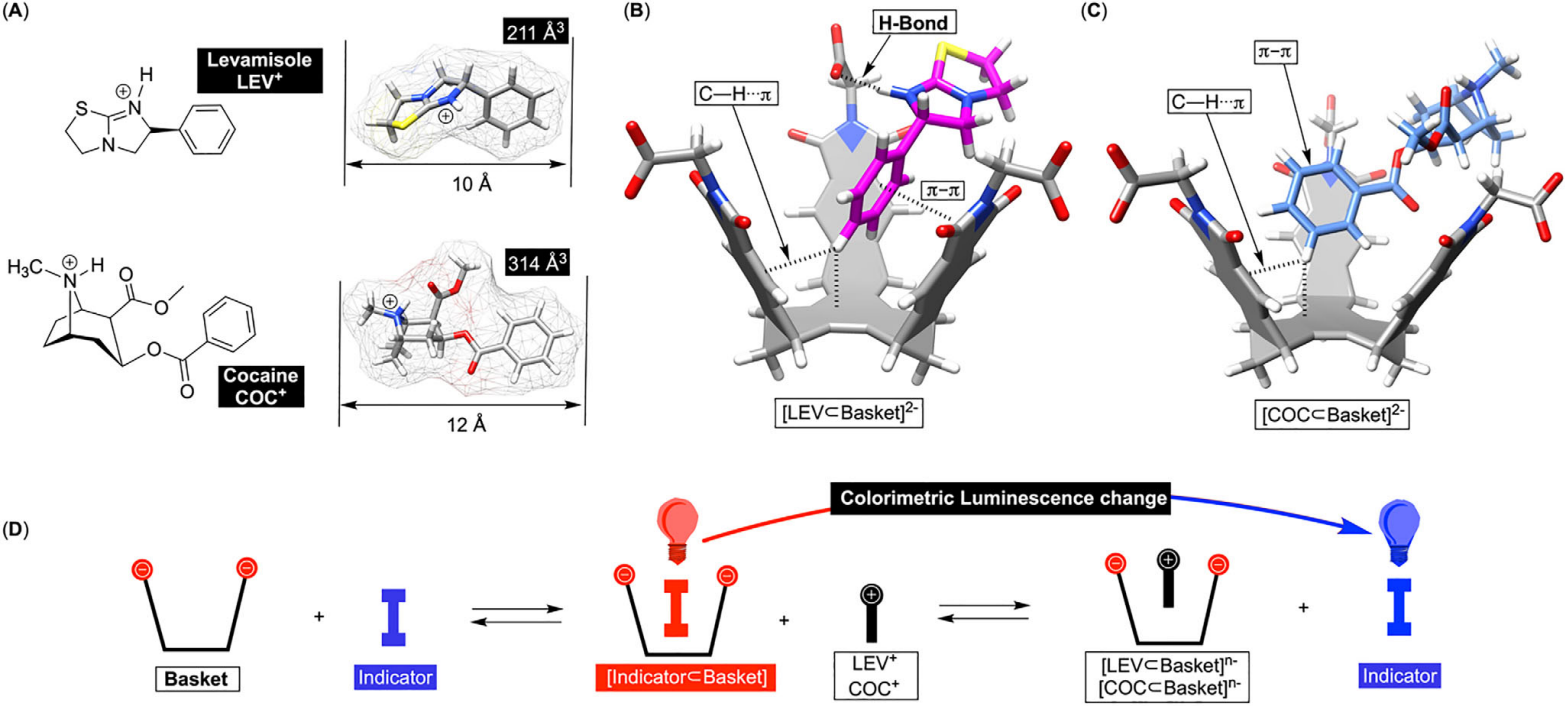
(A) Chemical and energy minimized (DFT:B3LYP) structures of levamisole (LEV^+^) and cocaine (COC^+^). (B,C) Energy minimized (OPLS 4) structures of [LEV⊂basket]^2−^ and [COC⊂basket]^2−^ complexes showing complementarity and intermolecular noncovalent contacts. (D) A schematic representation of the indicator displacement assay (IDA) for the detection of LEV^+^, COC^+^, and/or their mixture.

In aqueous media at pH = 7.0, levamisole (LEV^+^, p*K*_a_ ∼ 7) and cocaine (COC^+^, p*K*_a_ = 8.6) are herein taken as monocationic compounds. They are relatively small, comparable in volume/length (Figure 1A), and hold a positive charge at the top of the benzene ring. On the basis of earlier studies [25, 26] and preliminary computational results in Figure 1B,C, both drugs were expected to dock their nonpolar benzene inside the basket's inner space. If so, the positively charged site is, for each drug, in the vicinity of negatively charged amino acids at the basket's rim [15]. With the apparent host‐guest complementarity, we went on to study inclusion complexations of LEV^+^ and COC^+^ using a series of molecular baskets having differently sized cavities and amino acid groups at the rim (Figure 2). The goal was to learn about the complexation of the two drugs in water and potentially develop colorimetric/luminescent assays [27] for their detection.

**FIGURE 2:**
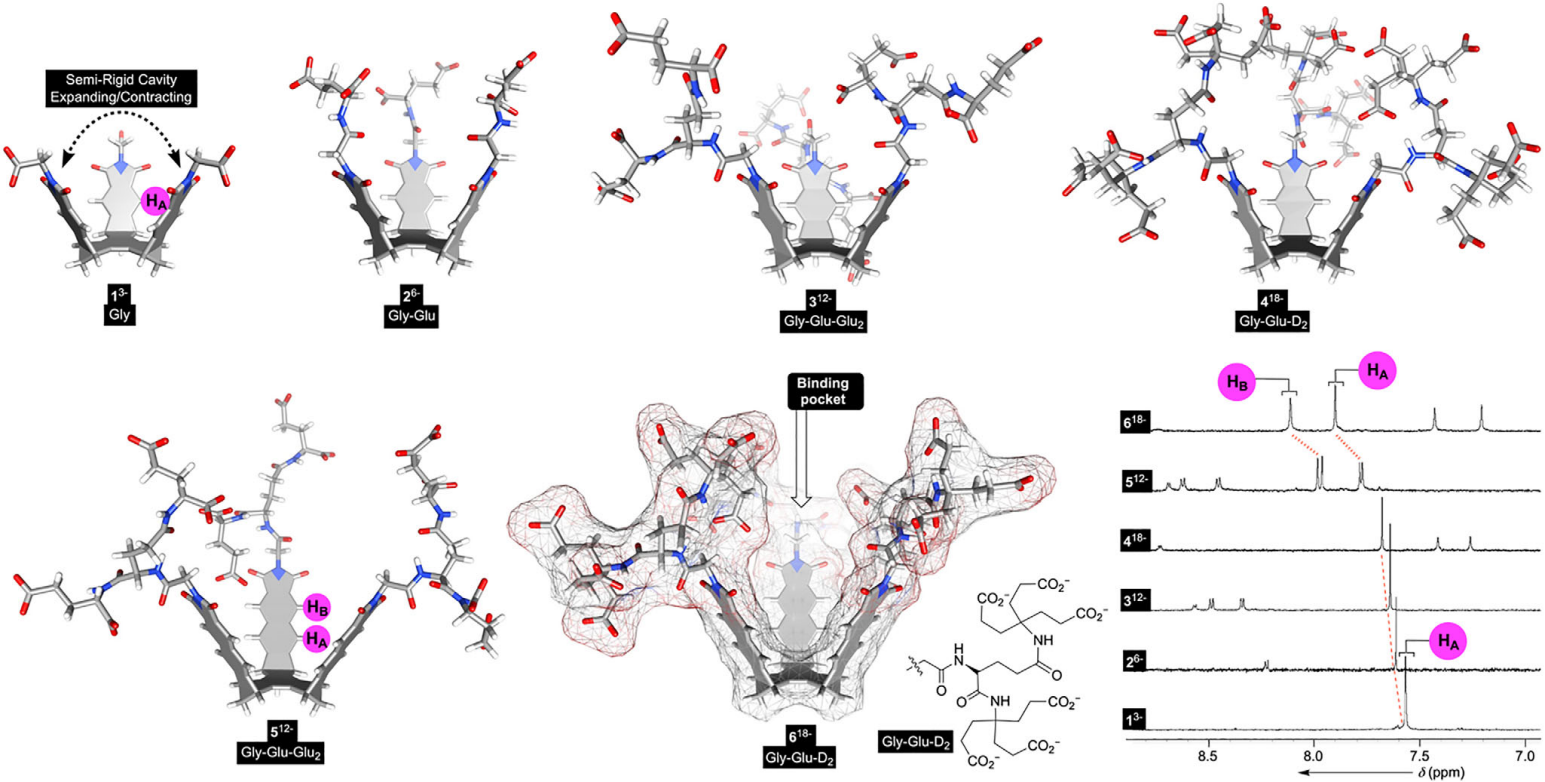
Molecular baskets **1** ^3−^ to **4** ^18−^ with phthalimide and **5** ^12−^/**6** ^18−^ with naphthalimide cavity carry four increasingly bigger and more anionic groups at the portal (OPLS4); for chemical structures of baskets **1** ^3−^ to **6** ^18−^, see Schemes S1–S4. The nonpolar binding pocket from **6** ^18−^ is shown (Chimera). A segment of ^1^H NMR spectra (600 MHz, 298 K) of baskets **1** ^3−^–**6** ^18−^ in 30 mM phosphate buffer at pH = 7.0 (water suppression, 10% D_2_O); note that phthalimide‐based baskets **1** ^3−^ to **4** ^18−^ possess one type of aromatic H_A_ protons while naphtalimide‐based **5** ^12−^–**6** ^18−^ have two types of aromatic H_A_/H_B_ protons.

## 2 Results and Discussion

### 2.1 Screening Molecular Baskets for Complexing Levamisole (LEV^+^)

Molecular baskets are cup‐shaped cavitands with a semi‐rigid cavity (Figure 2) [28]. Their side walls include phthalimide, naphthalimide, and other aromatics or their combination to give hosts with (a) variously sized, (b) shaped, or (c) inherently chiral inner space [28, 29, 30]. A set of charged groups (e.g., amino acids) is installed at the rim of baskets for (a) enabling solubility in aqueous media, (b) engaging in noncovalent interactions with guests, and/or (c) preventing self‐association [16]. For probing the inclusion of levamisole (LEV^+^, Figure 1B), we decided to screen six baskets **1** ^3−^−**6** ^18−^ (Figure 2) of which **1** ^3−^−**4** ^18−^ possess shorter (i.e., phthalimide‐based) while **5** ^12−^−**6** ^18−^ possess longer (i.e., naphthalimide‐based) cavities. As for the portal of these six cavitands, amino acids/peptides with increasingly greater size, branching, and negative charge (−3 to −18, in water at pH = 7.0) are included. As such, basket **1** ^3−^ is shallow, holding three small glycines at its top with three negative charges in total. In contrast, basket **6** ^18−^ is deep, holding 3 large dendrons (Gly‐Glu‐D_2_, D stands for branched Behera's amine) [23] and 18 negative charges in total. A well‐defined, semi‐rigid [31], and nonpolar binding pocket of basket **6** ^18−^ is shown in Figure 2. ^1^H NMR spectra of **2** ^6−^−**6** ^18−^ at their lower concentrations (30 mM phosphate buffer at pH = 7.0) revealed a negligible change in the chemical shift of signals with concentration (Figures S9–S12). In line with our earlier studies of **1** ^3−^ and **5** ^12−^ [16, 32], the result is in line with all six baskets populating their monomeric state at lower concentrations in water. Interestingly, ^1^H NMR signals from phthalimide H_A_ protons of **1** ^3−^−**4** ^18−^ at circa 7.6 ppm (Figure 2) underwent a steady and downfield shift along the basket series. With more anionic groups at the basket's portal, protons H_A_ are clearly less magnetically shielded. Since negative charges repel, we deduce [30] that three phthalimides with more anionic groups from **1** ^3− ^to **4** ^18−^ push each other away more effectively along the series to result in reduced diamagnetic shielding that the aromatics experience. The trend was also observed for baskets **5** ^12−^ and **6** ^18−^ (Figure 2), in which the latter cavitand develops a wider cavity. To sum up, baskets **1** ^3−^−**6** ^18−^ vary in (a) cavity size, (b) anionic charge at the portal, and (c) the width of their adaptable cavity. Will **1** ^3−^−**6** ^18−^ bind LEV^+^ drug and, if so, show any trend in the stability of [LEV⊂**1**−**6**]^n−^ complexes? Will the mode of binding alter along the series?

An incremental addition of a standard solution of LEV^+^ to each basket, dissolved in 30 mM phosphate buffer at pH = 7.0, was monitored with ^1^H NMR spectroscopy (Figures 3A and S13–S18). While signals from **1** ^3−^−**6** ^18−^ were only slightly perturbed during titrations, H_1_–H_4_ resonances from LEV^+^ (Figure 3A) became magnetically shielded, suggesting this molecule occupies the aromatic interior of baskets [33]. All binding isotherms fit well to 1:1 complexation model (Figures 3A and S19–S24) [34], with estimated binding affinities (*K*_a_) spanning two orders of magnitude and ranging from 2.0 · 10^3^ M^−1^ to 1.3 · 10^5^ M^−1^ (Figure 3A); ESI mass spectrometry data (Figures S25–S30) corroborated these findings by showing ions with the mass corresponding to binary [LEV⊂**1**−**6**]^+^ species. Evidently, the inclusion affinity for LEV^+^ occupying **1** ^3−^ to **6** ^18−^ increases along the series, with dendritic basket **6** ^18−^ acting as the most effective host. It follows that increasing the overall negative charge at the basket's portal and the cavity depth has a positive effect on the stability of binary [LEV⊂basket]^n−^ complexes. The observation indicates the role of (a) permanent ion‐ion interactions and (b) the hydrophobic effect in the inclusion complexation [35]. Indeed, the results from calorimetric titration of LEV^+^ to **6** ^18−^ revealed the exothermic formation of [LEV⊂**6**]^17−^ (Δ*H°* = − 9.7 kcal/mol, Figure S31), therefore, in line with LEV^+^ displacing high‐energy water molecules from the basket's nonpolar binding pocket [36]. Next, we went on to examine the mode(s) of binding. When the degree of magnetic shielding (Δ*δ* = *δ*_bound_—*δ*_free_) of ^1^H NMR resonances H_1_–H_4_ from LEV^+^ was plotted for **1** ^3−^−**6** ^18−^ (Figure 3B), it became apparent that H_1_ is the most shielded proton across the series. In fact, the diamagnetic effect gradually drops from H_2_ to H_4_ and thus commensurate with the benzene from levamisole occupying the basket's aromatic cavity and positioning its H_1_ close to the base where the diamagnetic shielding is the greatest (Figure 3B) [33]. This docking pose places positively charged imidazothiazole from LEV^+^ in the vicinity of negatively charged carboxylates at the rim of the baskets. In this regard, Monte‐Carlo conformational searches of [LEV⊂**1**‐**6**]^n−^ inclusion complexes in implicit water solvent (OPLS4, Figures S50–S55) revealed a comparable set of binding poses that in each case support ^1^H NMR spectroscopic observations. As a representative, complex [LEV⊂**6**]^17−^ in Figure 3C shows benzene from LEV^+^ occupying the cavity of **6** ^18−^ and (a) placing its H_1_ against the cavitand's base to engage in C─H···π intermolecular contacts while simultaneously (b) facing the cavitand's naphthalimide side to form π−π contacts. Moreover, positively charged imidazothiazole is forming cation−π contacts with the naphthalimide while the carboxylate dendrons are spectators residing in bulk solvent. It is important to note that computational results for [LEV⊂**1**‐**6**]^n−^ also include a population of conformers (within c.a. 1 kcal/mol of the steric energy window) in which carboxylates at the portal are forming charged hydrogen bonding contacts with the protonated nitrogen from LEV^+^.

**FIGURE 3:**
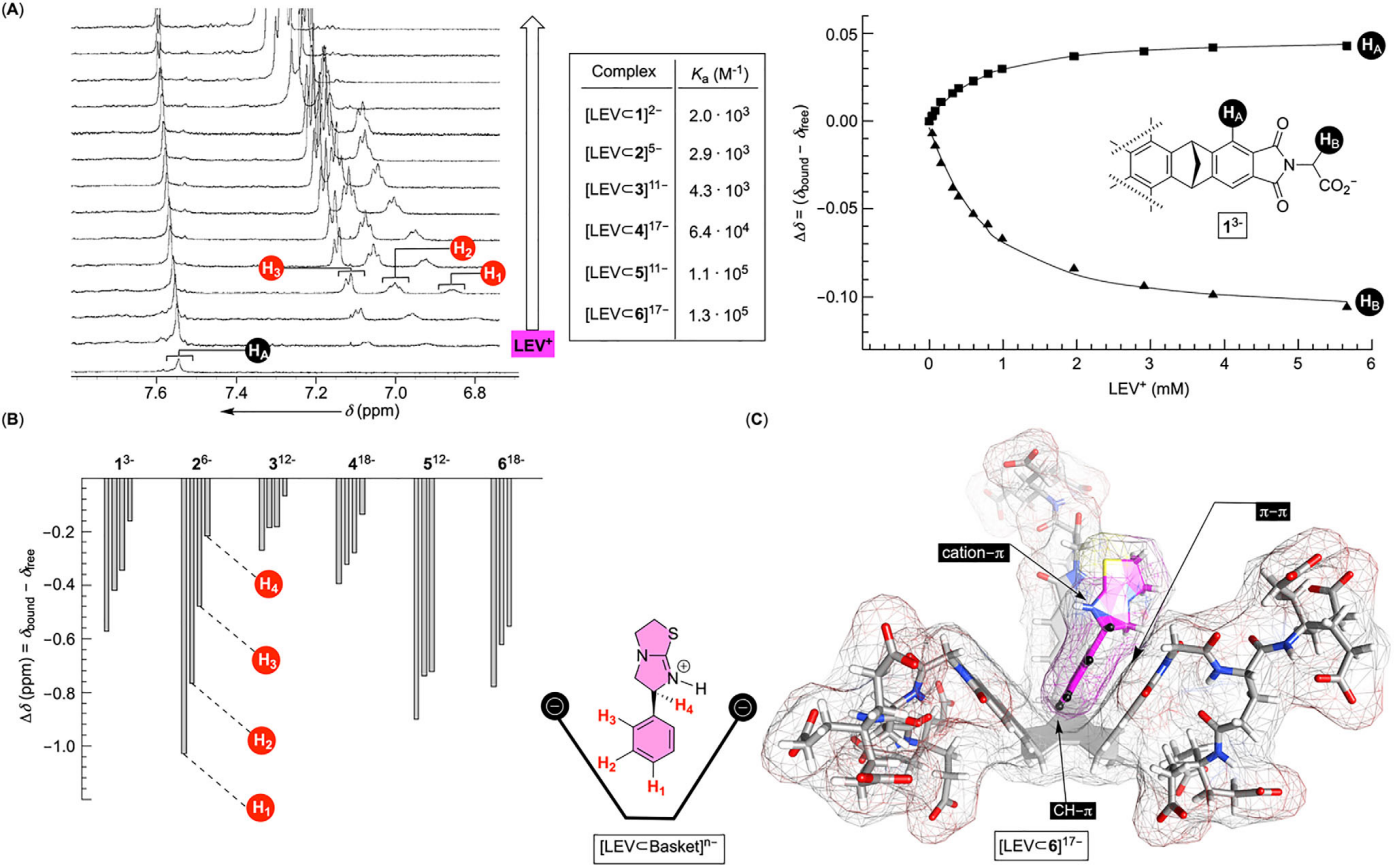
(A) (left) A segment from ^1^H NMR spectra (600 MHz, 298 K) of 0.41 mM basket **1** ^3−^ (30 mM phosphate buffer at pH = 7.0) obtained after incremental addition of LEV^+^. (Right) A steady ^1^H NMR chemical shift of H_A_ and H_B_ signals from **1** ^3−^ as a function of increasing concentration of LEV^+^ fit well (Musketeer) to the formation of binary [LEV⊂**1**]2^−^ complex with *K*_a_ = 2.0 · 10^3^ M^−1^. (Middle) Association constants (*K*_a_) corresponding to the formation of binary [LEV⊂**1**‐**6**]^n−^ complexes were obtained from ^1^H NMR supramolecular titrations (Figures S19–S24). (B) A change in ^1^H NMR chemical shifts of H_1_─H_4_ protons from LEV^+^ upon its complexation with baskets **1** ^3−^ to **6** ^18−^; (right) the proposed mode of inclusion of LEV^+^. (C) Energy‐minimized structure of [LEV⊂**6**]17^−^ from Monte‐Carlo conformational search (OPLS4, Figure S55).

With dendritic basket **6** ^18− ^showing the highest affinity toward LEV^+^ in water, we decided to use this host for (a) developing an indicator displacement assay (IDA) of the drug and (b) examining the inclusion complexation of cocaine (COC^+^).

### 2.2 Selecting Dye for UV–Vis Indicator Displacement Assay (IDA) and Detection of Levamisole

The binding affinity of LEV^+^ for dendritic basket **6** ^18−^ approaches the limit for reliable quantification by ^1^H NMR spectroscopy [37]. With the aim of developing an IDA assay for colorimetric detection [38] of levamisole (Figure 4A), we began by measuring the thermodynamic stability of [LEV⊂**6**]^17−^ with UV–vis spectroscopy. While both LEV^+^ and basket **6** ^18−^ absorb light at lower wavelengths (< 400 nm, Figure S32), the titration data revealed a negligible perturbation of their UV–vis spectra upon the complex formation (Figure S33). Following, we went on to identify a dye molecule that forms an inclusion complex with **6** ^18−^ (i.e., [dye⊂**6**]^n−^) [39]. A displacement of the bound dye from such [dye⊂**6**]^n−^ complex with LEV^+^ is to release the dye for creating a measurable spectroscopic change (Figure 4A). The change can be subjected to nonlinear regression analysis for (a) quantifying the stability [39] of [LEV⊂**6**]^17−^ and (b) creating an IDA calibration curve for determining unknown concentrations of LEV^+^ [27]. After UV–vis/NMR screening of the complexation of common organic dyes [17] with **6** ^18−^, we found that alizarin red S (ALZ^−^, Figure 4A) gave rise to a binary complex [ALZ⊂**6**]^19−^. The global fitting [34] of UV–vis titration data for the formation of [ALZ⊂**6**]^19−^ from **6** ^18−^ and ALZ^−^ (Figure 4B) was satisfactory with *K*_a_ = 5.6 · 10^4^ M^−1^ (see also, Figures S34 and S35). With the anionic dye having comparable affinity for occupying the cavity of dendritic basket **6** ^17−^ as positively charged LEV^+^ [40], we proceeded with developing the IDA. Regrettably, the degree of the observed change in absorbance from the bound versus free ALZ^−^ was in the displacement insufficient (Figure S36) to quantify the competing equilibria. Accordingly, we moved to investigate the binding and displacement of ALZ^−^ using fluorescence spectroscopy.

**FIGURE 4:**
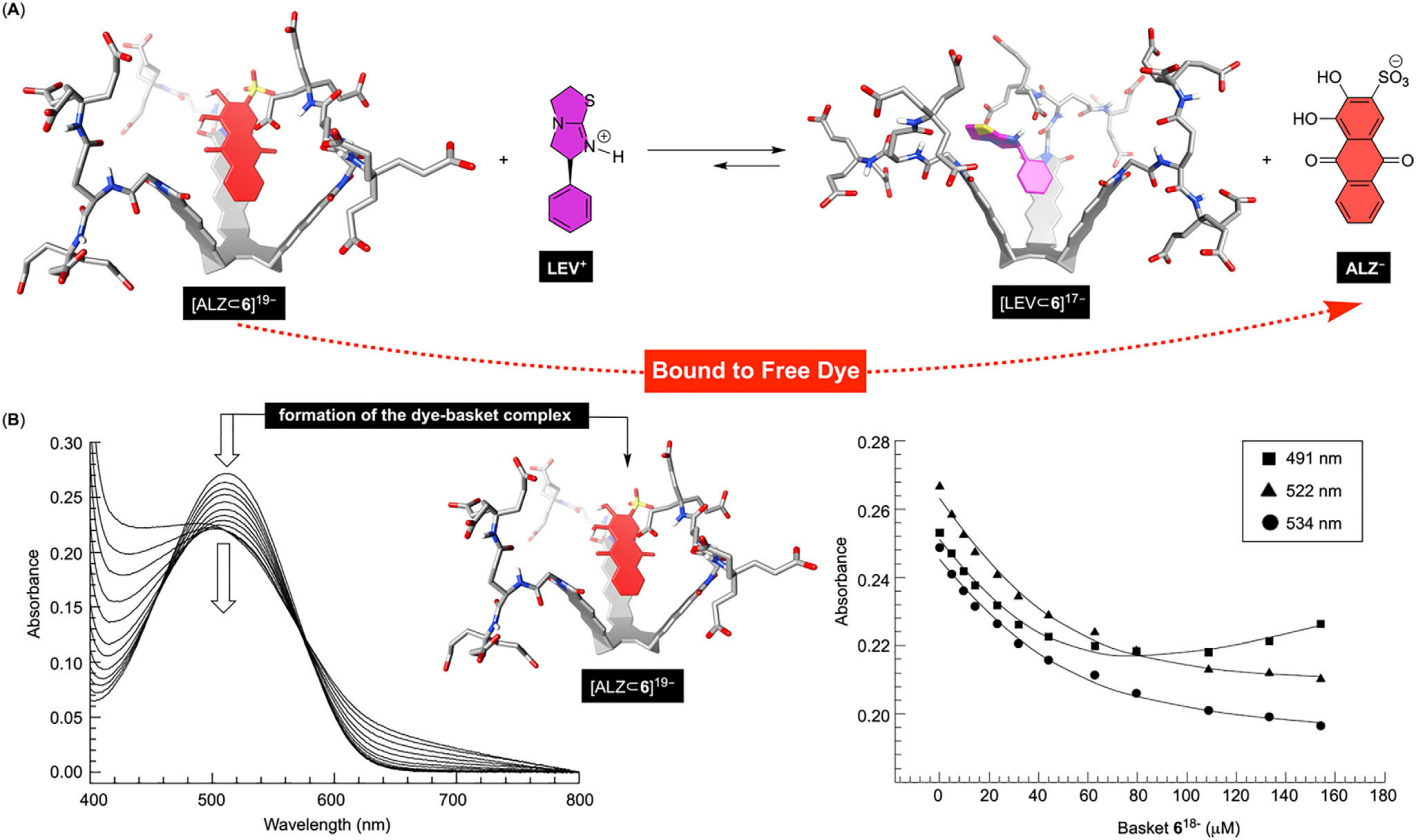
(A) Energy minimized (OPLS4) and chemical structures of [ALZ⊂**6**]19^−^ and levamisole (LEV^+^) in equilibrium with [LEV⊂**6**]17^−^ and alizarin red S (ALZ^−^). (B) UV–vis spectra of 50 µM ALZ^−^ in 30 mM phosphate buffer at pH = 7.0, before and after an incremental addition of 0.41 mM standard solution of dendritic basket **6** ^18−^. (Right) The global fitting (400–799 nm, Musketeer) of titration data (left) to a 1:1 model of binding was satisfactory with *K*_a_ = 5.6 · 10^4^ M^−1^.

### 2.3 Fluorescence Displacement Assay (FDA) for Detection of Levamisole (LEV^+^)

Alizarin red S showed negligible (zero) fluorescence in the phosphate buffer [41] used in our study. Consequently, we completed binding studies in 100 mM borate buffer at pH = 8.1 (p*K*_a_ of boric acid is 9.14–9.24), wherein the alizarin showed sufficient emission upon excitation to (with/without the basket host) permit the measurements (Figure 5A). In association with the new buffer system, we first quantified the inclusion of LEV^+^ within dendritic basket **6** ^18−^ using ^1^H NMR spectroscopy (Figures S37 and S38). The binding isotherm fits well to the formation of binary [LEV⊂**6**]^17−^ having the stability of *K*_a_ = 1.3 · 10^5^ M^−1^; while at pH = 8.1, levamisole is predominantly in its neutral form in bulk solution but also in equilibrium with LEV^+^, we hypothesize that LEV^+^ is likely occupying the inner space of **6** ^18−^ wherein it becomes stabilized (i.e., having different p*K*_a_). The observed magnetic perturbations of proton nuclei from LEV^+^ during the complexation (Figure S39) are in line with the earlier proposed mode of inclusion (Figure 3B/C). As for the stability of [ALZ⊂**6**]^19−^ in the borate buffer, we monitored a change in fluorescence of the dye upon incremental addition of a standard solution of **6** ^18−^ (Figure 5A). While the inclusion of alizarin led to the enhancement of fluorescence, the binding isotherm fit well [34] to the formation of [ALZ⊂**6**]^19−^ with *K*_a_ = 3.1 · 10^3^ M^−1^ (see also Figures S40 and S41). With the known thermodynamic stabilities of [LEV⊂**6**]^17−^ and [ALZ⊂**6**]^19−^ [39], we were set to establish a fluorescence displacement assay (FDA, Figure 5C). An incremental addition of a standard solution of LEV^+^ to a solution of ALZ^−^ and dendritic basket **6** ^18−^, comprising [ALZ⊂**6**]^19−^ complex, led to a decrease of fluorescence intensity from the alizarin dye (Figure 5B). The spectroscopic change was expected, corresponding to a displacement of the bound ALZ^−^ within [ALZ⊂**6**]^19−^ with LEV^+^ to give [LEV⊂**6**]^17−^ and free ALZ^−^ (Figure 5C). After the spectroscopic data were subjected to global fitting (480–640 nm, Figure 5B) [34], the stability of [LEV⊂**6**]^17−^ was found to be *K*_a_ = 1.7 · 10^5^ M^−1^. The value of the binding constant is close to the one obtained from ^1^H NMR spectroscopic titration (*K*_a_ = 1.3 · 10^5^ M^−1^), therefore providing validity to these measurements. Binding isotherms from FDA present calibration curves for determining the unknown concentration of LEV^+^ in aqueous samples of interest (circa 10–300 µM). Indeed, after a standard solution of LEV^+^ was added to the FDA solution (Figure 5B) so that its calculated concentration is 84 µM, the measured drop in fluorescence suggested 78 µM concentrated LEV^+^ (Figure S42).

**FIGURE 5:**
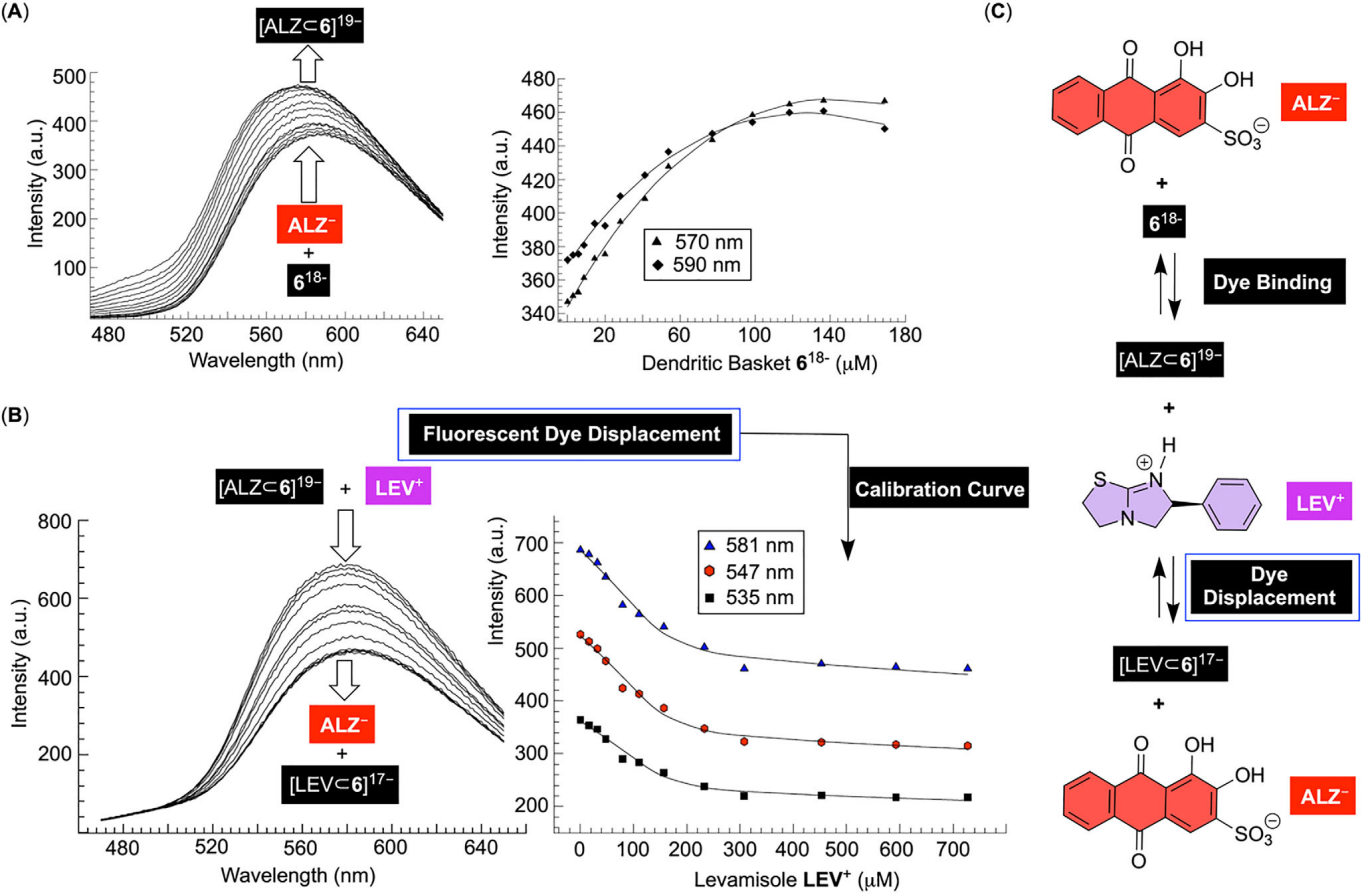
(A) Fluorescence spectra of 50.1 µM ALZ^−^ in 0.1 M borate buffer at pH = 8.1, before and after an incremental addition of a standard solution of dendritic basket **6** ^18−^. (Right) The global fitting (550–590 nm, Musketeer) of the titration data to a 1:1 model of binding was satisfactory with *K*_a_ = 3100 M^−1^. (B) Fluorescence spectra of 80 µM ALZ^−^ and 160 µM basket **6** ^18−^ in 100 mM borate buffer at pH = 8.1 before and after an incremental addition of a standard solution of LEV^+^. (Right) The global fitting (471–650 nm, Musketeer) of the titration data to a displacement model was satisfactory with the stability *K*_a_ = 171000 M^−1^ of [LEV⊂**6**]18^−^; the stability of [ALZ⊂**6**]19^−^, *K*_a_ = 3100 M^−1^, was included as a nonfit parameter. (C) A schematic representation of the fluorescent displacement assay (FDA) for the detection of LEV^+^ in aqueous media.

### 2.4 Inclusion Complexation, Fluorescence Displacement Assay (FDA), and Detection of Cocaine (COC^+^)

Does dendritic basket **6** ^18−^ form an inclusion complex with cocaine (COC^+^)? If so, how selective is the binding of COC^+^ versus LEV^+^? We used ^1^H NMR spectroscopy to monitor incremental addition of a standard solution of COC^+^ to basket **6** ^18−^ in borate buffer (Figure 6A); note that COC^+^ is in the monomeric state within the examined range of concentrations (Figure S43) and is chemically stable to hydrolysis for at least 6 h (Figure S44). A steady shift and broadening of ^1^H NMR resonances from the basket indicated noncovalent complexation(s) taking place, fast on the chemical shift time scale. Importantly, aromatic H_A_/H_B_ signals from the basket's naphthalimides underwent magnetic perturbations with each undergoing a downfield followed by an upfield shift (Figure 6A). The observation suggested [42] two consecutive binding events in which, we posit, the first corresponds to the formation of a binary [COC⊂**6**]^17−^ while the second is a ternary [COC_2_⊂**6**]^16−^ complex; molecular baskets have already been found [15] to associate with nonpolar drugs using both the bowl‐shaped cavity and its nonpolar outer surface. Since two aromatic signals from host **6** ^19−^ were magnetically perturbed more than others (Figure S46), we reasoned that one of the binding events includes COC^+^ occupying the aromatic cavity of the host. The results of calorimetric titration (Figure 6B) were in line with the formation of binary [COC⊂**6**]^17−^ (*K*_1_ = 2.5 · 10^6^ M^−1^) and ternary [COC_2_⊂**6**]^16−^ (*K*_2_ = 5.2 · 10^3^ M^−1^) complexes. The first binding event is driven by enthalpy (Δ*H°* = −7.4 kcal/mol), which is the trademark of inclusion complexations and high‐energy water molecules displacement from concave/nonpolar binding sites (i.e., non‐classical hydrophobic effect) [36]. With the stability of [COC⊂**6**]^17−^ in the micromolar range, we were curious about the mode of inclusion. While ^1^H NMR signals from free/bound COC^+^ were severely broadened during the titration experiment, one of the proton nuclei from the drug's benzene ring appeared downfield (Figure S48) while the ^+^N−Me group shifted upfield (Figure S49). Consequently, the bicyclic portion of COC^+^ occupies the cavity of **6** ^18−^ while the benzene resides around the portal. Indeed, Monte‐Carlo conformational search of [COC⊂**6**]^17−^ (Figures 6C and S56) revealed the drug docking its bicyclic ring in the basket's binding pocket to form numerous C─H···π and cation−π contacts. In contrast, the benzene ring is at the portal, leaning against one of the naphthalimides to engage in π−π noncovalent bonding. Despite the large affinity gap of **6** ^18−^ toward COC^+^ and ALZ^− ^[39], we went on to probe the displacement of alizarin from [ALZ⊂**6**]^19−^ with COC^+^ (Figure 6D). An incremental addition of the drug to a solution of **6** ^18−^ and ALZ^−^ (i.e., [ALZ⊂**6**]^19−^) resulted in a steady decrease of the observed fluorescence, manifesting the formation of free dye alongside [COC⊂**6**]^17−^. While ignoring the formation of ternary [COC_2_⊂**6**]^16−^, the data were fit to a model comprising two competing, equilibria (shown in Figure 5C) yielding *K*_a_ = 4.2 · 10^6^ M^−1^ for the formation of [COC⊂**6**]^17−^. In this way, the estimated stability of [COC⊂**6**]^17−^ from FDA measurement is close to the one obtained from calorimetry. To sum up, dendritic basket **6** ^18−^ has an order of magnitude greater affinity for binding cocaine (COC^+^) than levamisole (LEV^+^), likely due to distinct modes of molecular inclusion.

**FIGURE 6:**
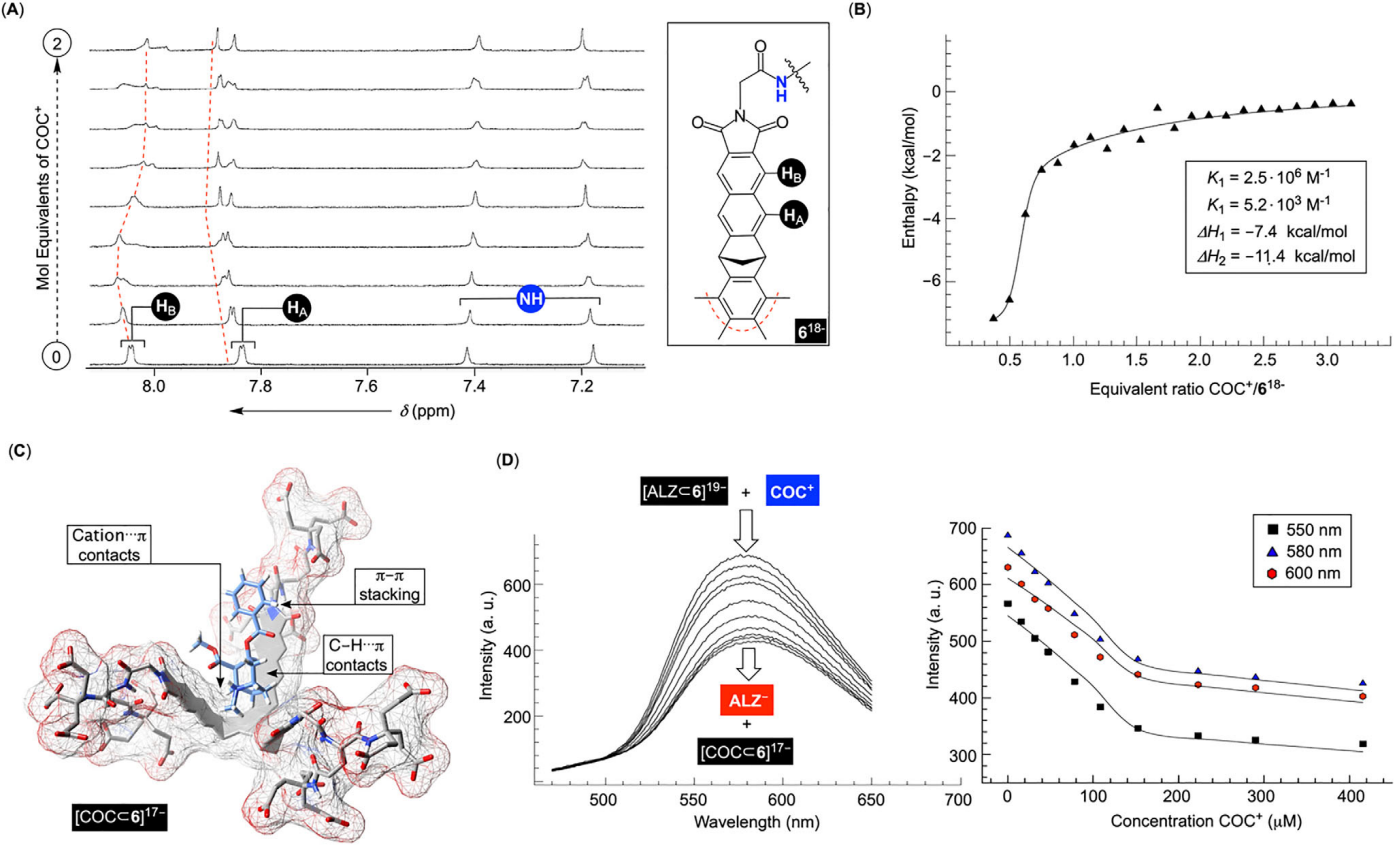
(A) A segment of ^1^H NMR spectra (850 MHz, 298 K) of 50 µM dendritic basket **6** ^18−^ (100 mM borate buffer at pH = 8.1) before and after incremental addition of 250 mM COC^+^. (B) An incremental addition of a standard solution of COC^+^ to a 200 µM solution of dendritic basket **6** ^18−^ (100 mM borate buffer at pH = 8.1) was monitored with isothermal titration calorimetry (ITC) at 298.0 K and the data fit to a 1:2 model of complexation. (C) The most stable docking pose of energy minimized [COC⊂**6**]17^−^ (OPLS4, Monte Carlo; Figure S56). (D) Fluorescence spectra of 80 µM ALZ^−^ and 160 µM basket **6** ^18−^ in 0.1M borate buffer at pH = 8.1 before and after an incremental addition of a standard solution of COC^+^. (Right) The global fitting (471‐650 nm, Musketeer) of the titration data to a displacement model gave *K*_a_ = 4.2 · 10^6^ M^−1^ for [COC⊂**6**]17^−^.

## 3 Conclusion

In conclusion, we employed ^1^H NMR spectroscopy to examine the binding affinity of levamisole toward six molecular baskets. The thermodynamic stability of resulting [levamisole⊂basket]^n−^ complexes increased with both cavity depth and the anionic charge of the groups positioned at the basket's portal. In particular, dendritic basket **6** ^18−^ exhibited 10 µM affinity for levamisole and, to the best of our knowledge, represents the strongest abiotic host reported for this drug to date. We further used alizarin red S as a fluorescence indicator to develop a fluorescence displacement assay (FDA) for the detection (circa 10–300 µM range) of levamisole in water. Importantly, dendritic basket **6** ^18−^ bound cocaine with circa 1 µM affinity, which is comparable to that of the strongest known abiotic hosts for this drug, including pillar[6]maxQ [20] and calabadion [21]. Using the same fluorescence indicator, we also developed an FDA for the detection of cocaine in aqueous media. While levamisole occupies the cavity of dendritic basket **6** ^18−^ with its benzene ring engaging in attractive π−π and C─H···π contacts, cocaine docks its positively charged bicyclic unit, thereby forming cation−π, π−π, and C─H···π noncovalent bonds. These differences in the mode of inclusion, together with the hydrophobic effect and ion pairing, are likely responsible for the observed order‐of‐magnitude difference in the binding selectivity. Given this selectivity pattern, our FDAs are only suitable for the individual detection of levamisole (10–300 µM range) and cocaine (10–120 µM range). Nevertheless, the study sets a foundation for using baskets as modular cavitands to investigate complexation of these and similar drugs in aqueous environments.

## Conflicts of Interest

The authors have no conflicts of interest.

## Supporting information




**Supporting File**: chem71216‐sup‐0001‐SuppMat.pdf

## Data Availability

The data that support the findings of this study are available from the corresponding author upon reasonable request.
