# Supplementary material for: Inclusion Complexations of Levamisole and Cocaine With Molecular Baskets. Fluorescence Displacement Assay for Detection of Cocaine and Its Adulterant Levamisole in Aqueous Media
Source: Chemistry. 2026 Jun 3;32(41):e71216. doi: 10.1002/chem.71216 (PMC13729933; doi:10.1002/chem.71216)
Supplement: Supplementary file 1 [file CHEM-32-e71216-s001.pdf]

Inclusion Complexations of Levamisole and Cocaine with Molecular Baskets. Fluorescence Displacement Assay for Detection of Cocaine and its Adulterant Levamisole in Aqueous Media

Christopher T. Mortensen,[a][b] Carson E. Ward,[a][b] Nitesh Kumar,[a] Logan Willemann,[a] Ryan Lutz,[a] Kailey Hughes,[a] Zheng Zheng,[a] and Jovica D. Badjić*[a]

[a]Department of Chemistry and Biochemistry

The Ohio State University

100 West 18th Avenue, Columbus, OH 43210 (USA)

E-mail:badjic.1@osu.edu

[b]Equal Contribution to the Study

Supporting Information

General Information

All chemicals were purchased from commercial sources and used as received unless stated otherwise. All solvents were dried before use according to standard literature procedures. Chromatographic purifications were performed with silica gel 60 (SiO_2, 40–75 µm, 200 × 400 mesh). Thin-layer chromatography was performed on a silica gel plate w/UV254 (200 µm). Chromatograms were visualized by UV light or stained with ninhydrin, iodine (I_2), or ceric ammonium sulfate (CAM). All NMR experiments were performed with Bruker 400, 600, and 850 MHz spectrometers. Deuterated solvents and $(CD_3)_2SO$ and D_2O were acquired from Cambridge Isotope Laboratories. NMR data was processed and analyzed using Bruker software (Topspin 4.2, Dynamics center 2.8, MestReNova 13.3.3). High-resolution mass data (HRMS) were obtained using electrospray ionization (ESI) techniques (Bruker ESI TOF and Thermo-fisher ESI orbitrap instruments). Acidity (pH) measurements were completed with an HI 2210 pH meter. For isothermal titration calorimetry (ITC) measurements, we used Nano ITC from TA instruments. UV-Vis spectra were recorded with Shimadzu UV-2401PC spectrophotometer using a 1x1 cm quartz cuvette. Fluorescence spectra were recorded with Cary Eclipse Fluorescence spectrophotometer using a 1x1 cm quartz cuvette.

Synthetic Procedures and Characterizations

For synthesis and characterization of basket **1**, see Badjic and coworkers, *J. Am. Chem. Soc.* **2017**, *139*, 18496−18499. For synthesis and characterization of basket **5** (below), see Badjic and coworkers, *Angew. Chem. Int. Ed.* **2024**, e202420574.

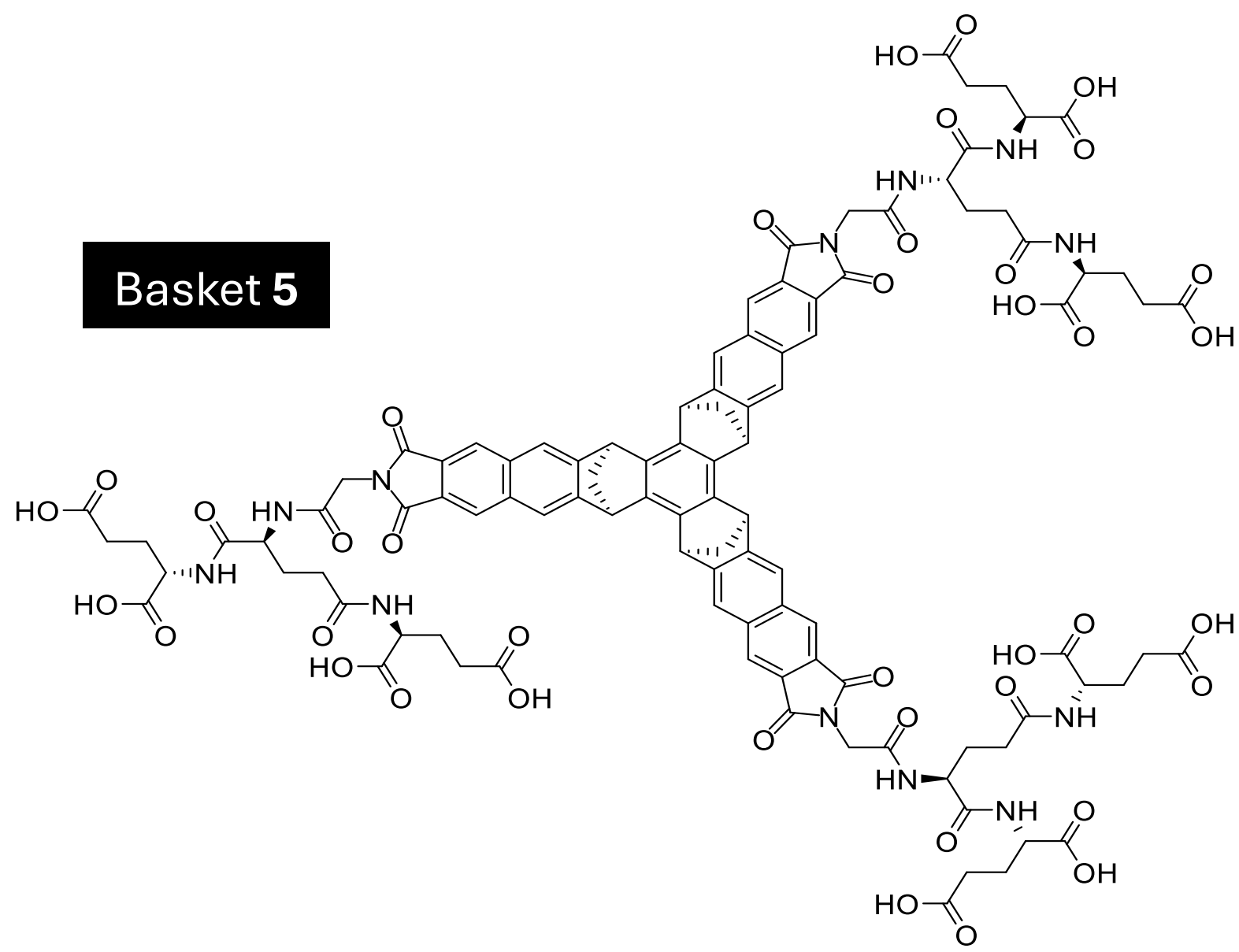

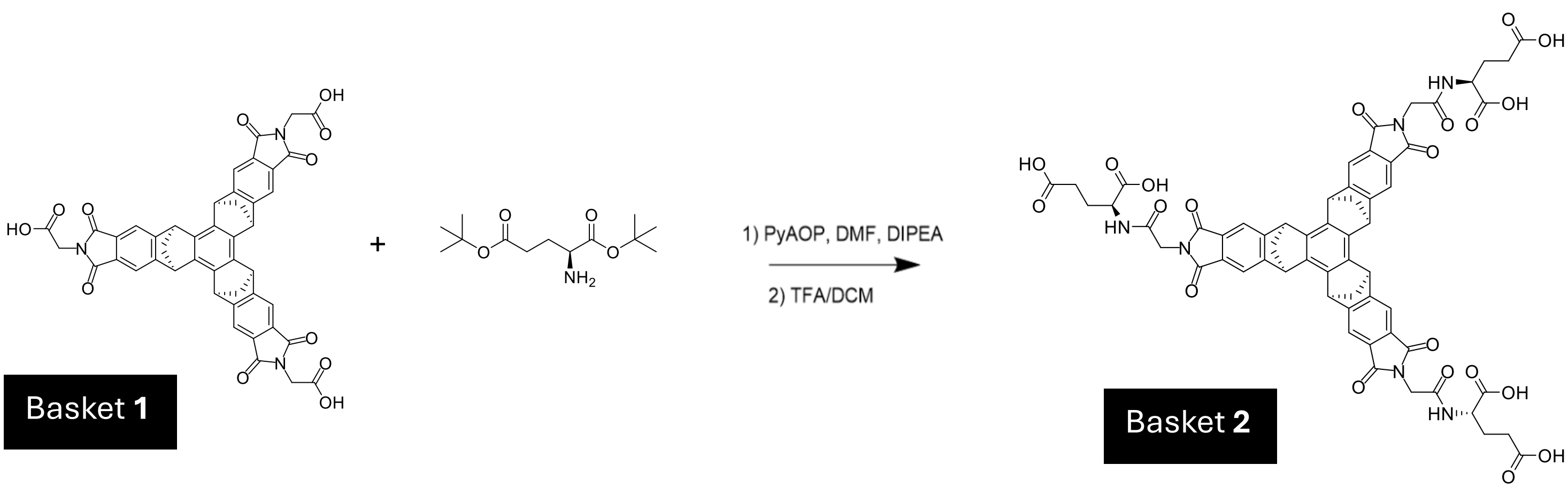

Scheme S1. Synthesis of basket **2**. For the preparation of basket **1**, see Badjic and coworkers, *J. Am. Chem. Soc.* **2017**, *139*, 18496−18499.

Basket 2: Basket **1** (25 mg, 31.18 µmol), *S*-glutamic acid di-*tert*-butyl ester (48.52 mg, 187.1 µmol), and PyAOP (97.6 mg, 187.1 µmol) were placed in a glass vial and kept under inert nitrogen atmosphere. Dry DMF (2 mL) was added and stirred to dissolve the solids followed by the addition of diisopropylethylamine (DIPEA) (65.18 µL, 374.2 µmol). After stirring the reaction mixture for 24 h at 25 ^{o}C, DMF was removed under reduced pressure with the remaining solid dissolved in 2 mL of dichloromethane and subsequently washed with 4 mL of water. The organic layer was dried

with anhydrous Na_2SO_4, concentrated under reduced pressure, and the residual subjected to column chromatography (SiO_2, dichloromethane : acetone = 3:1 to 1:1) to give *t*-Boc protected basket **2** (34.3 mg, 72%) as a colorless oil. This compound (29 mg, 19.01 µmol) was then dissolved in 2 mL of dichloromethane : trifluoroacetic acid = 1:1 and the reaction mixture stirred overnight at 25 ºC. After, the solvent was removed under reduced pressure. The solid residue was suspended in 2 mL of water and treated with 3 mL of 1M HCl to be subjected to centrifugation. The precipitate was removed and treated with 2 mL of 0.1M HCl followed by centrifugation. The precipitate was removed and lyophilized to give basket **2** (17.5 mg, 77%) as an off-white solid. ^{1}H NMR (400 MHz, DMSO-d_6) δ (ppm) = 8.46 (d, J = 8.0 Hz, 3H), 7.82 (s, 6H), 4.73 (s, 6H), 4.22-4.17 (m, 3H), 4.13-3.99 (m, 6H), 2.29-2.20 (m, 6H), 1.99-1.89 (m, 3H), 1.76-1.65 (m, 3H).). ^{13}C NMR (100 MHz, DMSO-d_6) δ (ppm) = 174.1, 173.4, 167.9, 167.8, 166.6, 158.1, 138.3, 130.3, 116.5, 52.7, 48.7, 30.2, 26.8. HRMS (ESI-MS) *m/z* calculated for $C_{60}H_{48}N_6O_{21}$, $[M+NH_4]^+$: 1206.3211; found: 1206.3209.

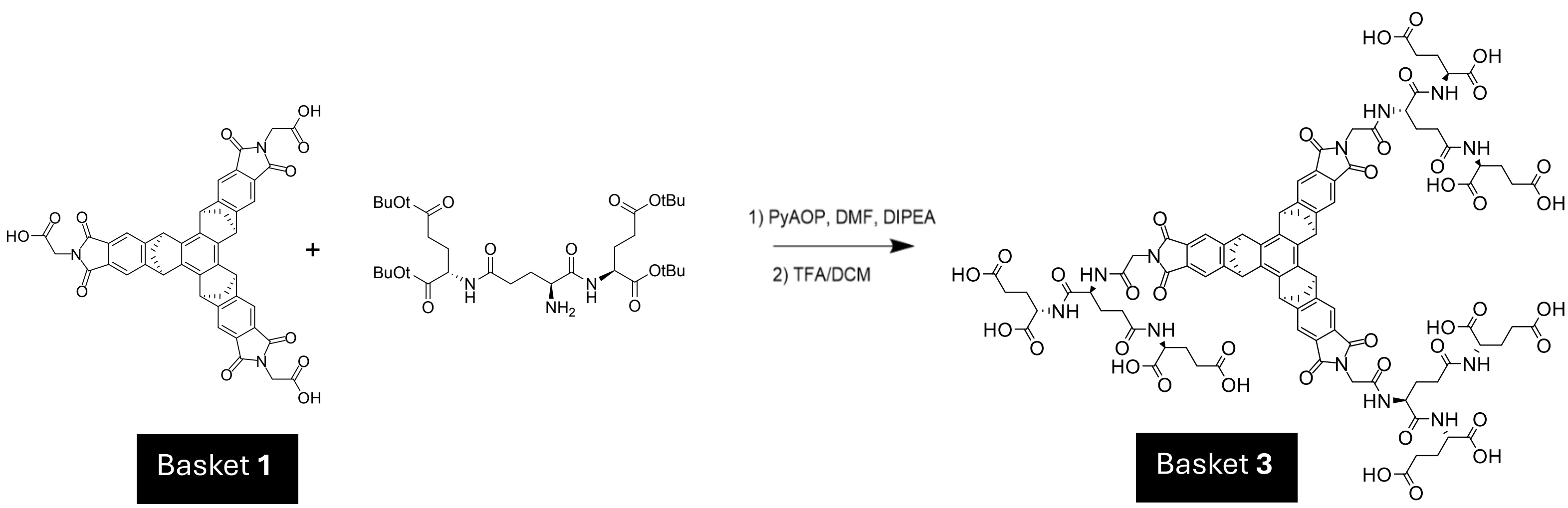

Scheme S2. Synthesis of basket **3**. For the preparation of Glu-Glu$_2$, see Badjic and coworkers, *Angew. Chem. Int. Ed.* **2024**, e202420574.

Basket 3: Basket **1** (25 mg, 31.18 µmol), Glu-Glu$_2$ dendron (124.65 mg, 187.1 µmol), and PyAOP (97.6mg, 187.1 µmol) were placed in a glass vial and kept under inert nitrogen atmosphere. Dry DMF (2 mL) was added and the solution stirred to dissolve the solids followed by the addition of diisopropylethylamine (DIPEA) (65.18 µL, 374.2 µmol). After stirring the reaction mixture for 48 h at 25 ºC, DMF was removed under reduced pressure with the remaining solid dissolved in 2 mL of dichloromethane and subsequently washed with 4 mL of water. The organic layer was dried with anhydrous Na_2SO_4, concentrated under reduced pressure, and the residual solid subjected to column chromatography (SiO_2, dichloromethane : acetone = 3:1 to 1:1) to give *t*-Boc protected basket **3** (35.6 mg, 43 %) as a colorless oil. This compound (56 mg, 19.01 µmol) was then dissolved in 2 mL of dichloromethane : trifluoroacetic acid = 1:1 and the reaction mixture stirred overnight at 25 ºC. After, the solvent was removed under reduced pressure. The solid residue was suspended in 2 mL of water and treated with 3 mL of 1M HCl, to be subjected to centrifugation. The precipitate was removed and treated with 2 mL of 0.1M HCl followed by centrifugation. The solid precipitate was removed and lyophilized to give basket **3** (16.4 mg, 39 %) as an off-white solid.

^{1}H NMR (850 MHz, DMSO-d_6) δ (ppm) = 8.39 (d, J = 6.8 Hz, 3H), 8.20 (d, J = 7.48 Hz, 3H), 8.0 (d, J = 7.7 Hz, 3H), 7.79 (d, J = 2.2 Hz, 6H), 4.71 (s, 6H), 4.25 (m, 3H), 4.18-4.16 (m, 6H), 4.08-4.05 (m, 6H), 2.29-2.22 (m, 16H), 2.16-2.11 (m, 16H), 2.08 (m, 6H), 1.96-1.92 (m, 8H), 1.88-1.84 (m, 4H), 1.78-1.74 (m, 8H), 1.69-1.66 (m, 4H). ^{13}C NMR (214 MHz, DMSO-d_6) δ (ppm) = 174.1, 174.1, 173.9, 173.6, 172.2, 171.6, 168.0, 167.9, 166.4, 158.1, 138.3, 130.4, 116.6, 65.6, 52.4, 51.6, 51.5, 48.7, 34.6, 31.9, 31.7, 30.5, 30.4, 28.8, 26.7, 26.6. HRMS (ESI-MS) *m*/*z* calculated for $C_{90}H_{90}N_{12}O_{39}$, $[M+NH_4]^{2+}$: 1981.5797; found: 1981.5826.

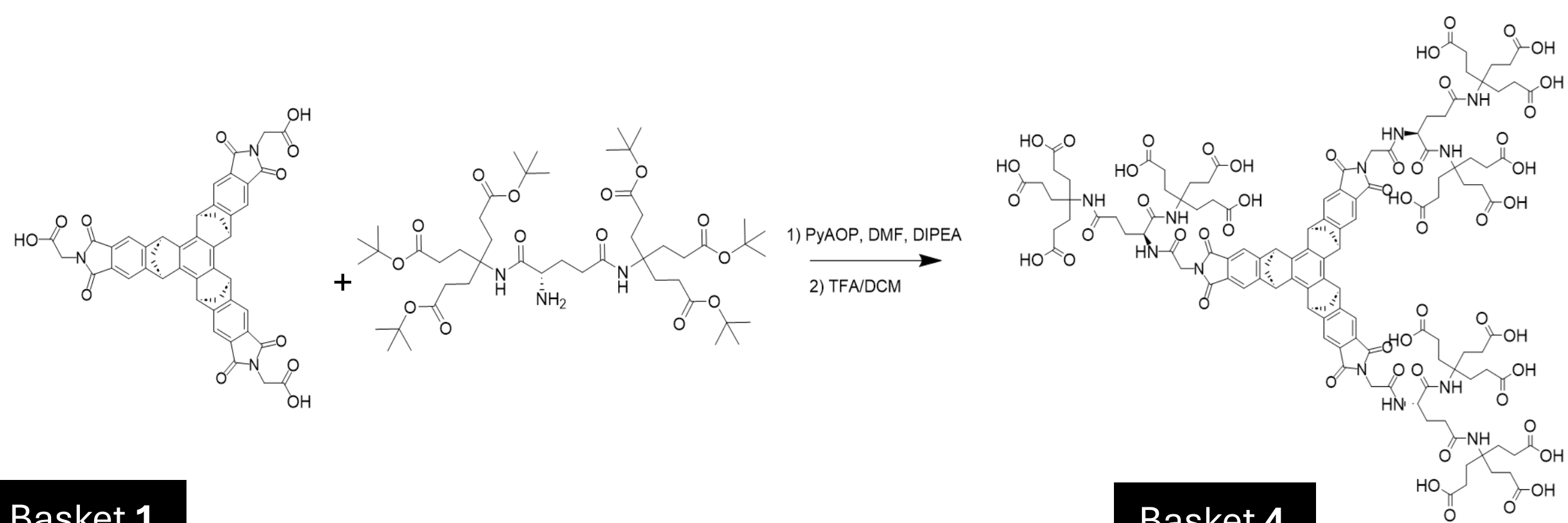

Basket **1**

Basket **4**

Scheme S3. Synthesis of basket **4**. For the preparation of Glu-D_2, see Badjic and coworkers, *Angew. Chem. Int. Ed.* **2025**, e202514676.

asket 4: Basket **1** (10 mg, 12.47 µmol), Glu-D_2 dendron (70.52 mg, 74.84 µmol), and PyAOP (39.02mg, 74.84 µmol) were placed in a glass vial and kept under inert nitrogen atmosphere. Dry DMF (2 mL) was added, and the solution stirred to dissolve the solids followed by the addition of diisopropylethylamine (DIPEA) (26.07 µL, 149.68 µmol). After stirring the reaction mixture for 48 h at 25 °C, DMF was removed under reduced pressure with the remaining solid dissolved in 2 mL of dichloromethane and subsequently washed with 4 mL of water. The organic layer was dried with anhydrous Na_2SO_4, concentrated under reduced pressure, and the residual subjected to column chromatography (SiO_2, dichloromethane : acetone = 5:1 to 1:1) to give *t*-Boc protected basket **4** (32.1 mg, 72%) as a colorless oil. This compound (50 mg, 13.99 µmol) was dissolved in 2 mL of dichloromethane : trifluoroacetic acid = 1:1 and the reaction mixture stirred overnight at 25 °C. After, the solvent was removed under reduced pressure. The solid residue was suspended in 2 mL of water and treated with 3 mL of 1M HCl, to be subjected to centrifugation. The solid was collected and treated with 2 mL of 0.1M HCl followed by centrifugation. The remaining precipitate was removed and lyophilized to give basket **4** (23.0 mg, 64%) as a beige solid. ^{1}H NMR (850 MHz, DMSO-d_6) δ (ppm) = 8.29 (d, J = 7.2 Hz, 3H), 7.79 (d, J = 2.0 Hz, 6H), 7.16 (d, J = 15.4 Hz, 6H), 4.71 (s, 6H), 4.13 (d, J = 5.4 Hz, 3H), 4.07 (s, 6H), 2.11-2.09 (m, 46H), 1.82-1.81 (m, 46H). ^{13}C NMR (214 MHz, DMSO-d_6) δ (ppm) = 174.9, 174.8, 171.7, 171.0, 168.0, 167.9, 166.5, 158.0, 138.3, 130.4, 56.9, 56.7, 53.5, 48.7, 32.9, 29.5, 29.4, 28.8, 28.5, 28.4, 27.5. HRMS (ESI-MS) *m*/*z* calculated for $C_{120}H_{138}N_{12}O_{51}$, $[M+NH_4]^{2+}$: 1299.9641; found: 1299.9683.

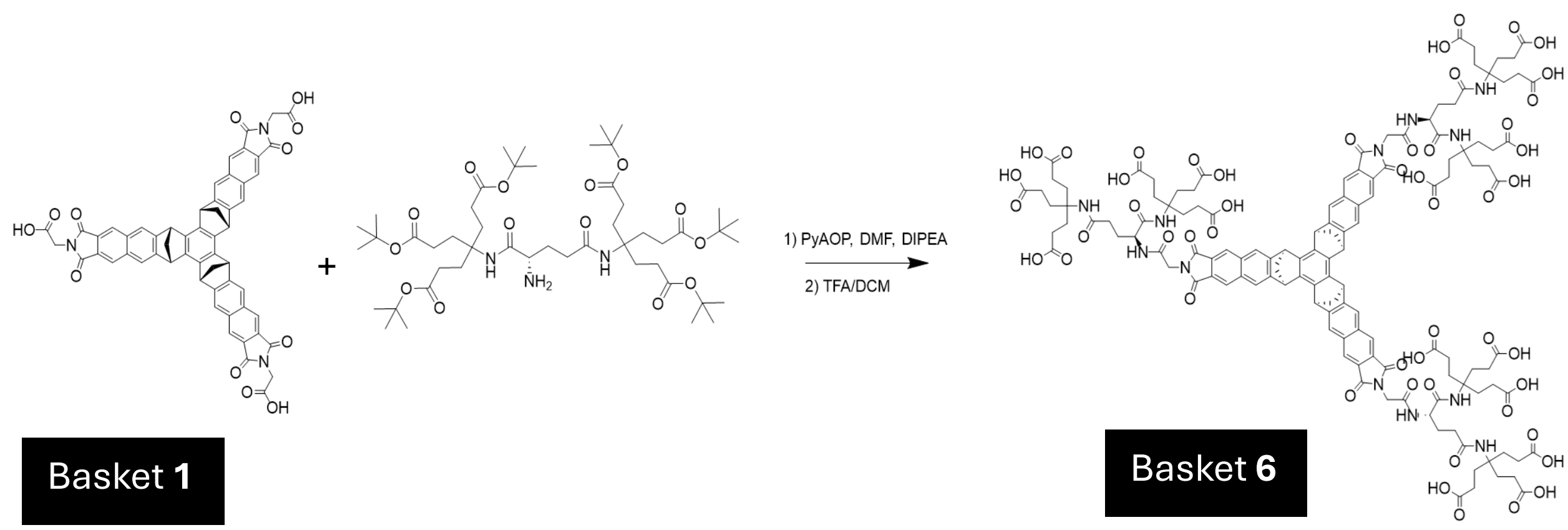

Scheme S4. Synthesis of basket **6**. For the preparation of deep-cavity basket, see Badjic and coworkers, *Chem. Eur. J.* **2023**, e202303374.

Basket 6: Deep-cavity basket (5 mg, 5.25 µmol), Glu-D_2 dendron (29.70 mg, 31.52 µmol), and PyAOP (16.43 mg, 31.52 µmol) were placed in a glass vial and kept under inert nitrogen atmosphere. Dry DMF (2 mL) was added and stirred to dissolve the solids followed by the addition of diisopropylethylamine (DIPEA) (10.98 µL, 63.03 µmol). After stirring the reaction mixture for 48 h at 25 ^oC, DMF solvent was removed under reduced pressure with the solid dissolved in 2 mL of dichloromethane and subsequently washed with 4 mL of water. The organic layer was dried with anhydrous Na_2SO_4, concentrated under reduced pressure, and the residual subjected to column chromatography (SiO_2, dichloromethane : acetone = 5:1 to 2:1) to give *t*-Boc protected basket **6** (15.5 mg, 79%) as a colorless oil. This compound (22 mg, 5.91 µmol) was then dissolved in 2 mL of dichloromethane : trifluoroacetic acid = 1:1 and the reaction mixture stirred overnight at 25 ^oC. After, the solvent was removed under reduced pressure. The solid residue was suspended in 2 mL of water and treated with 3 mL of 1M HCl, to be subjected to centrifugation. The solid was collected and then treated with 2 mL of 0.1M HCl followed by centrifugation. The remaining precipitate was removed and lyophilized to give basket **6** (9.1 mg, 57%) as a beige solid. ^{1}H NMR (850 MHz, DMSO-d_6) δ (ppm) = 8.36 (d, J = 13.5 Hz, 3H), 8.17 (s, 6H), 7.90 (d, J = 6.0 Hz, 6H), 7.15-7.11 (m, 6H), 4.80 (s, 6H), 4.18-4.13 (m, 9H), 2.09 (m, 48H), 1.8 (m, 48H). ^{13}C NMR (214 MHz, DMSO-d_6) δ (ppm) = 174.9, 174.8, 171.7, 171.0, 167.7, 151.1, 138.2, 134.6, 127.4, 124.4, 121.7, 56.9, 56.7, 53.5, 48.3, 32.9, 29.4, 28.7, 28.5, 28.4, HRMS (ESI-MS) *m/z* calculated for $C_{132}H_{144}N_{12}O_{51}$, $[M+NH_4]^{2+}$: 1374.9876; found: 1374.9923.

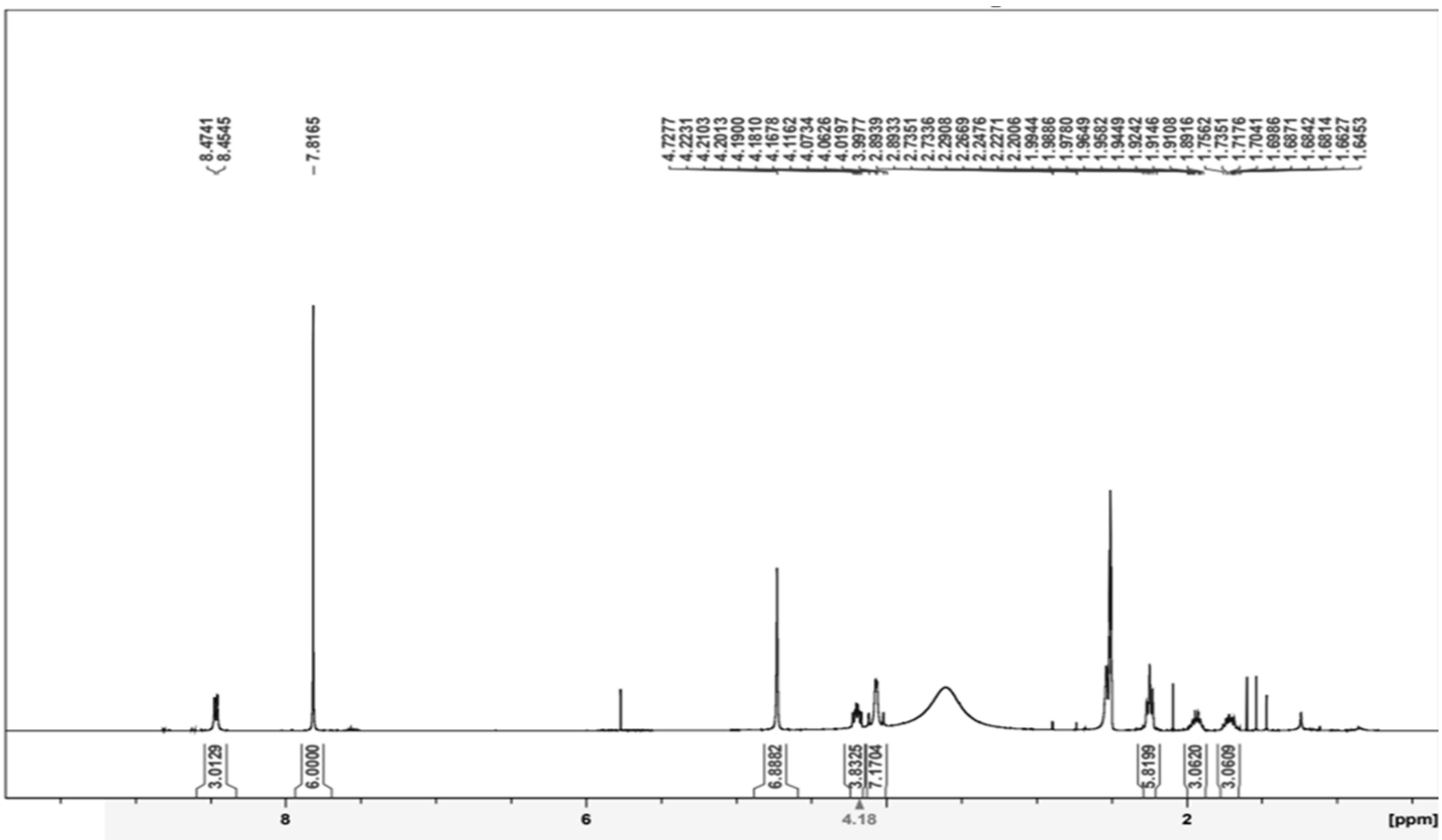

Figure S1. ^{1}H NMR spectrum of basket **2** (400 MHz, 298K, DMSO-d_6).

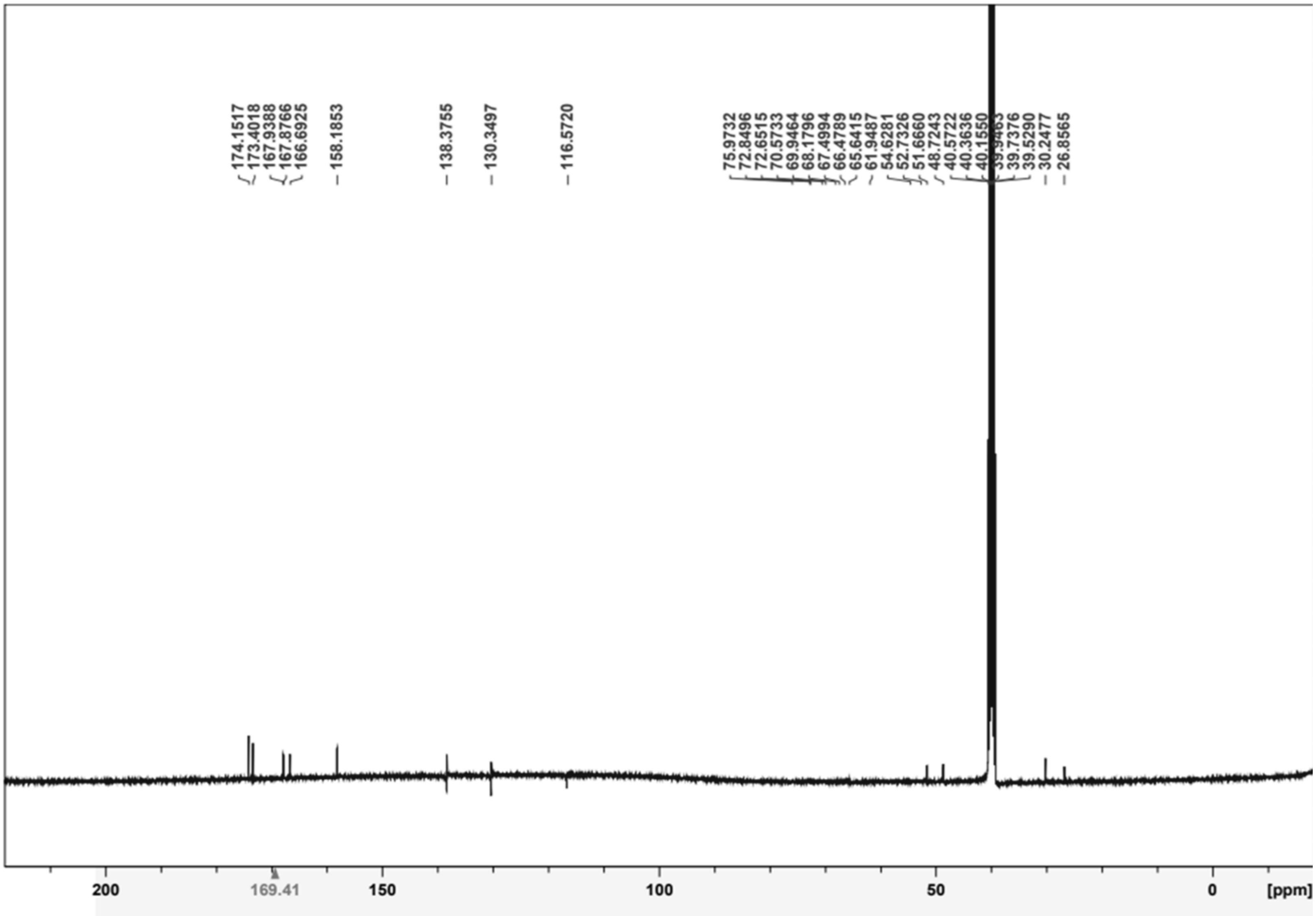

Figure S2. $^{13}C\{^1H\}$ NMR spectrum of basket **2** (100 MHz, 298K, DMSO-d_6).

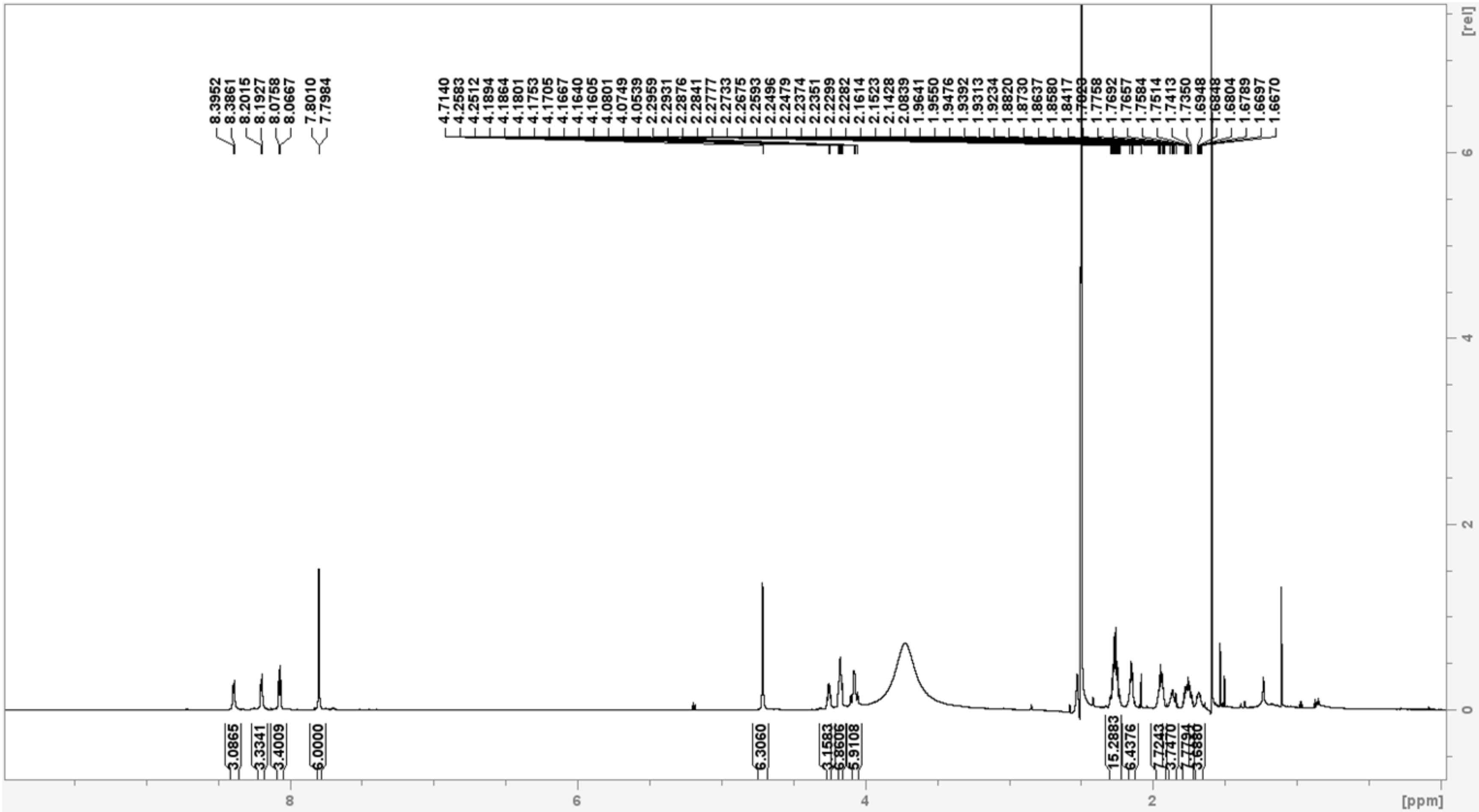

Figure S3. ^{1}H NMR spectrum of basket **3** (850 MHz, 298K, DMSO-d_6).

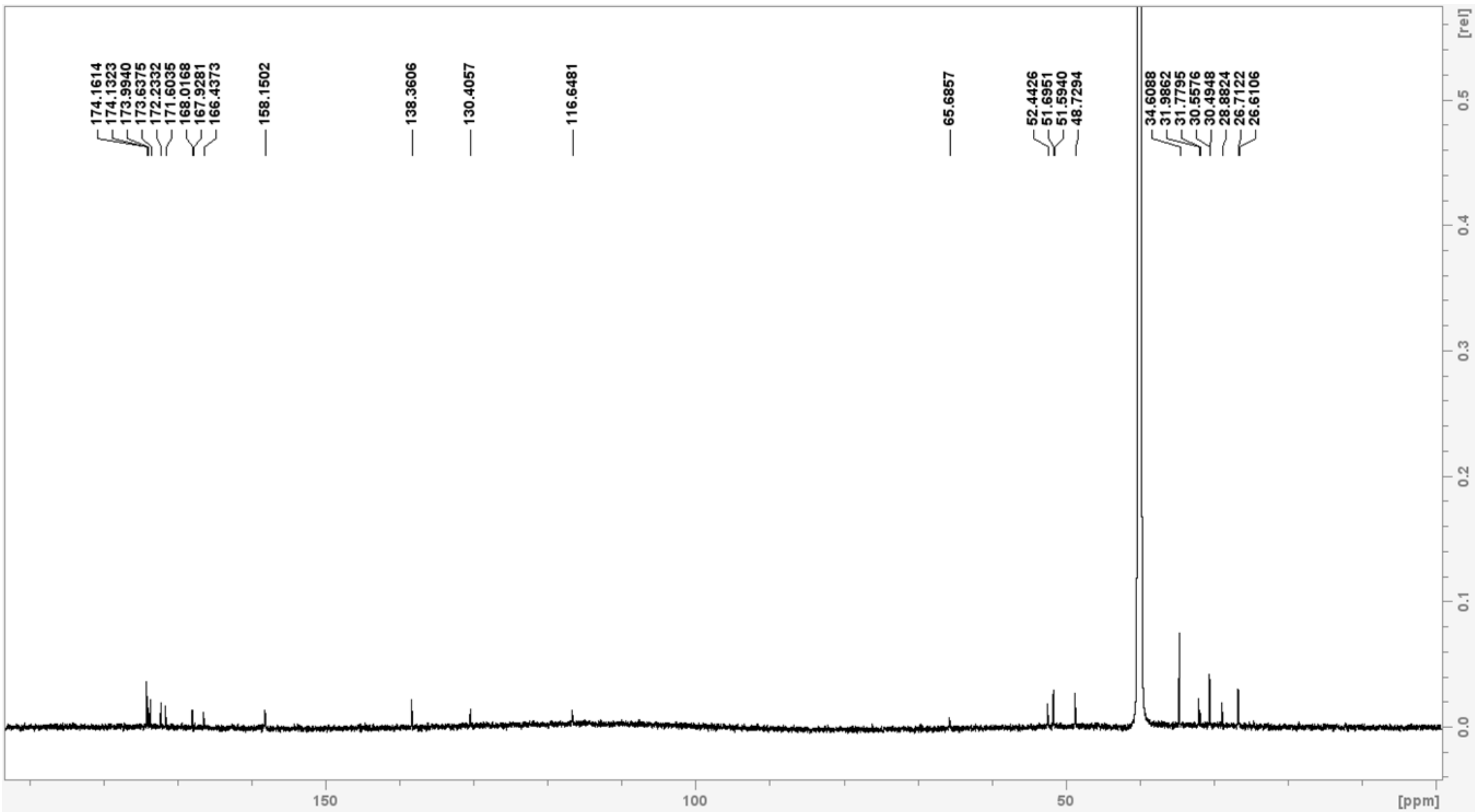

Figure S4. $^{13}C\{^{1}H\}$ NMR spectrum of basket **3** (214 MHz, 298K, DMSO-d_6).

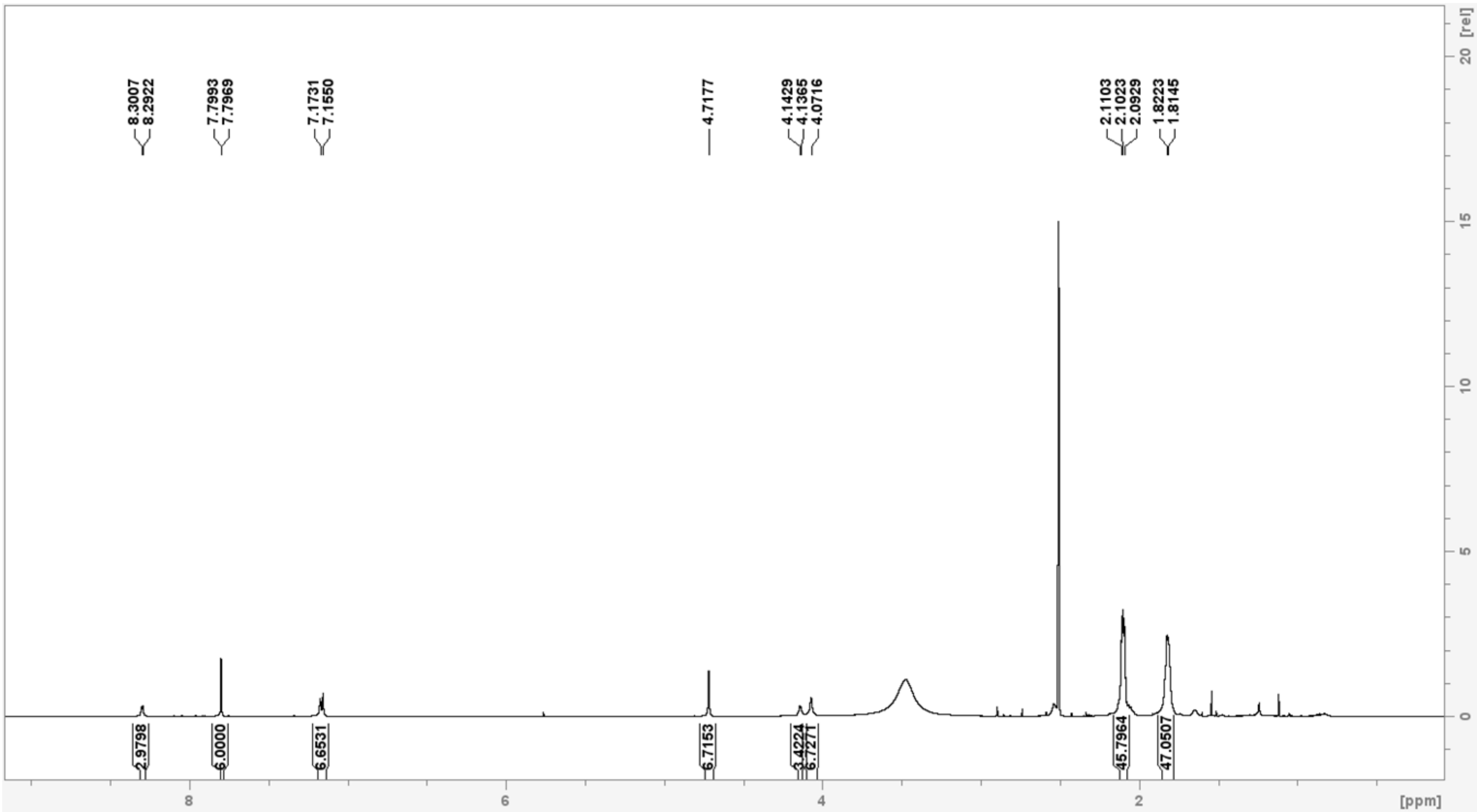

Figure S5. ^{1}H NMR spectrum of basket **4** (850 MHz, 298K, DMSO-d_6).

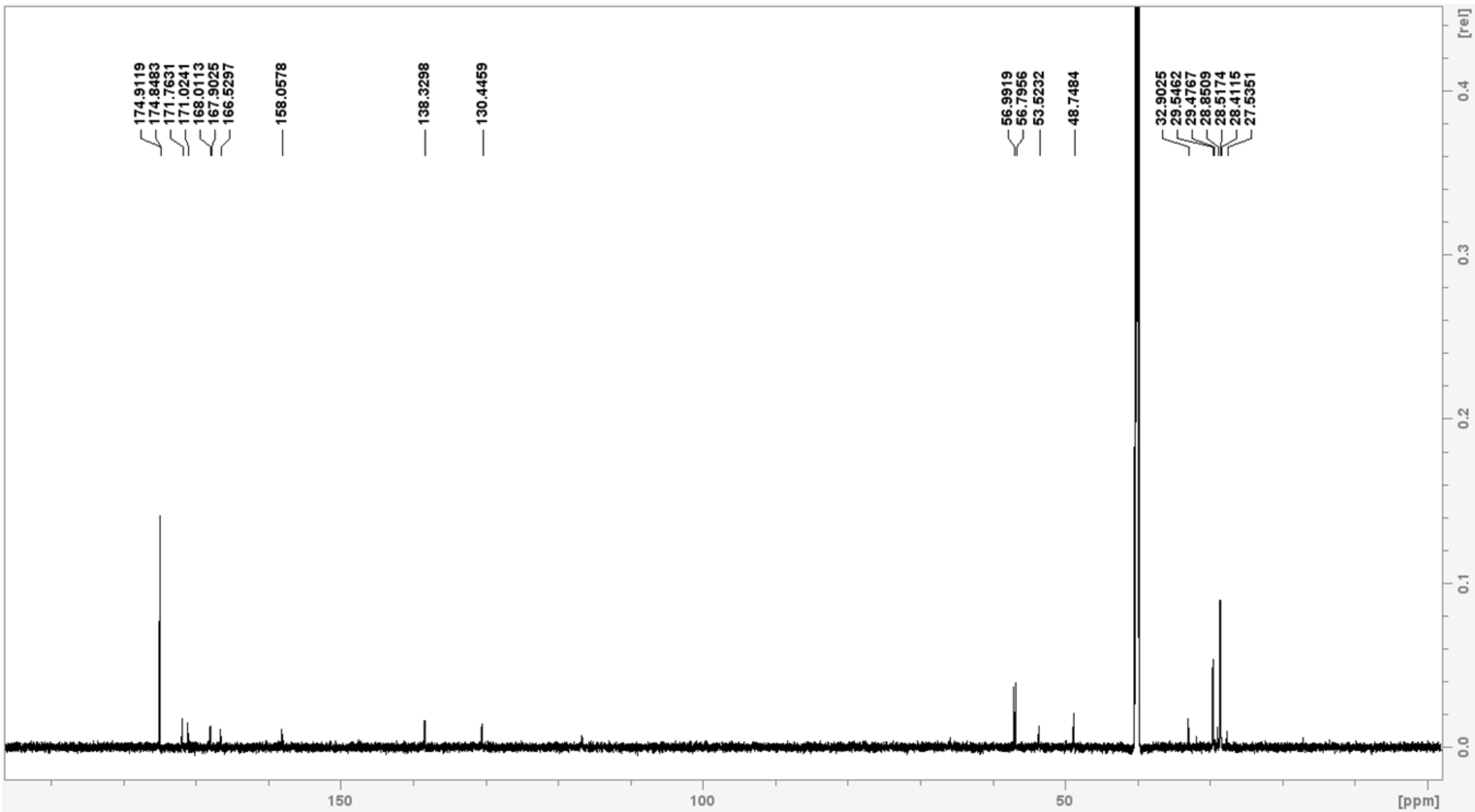

Figure S6. $^{13}C\{^{1}H\}$ NMR spectrum of basket **4** (214 MHz, 298K, DMSO-d_6).

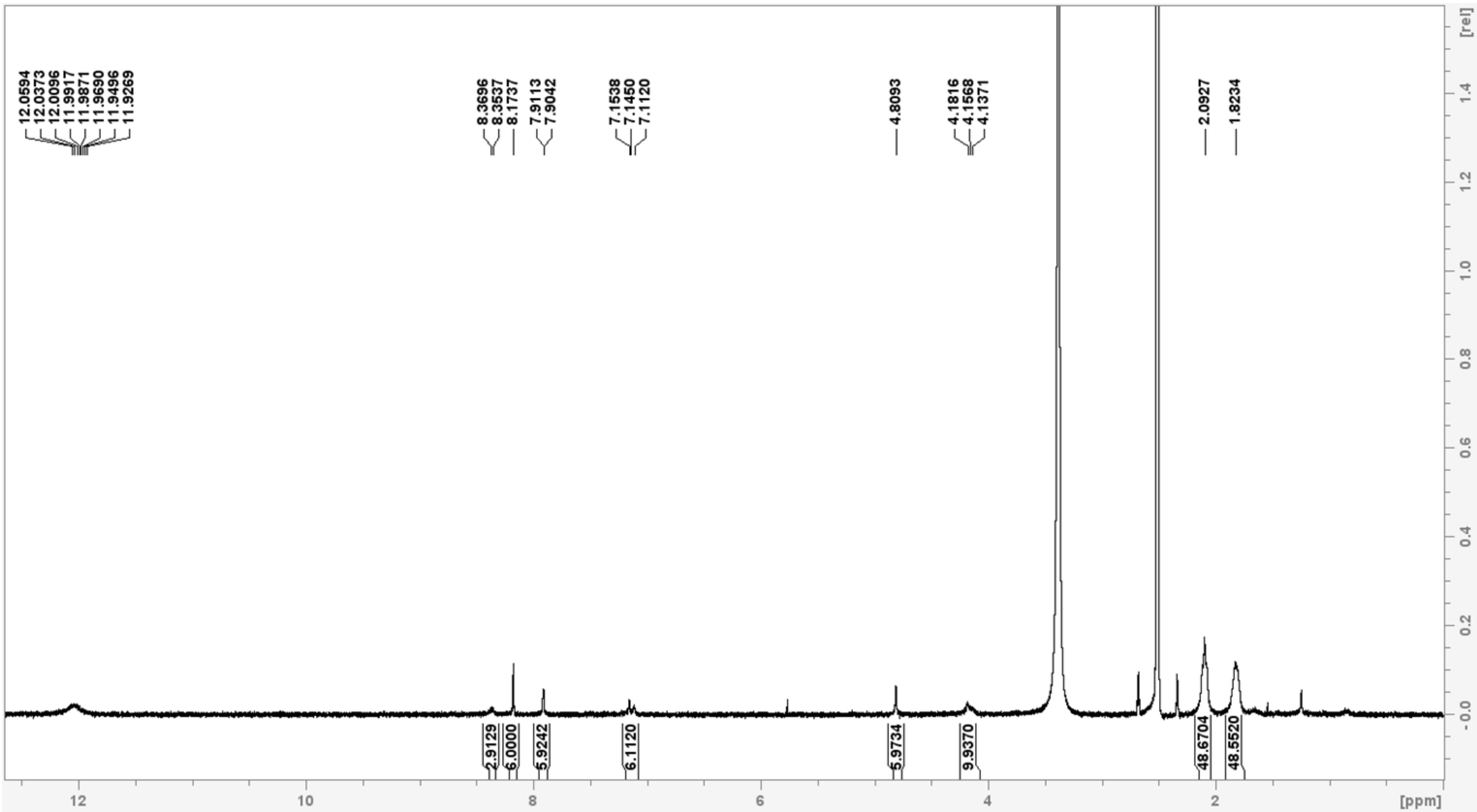

Figure S7. ^{1}H NMR spectrum of basket **6** (400 MHz, 298K, DMSO-d_6).

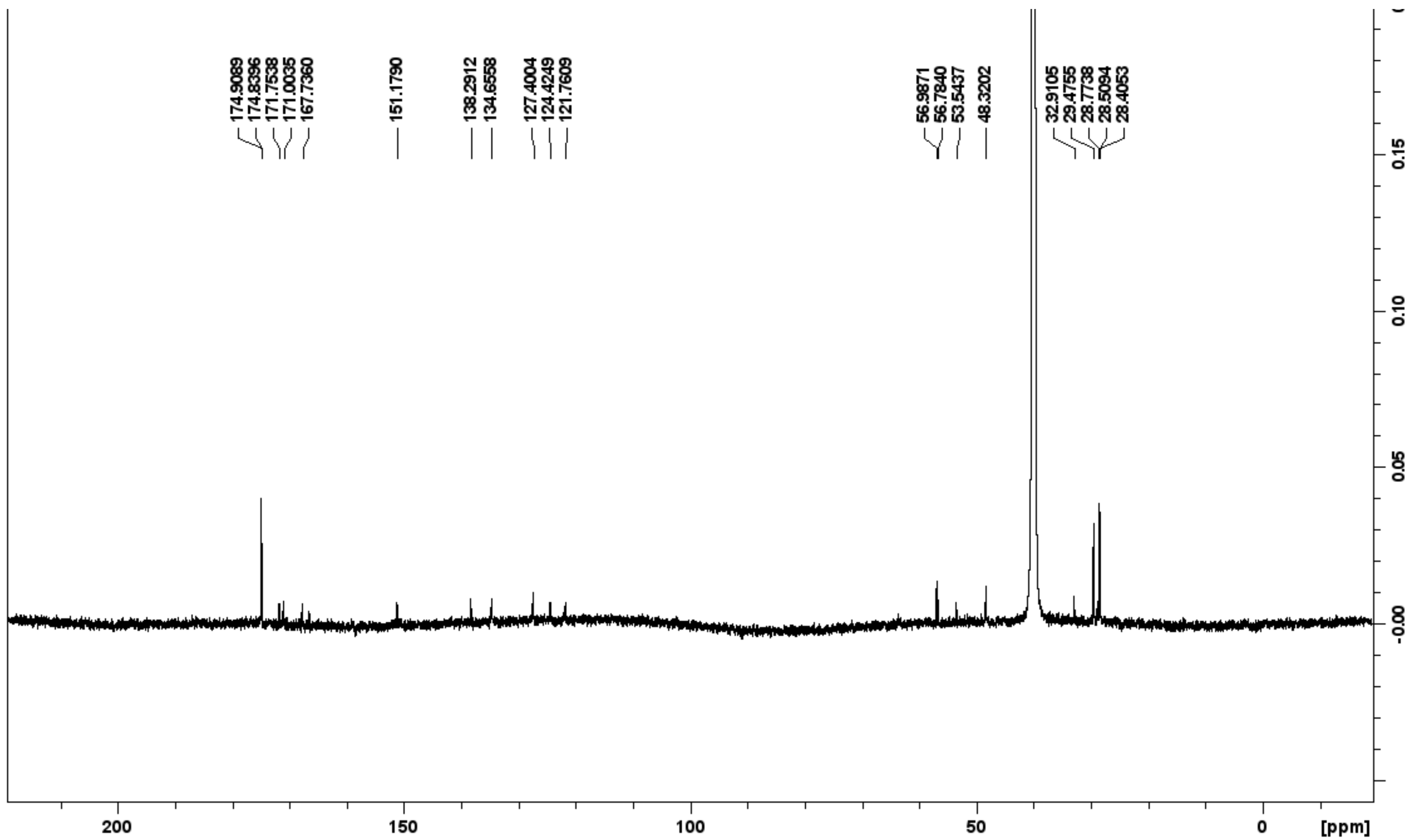

Figure S8. $^{13}C\{^{1}H\}$ NMR spectrum of basket **6** (214 MHz, 298K, DMSO-d_6).

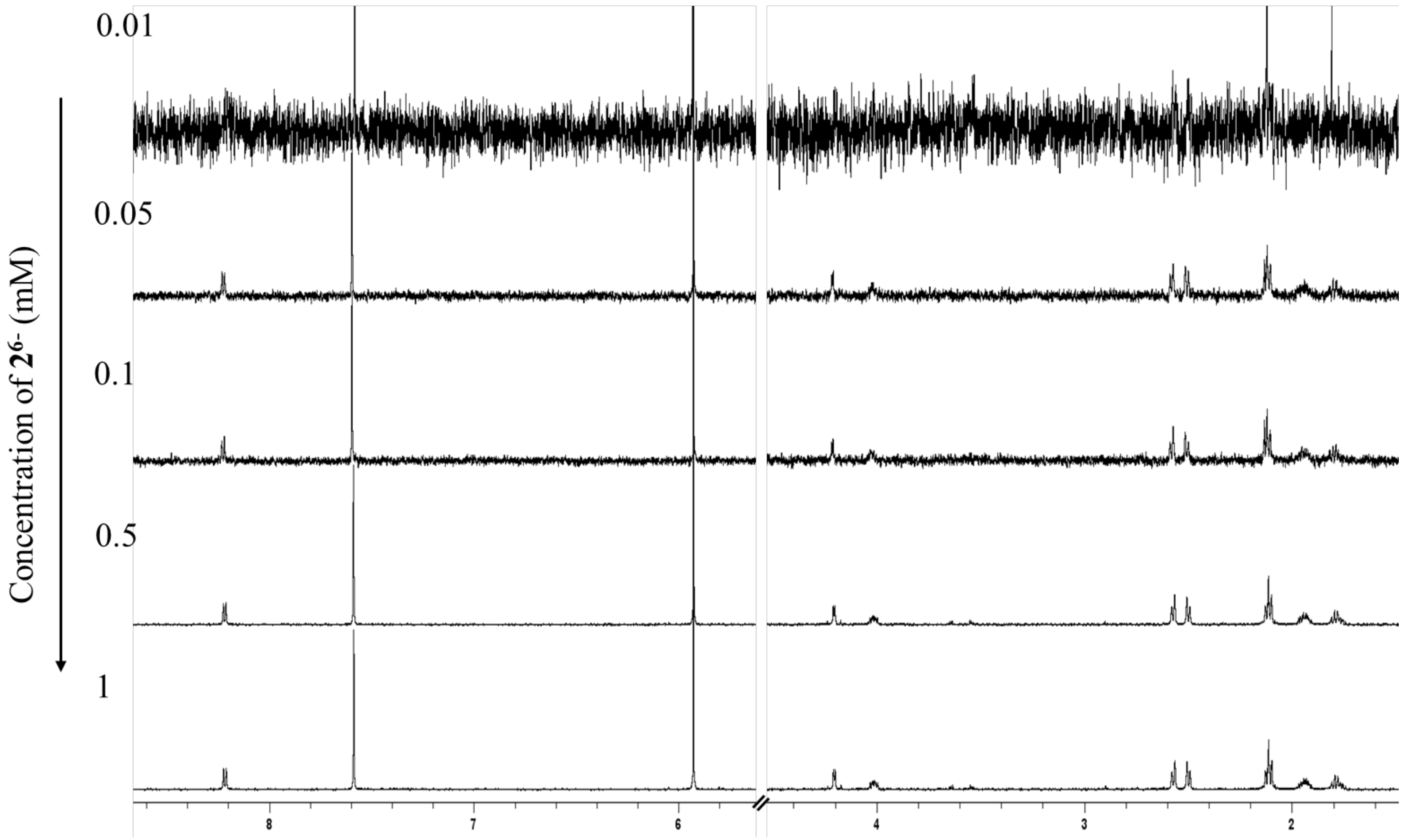

Figure S9. ^{1}H NMR spectra (600 MHz, water suppression) of 0.01-1.0 mM basket **2**$^{6-}$ in 30 mM phosphate buffer at pH = 7.0 (10% D_2O, 298 K).

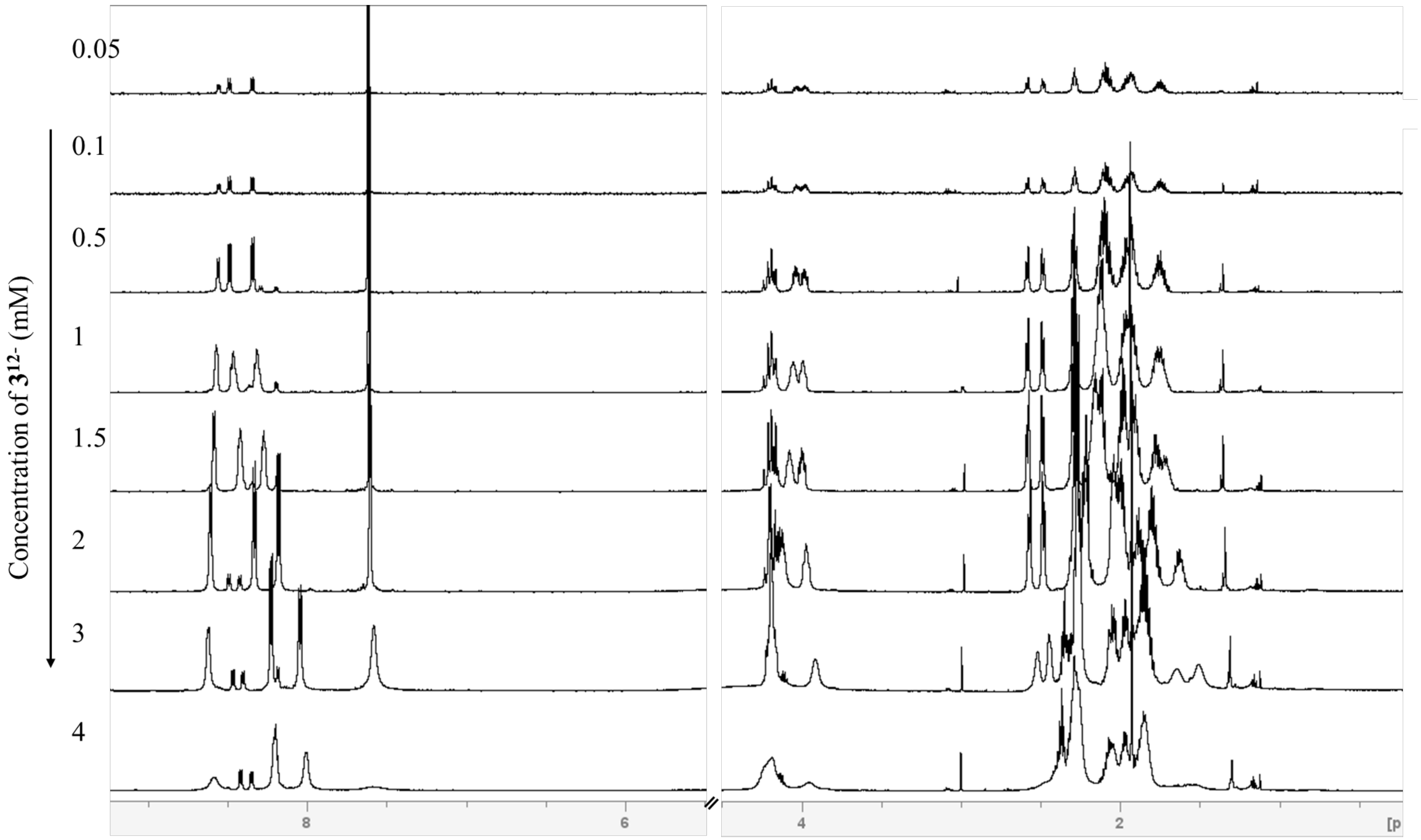

Figure S10. ^{1}H NMR spectra (600 MHz, water suppression) of 0.05-4.0 mM basket **3**$^{12-}$ in 30 mM phosphate buffer at pH = 7.0 (10% D_2O, 298 K). Note that signals (e.g., 8-9 ppm) undergo a negligible shift or change in shape for 0.05 to 0.5 mM solutions of the basket indicating its monomeric state.

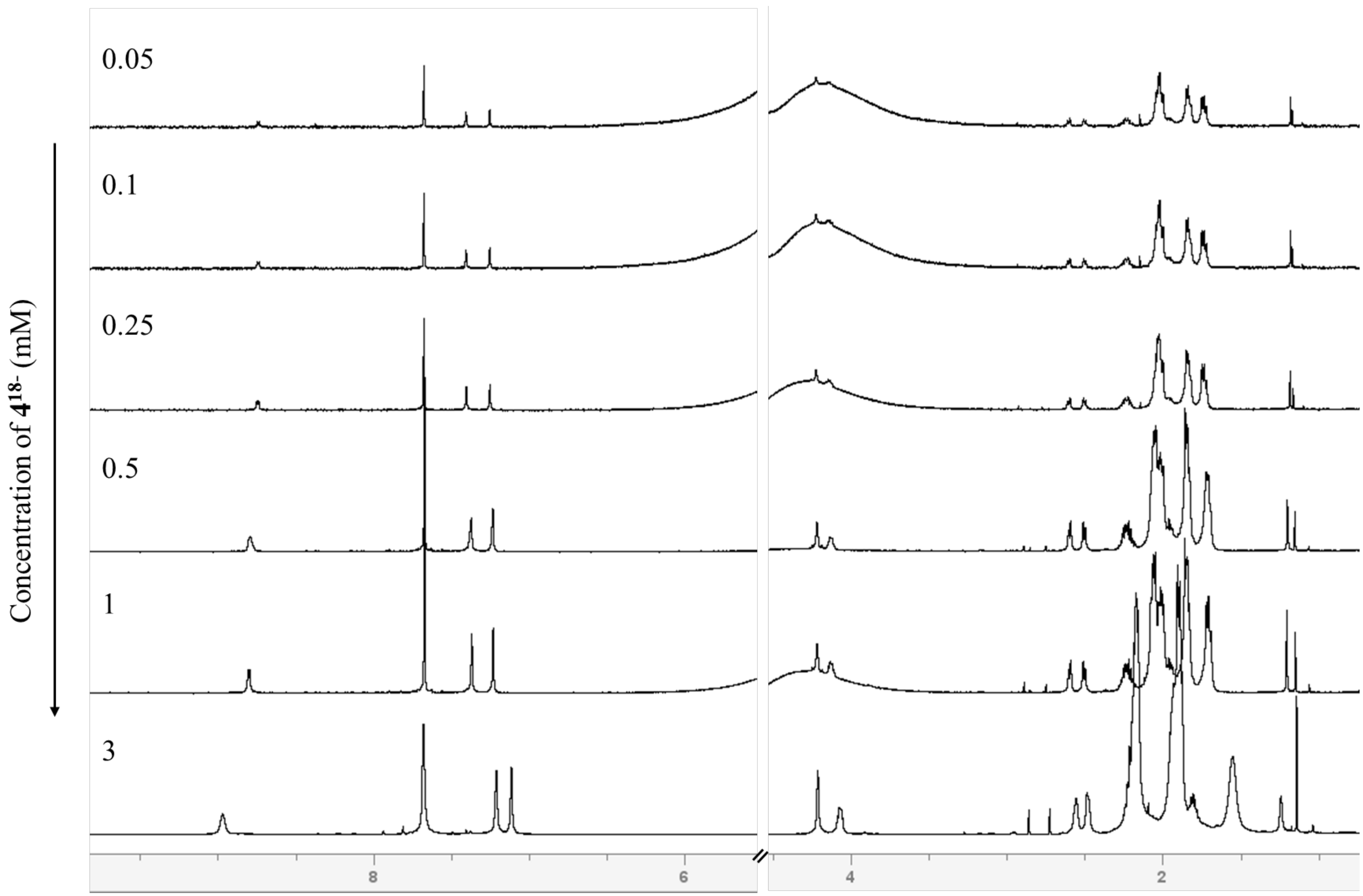

Figure S11. ^{1}H NMR spectra (600 MHz, water suppression) of 0.05-3.0 mM basket **4**$^{18-}$ in 30 mM phosphate buffer at pH = 7.0 (10% D_2O, 298 K). Note that signals (e.g., 7-9 ppm) undergo a negligible shift or change in shape for 0.05 to 0.25 mM solutions of the basket indicating its monomeric state.

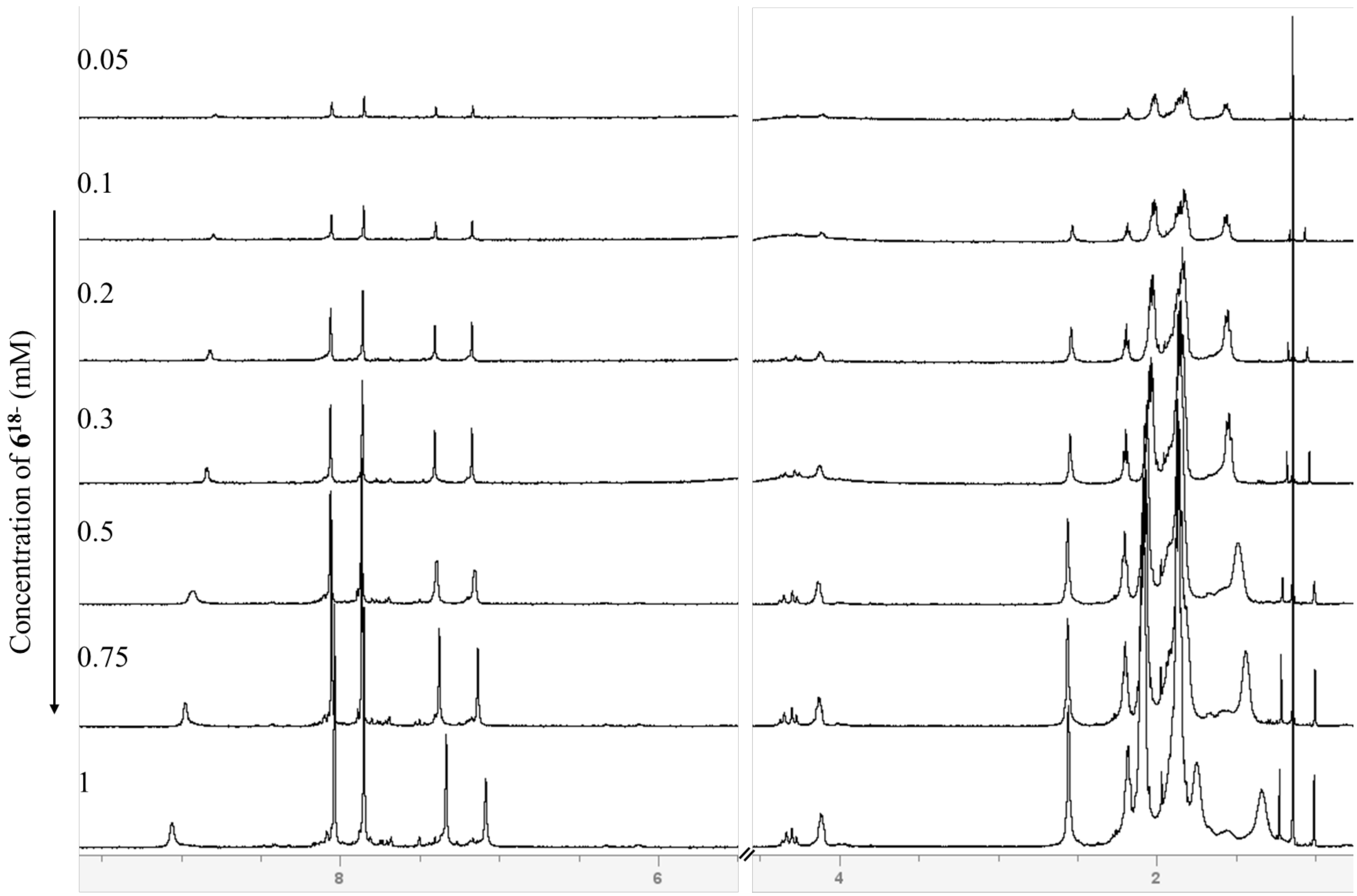

Figure S12. ^{1}H NMR spectra (600 MHz, water suppression) of 0.05-1.0 mM basket $\mathbf{6}^{18-}$ in 30 mM phosphate buffer at pH = 7.0 (10% D_2O) and at 298 K. Note that signals (e.g., 7-9 ppm) undergo a negligible shift or change in shape for 0.05 to 0.2 mM solutions of the basket indicating its predominant monomeric state.

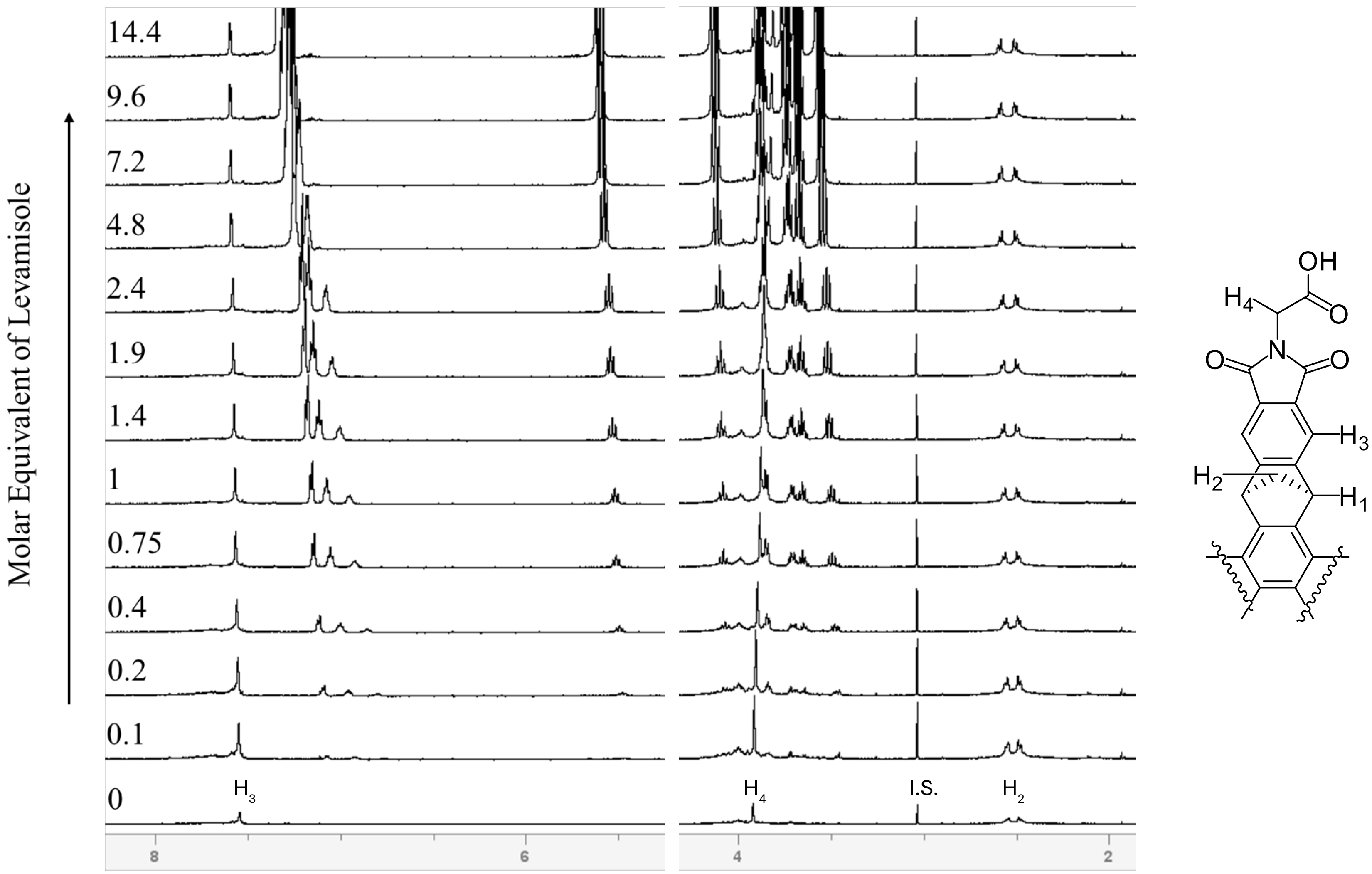

Figure S13. An incremental addition of 100 mM solution of LEV^+ to 0.41 mM basket $\mathbf{1}^{3-}$ (30 mM phosphate buffer at pH = 7.0, 90% H_2O: 10% D_2O) was monitored with ^1H NMR spectroscopy (600 MHz, 298K; water suppression). Molar equivalents of LEV^+ are shown on the left; the signal from H_1 is within the water suppression region. Dimethyl sulfone used as internal standard (I.S., 0.5 mM).

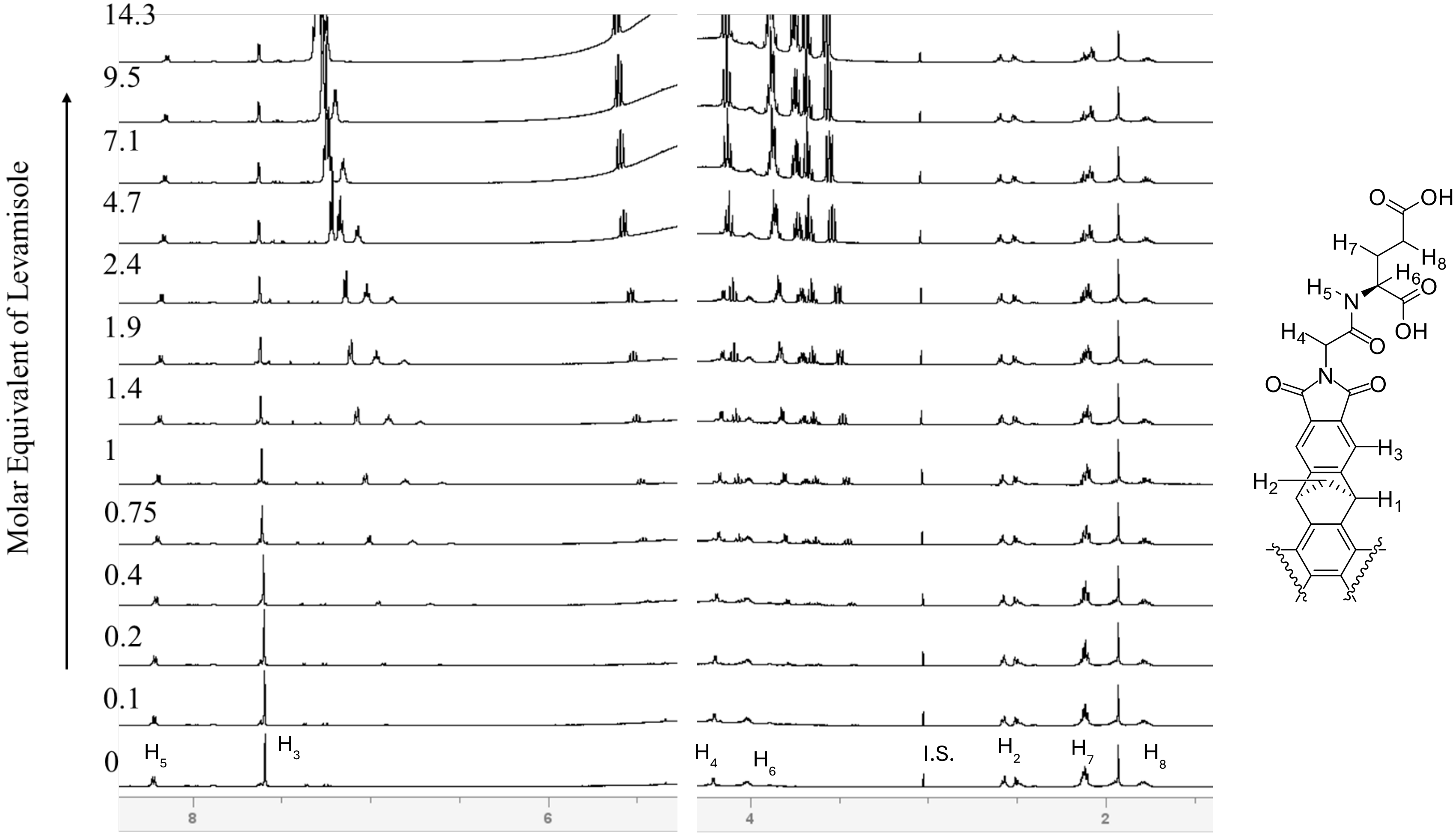

Figure S14. An incremental addition of 100 mM solution of LEV^+ to 0.42 mM basket $\mathbf{2}^{6-}$ (30 mM phosphate buffer at pH = 7.0, 90% H_2O:10% D_2O) was monitored with ^1H NMR spectroscopy (600 MHz, 298K; water suppression). Molar equivalents of LEV^+ are shown on the left; the signal from H_1 is within the water suppression region. Dimethyl sulfone was used as internal standard (I.S., 0.1 mM).

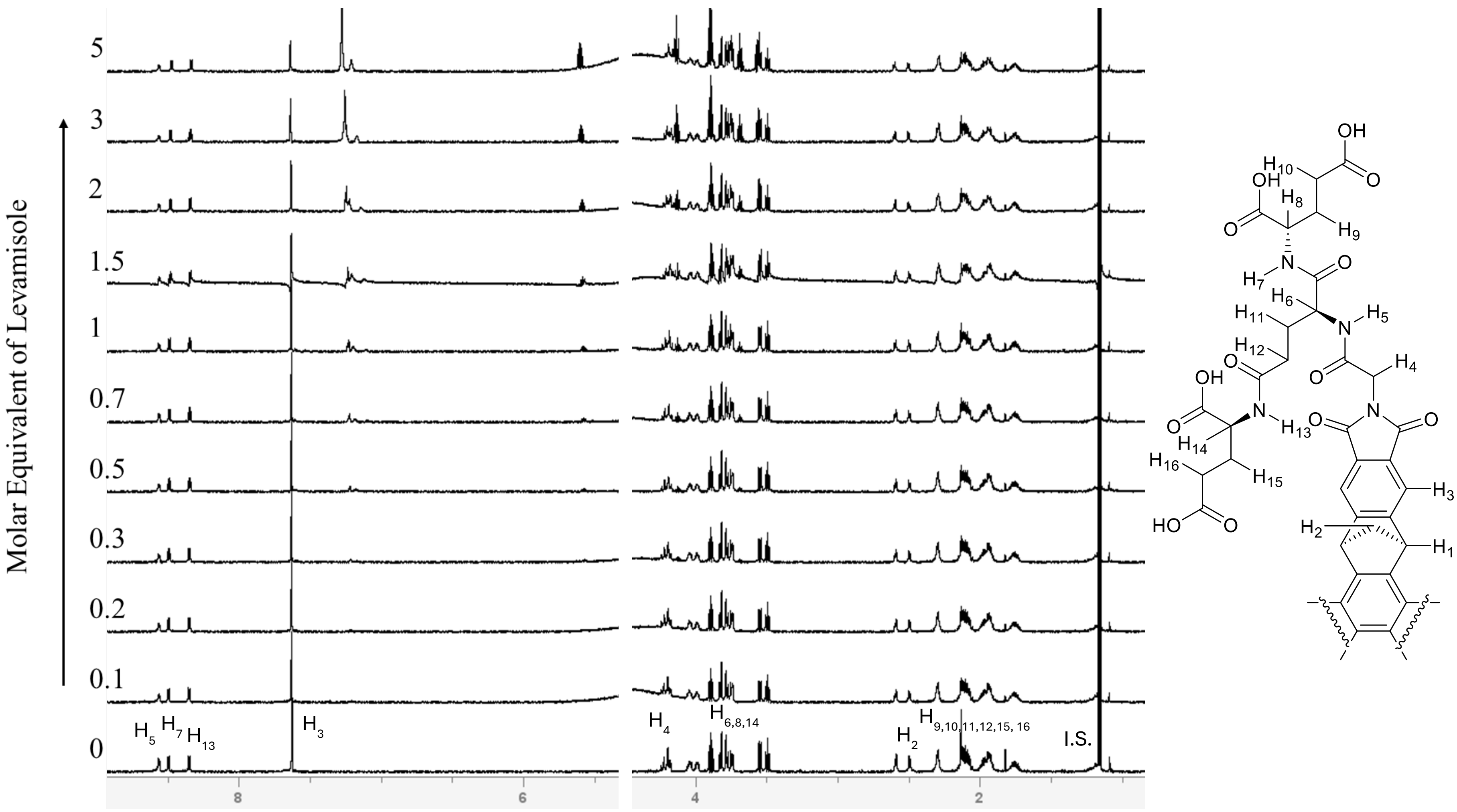

Figure S15. An incremental addition of 1.0 mM solution of LEV^{+} to 50 μM basket $\mathbf{3}^{12-}$ (30 mM phosphate buffer at pH = 7.0, 90% H_2O:10% D_2O) was monitored with ^{1}H NMR spectroscopy (600 MHz, 298K; water suppression). Molar equivalents of LEV^{+} are shown on the left; the signal from H_1 is within the water suppression region. Dimethyl sulfone was used as internal standard (I.S., 0.1 mM).

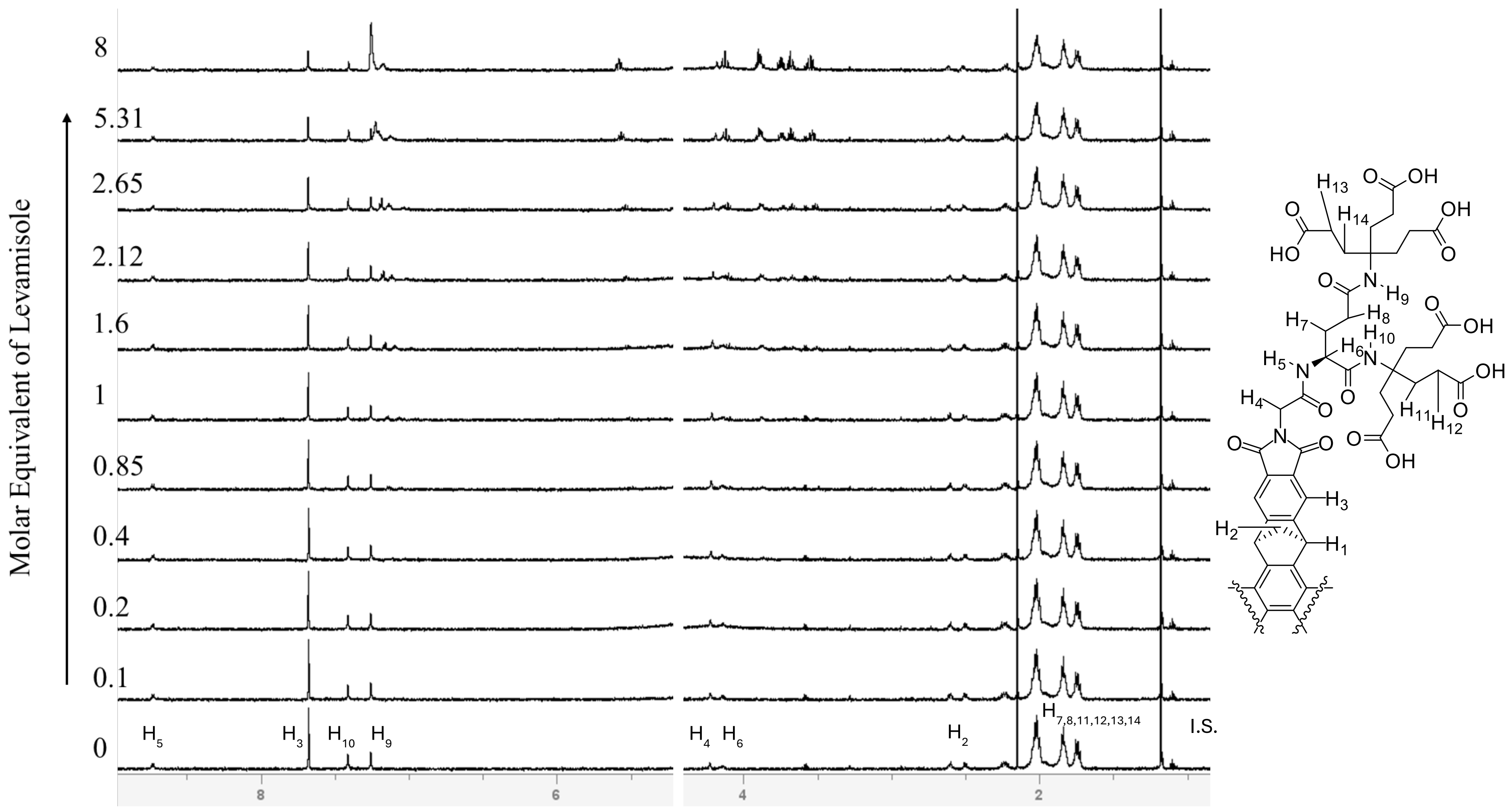

Figure S16. An incremental addition of 0.65 mM solution of LEV^+ to 31 μM basket $\mathbf{4}^{18-}$ (30 mM phosphate buffer at pH = 7.0, 90% H_2O:10% D_2O) was monitored with ^1H NMR spectroscopy (600 MHz, 298K; water suppression). Molar equivalents of LEV^+ are shown on the left; the signal from H_1 is within the water suppression region. Dimethyl sulfone was used as internal standard (I.S., 0.1 mM).

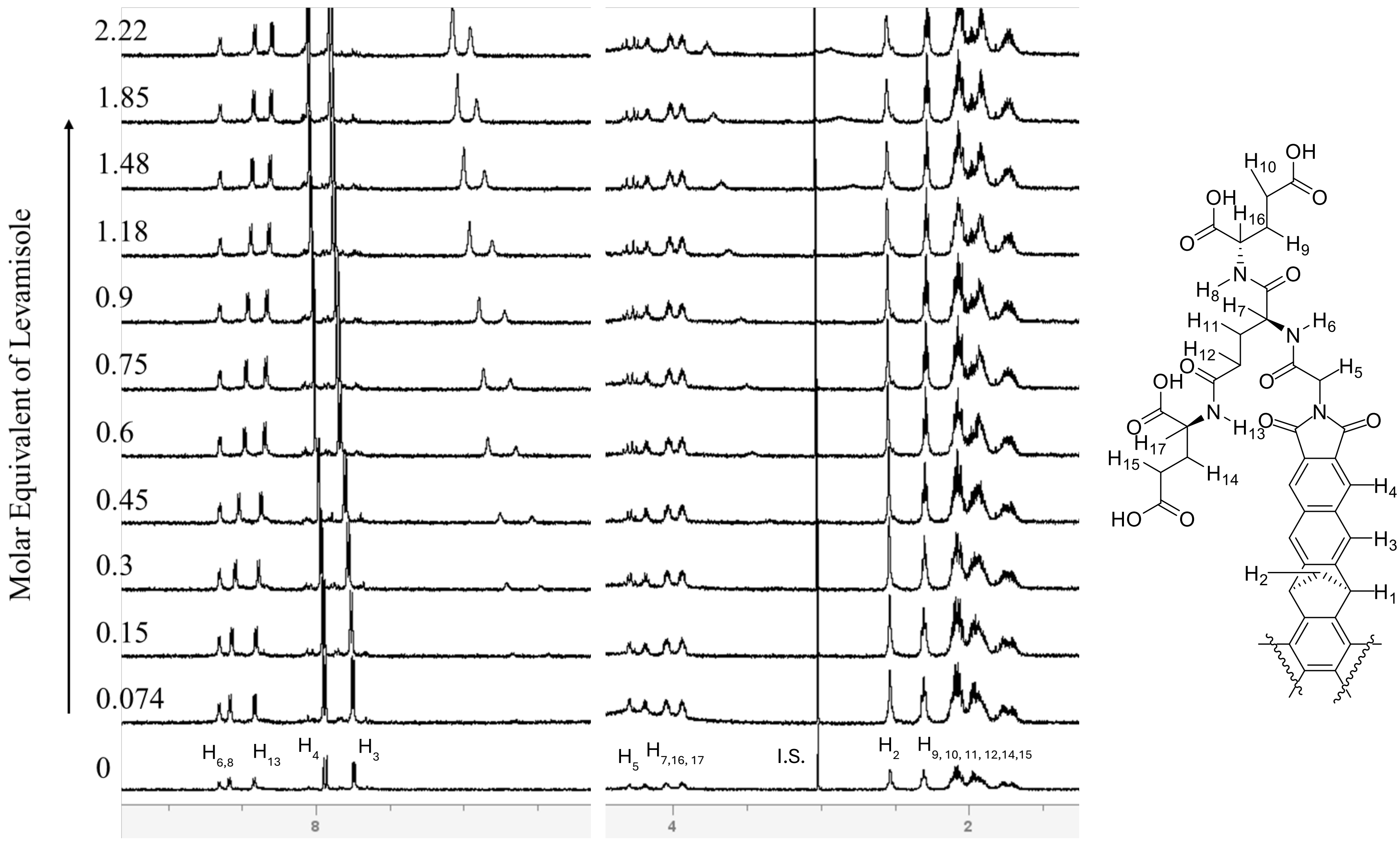

Figure S17. An incremental addition of 25 mM solution of LEV^+ to 67 µM basket $\mathbf{5}^{12-}$ (30 mM phosphate buffer at pH = 7.0, 90% H_2O:10% D_2O) was monitored with ^1H NMR spectroscopy (600 MHz, 298K; water suppression). Molar equivalents of LEV^+ are shown on the left; the signal from H_1 is within the water suppression region. Dimethyl sulfone was used as internal standard (I.S., 0.1 mM).

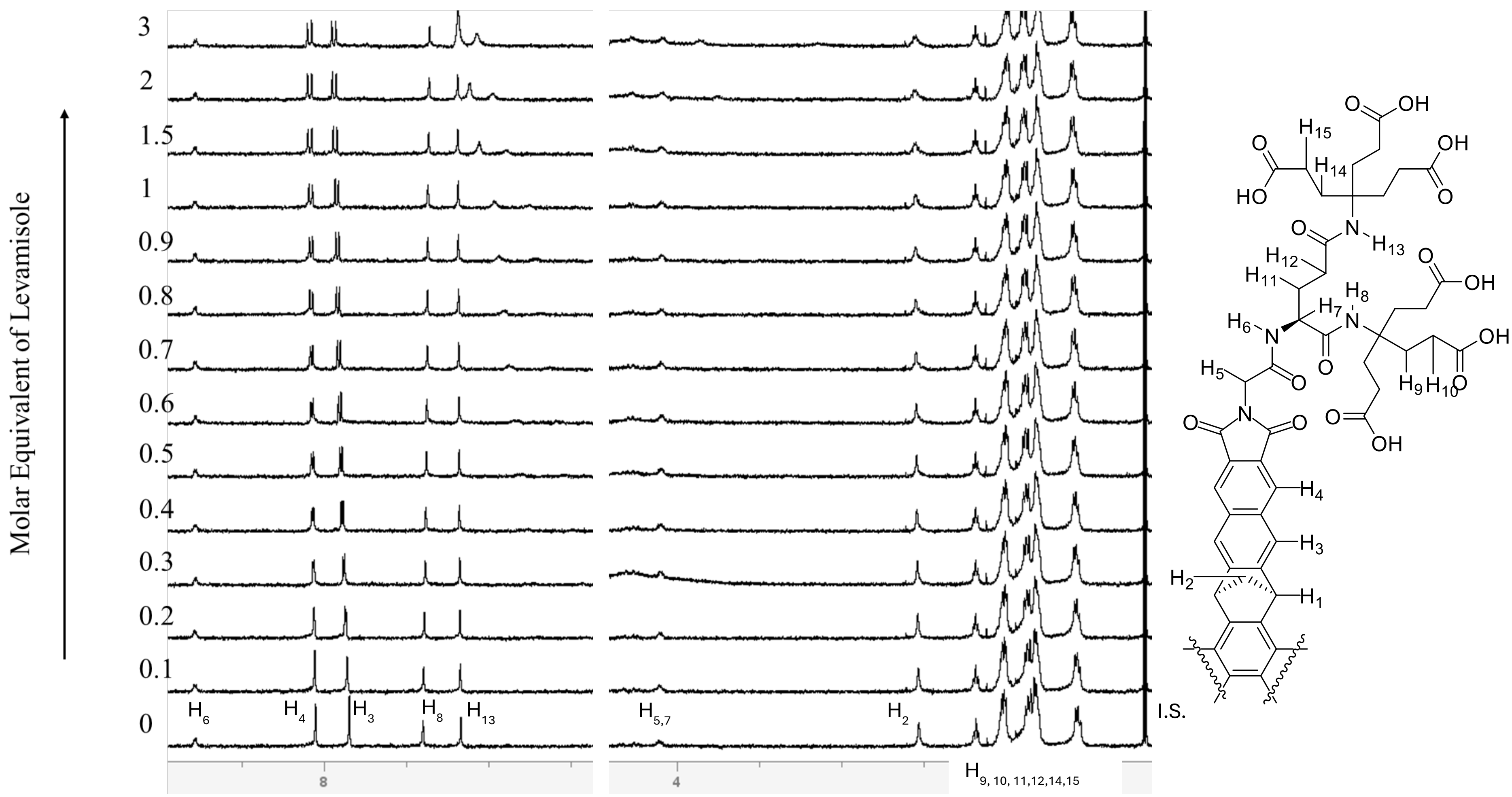

Figure S18. An incremental addition of 1.0 mM solution of LEV^+ to 50 μM basket $\mathbf{6}^{18-}$ (30 mM phosphate buffer at pH = 7.0, 90% H_2O:10% D_2O) was monitored with ^1H NMR spectroscopy (600 MHz, 298K; water suppression). Molar equivalents of LEV^+ are shown on the left. ; note that the signals from H_1 is within the water suppression region. Dimethyl sulfone was used as internal standard (I.S., 0.1 mM).

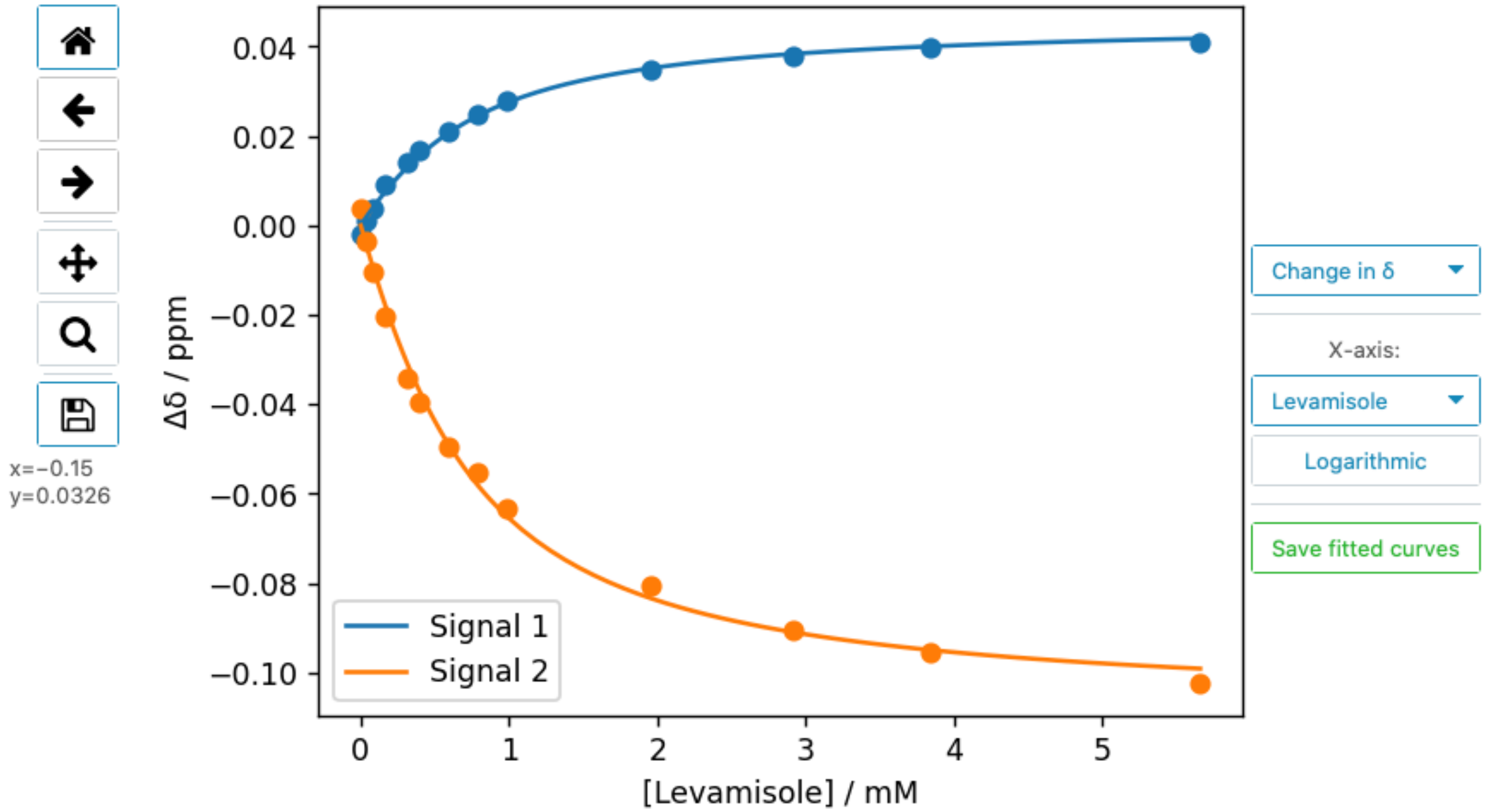

Figure S19. A change in ^{1}H NMR chemical shifts of signals at 7.54 and 3.92 ppm from $\mathbf{1}^{3-}$ (Figure S13) were fit to 1:1 model of complexation (Musketeer) giving (K_a = 2040 M^{-1}).

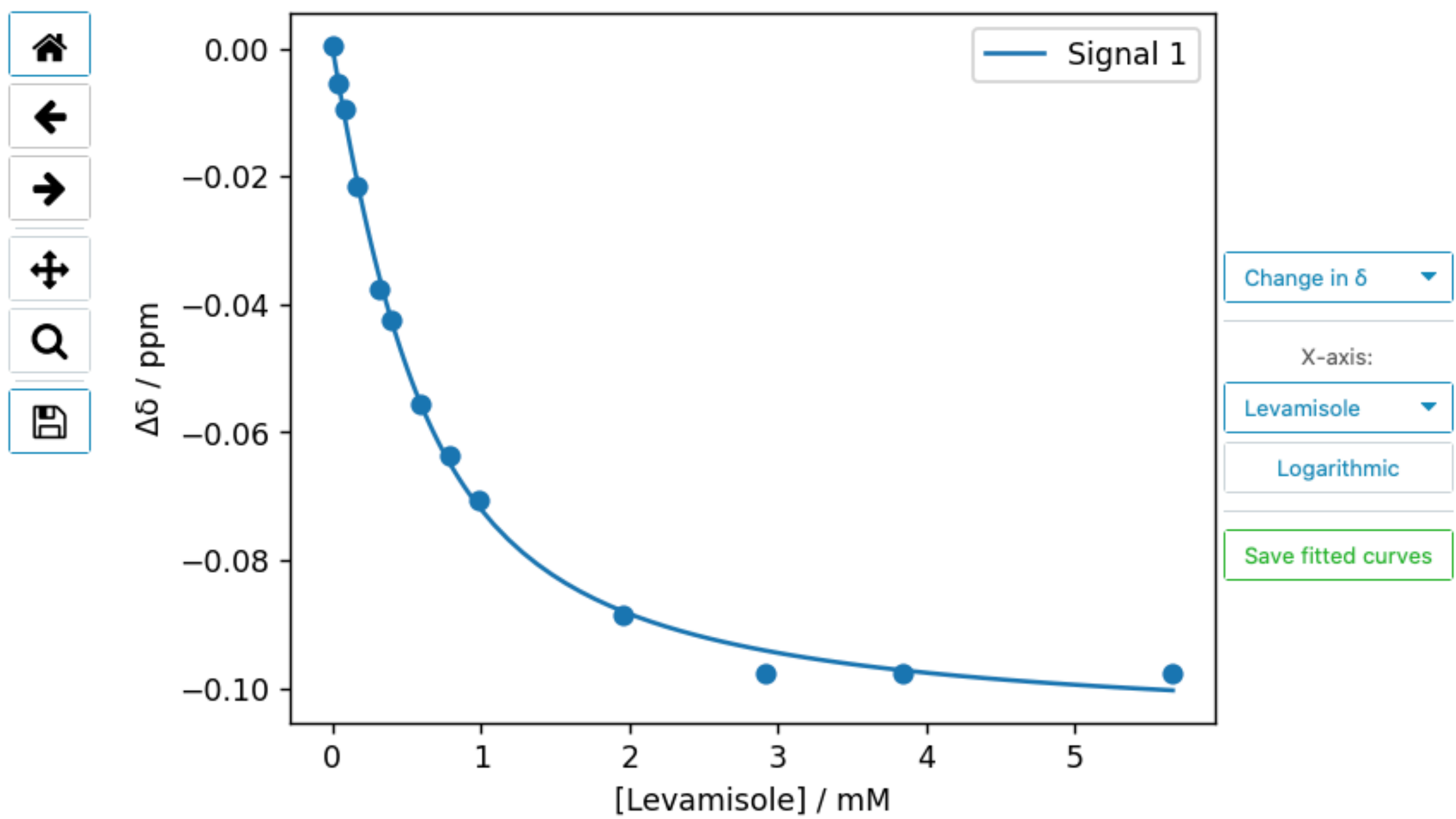

Figure S20. A change in ^{1}H NMR chemical shift of signal at 4.21 ppm from $\mathbf{2}^{6-}$ (Figure S14) was fit to 1:1 model of complexation (Musketeer) giving (K_a = 2870 M^{-1}).

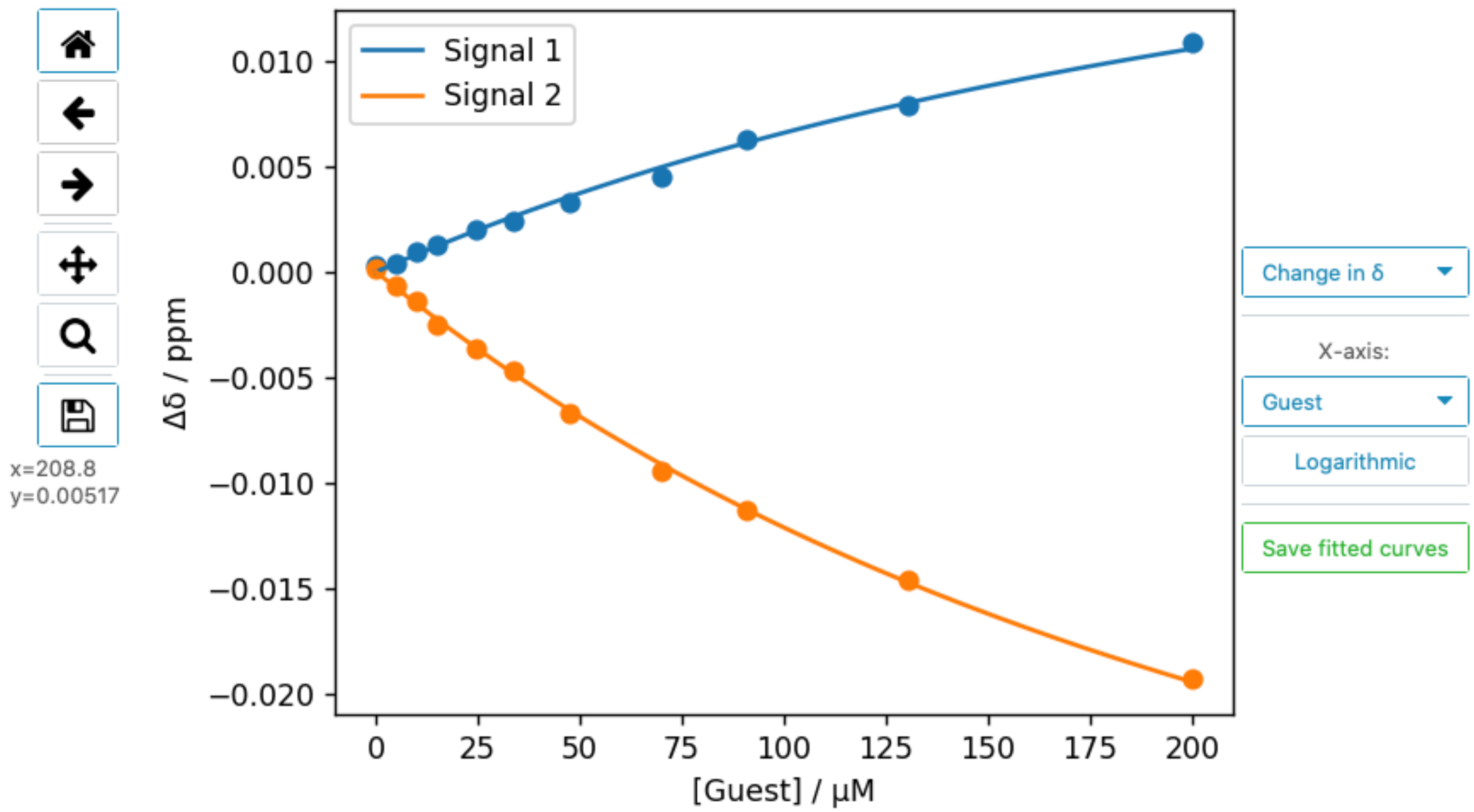

Figure S21. A change in ^{1}H NMR chemical shifts of signals at 7.62 and 8.49 ppm from $\mathbf{3}^{12-}$ (Figure S15) was fit to 1:1 model of complexation (Musketeer) giving (K_a = 4340 M^{-1}).

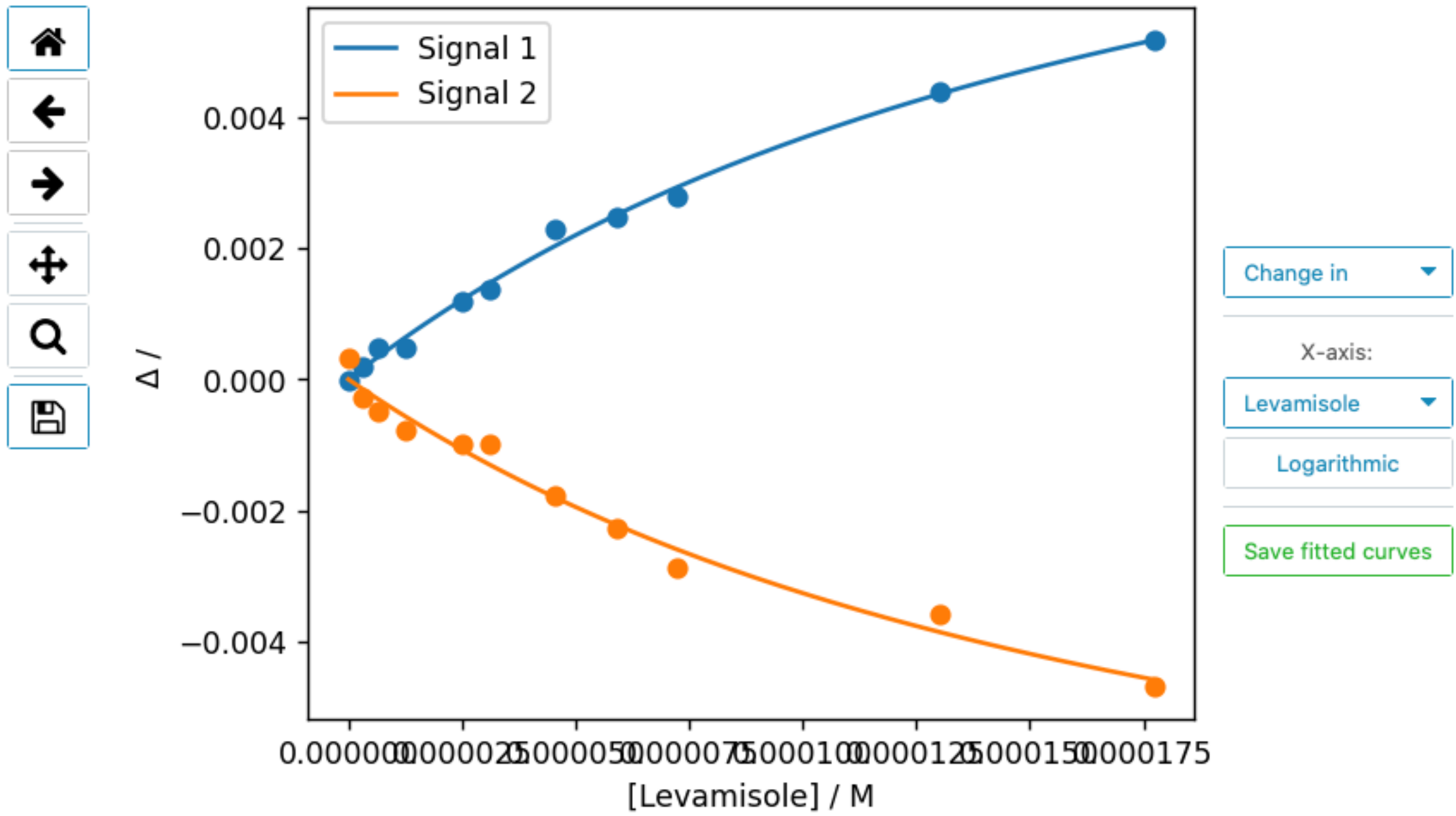

Figure S22. A change in ^{1}H NMR chemical shifts of signals at 7.67 and 7.41 ppm from $\mathbf{4}^{18-}$ (Figure S16) was fit to 1:1 model of complexation (Musketeer) giving (K_a = 6370 M^{-1}).

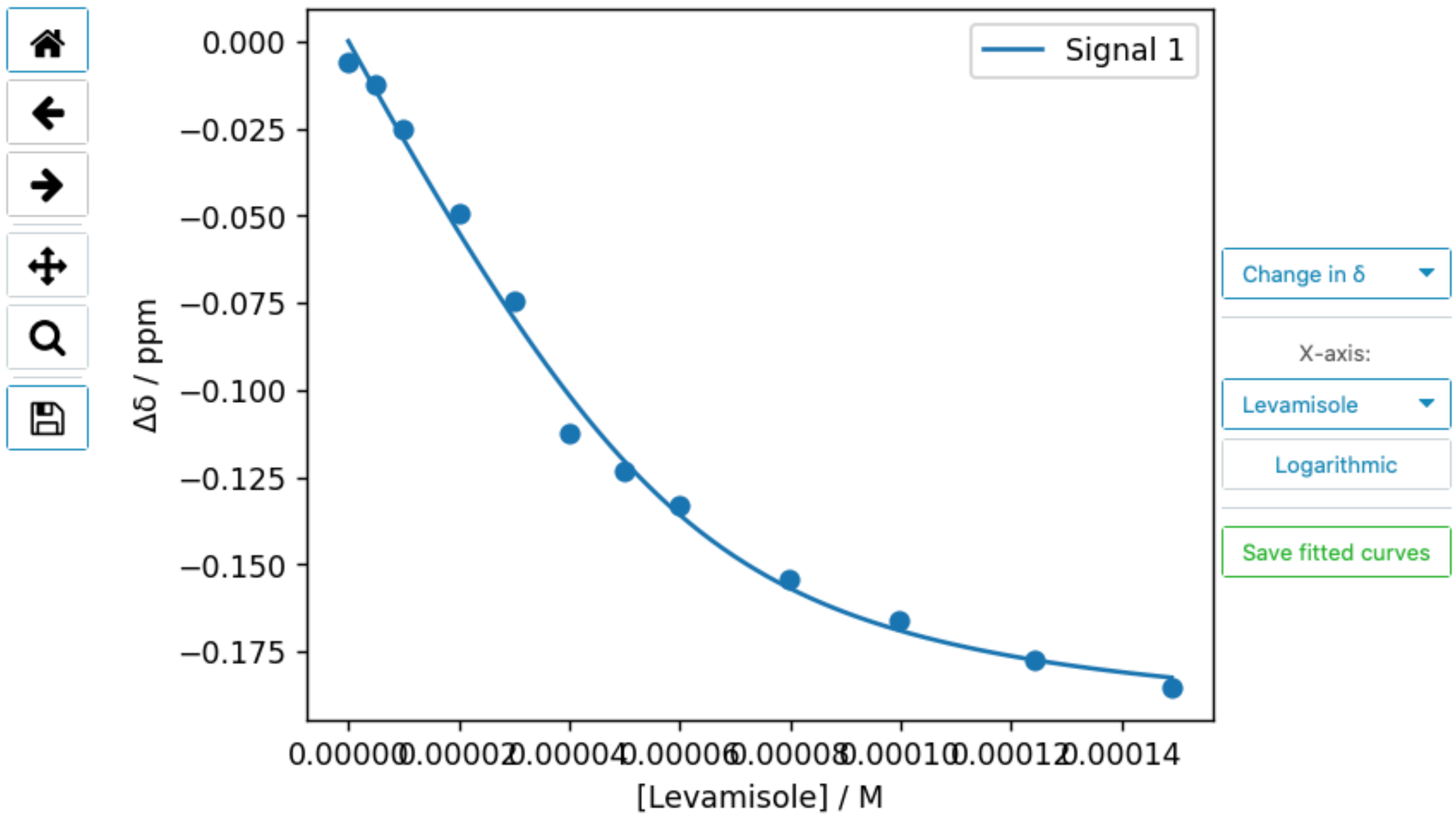

Figure S23. A change in ^{1}H NMR chemical shift of signal at 8.58 ppm from $\mathbf{5}^{12-}$ (Figure S17) was fit to 1:1 model of complexation (Musketeer) giving (K_a = 114000 M^{-1}).

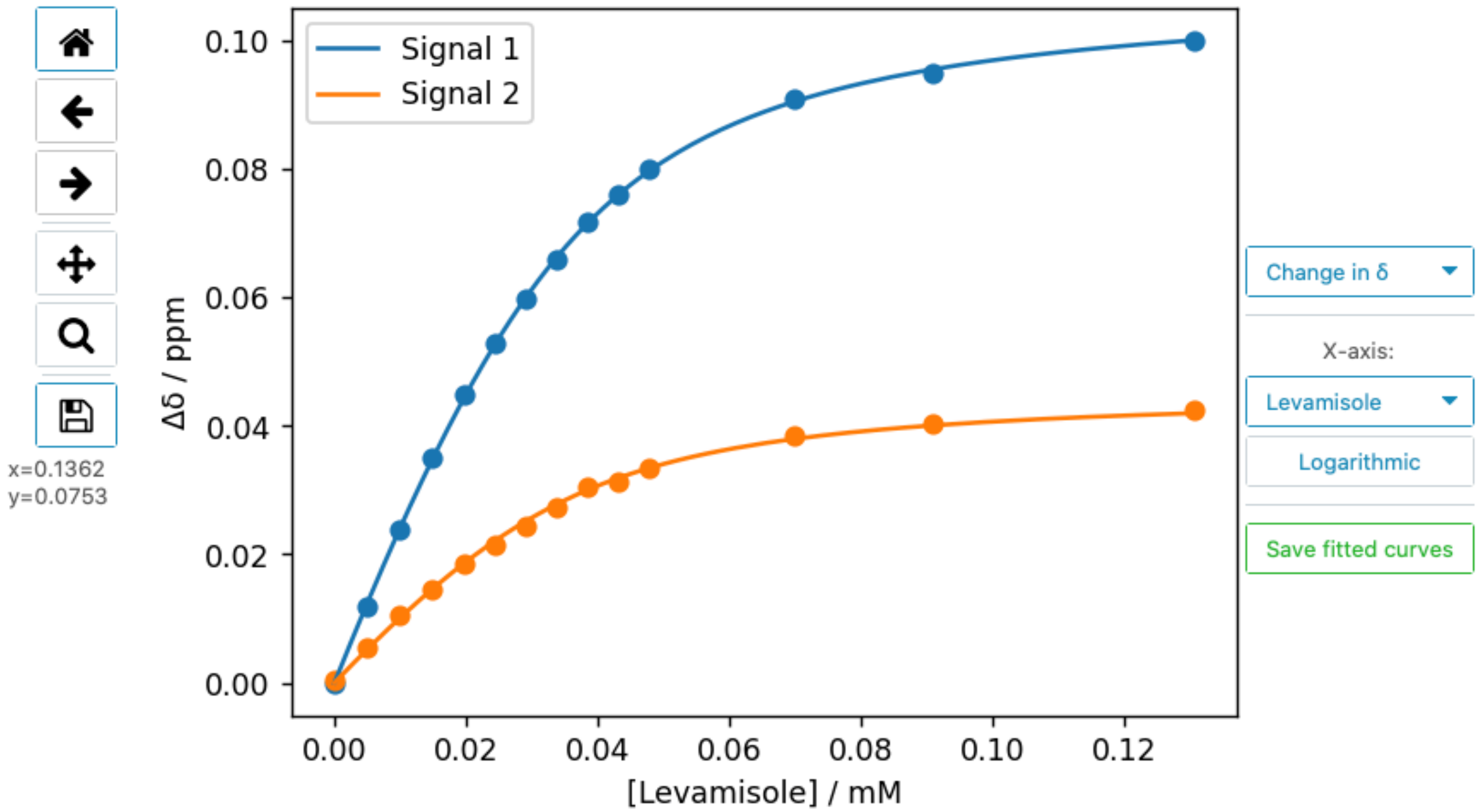

Figure S24. A change in ^{1}H NMR chemical shift of signal at 7.88 and 8.05 ppm from **6**$^{18-}$ (Figure S18) was fit to 1:1 model of complexation (Musketeer) giving (K_a = 125000 M^{-1}).

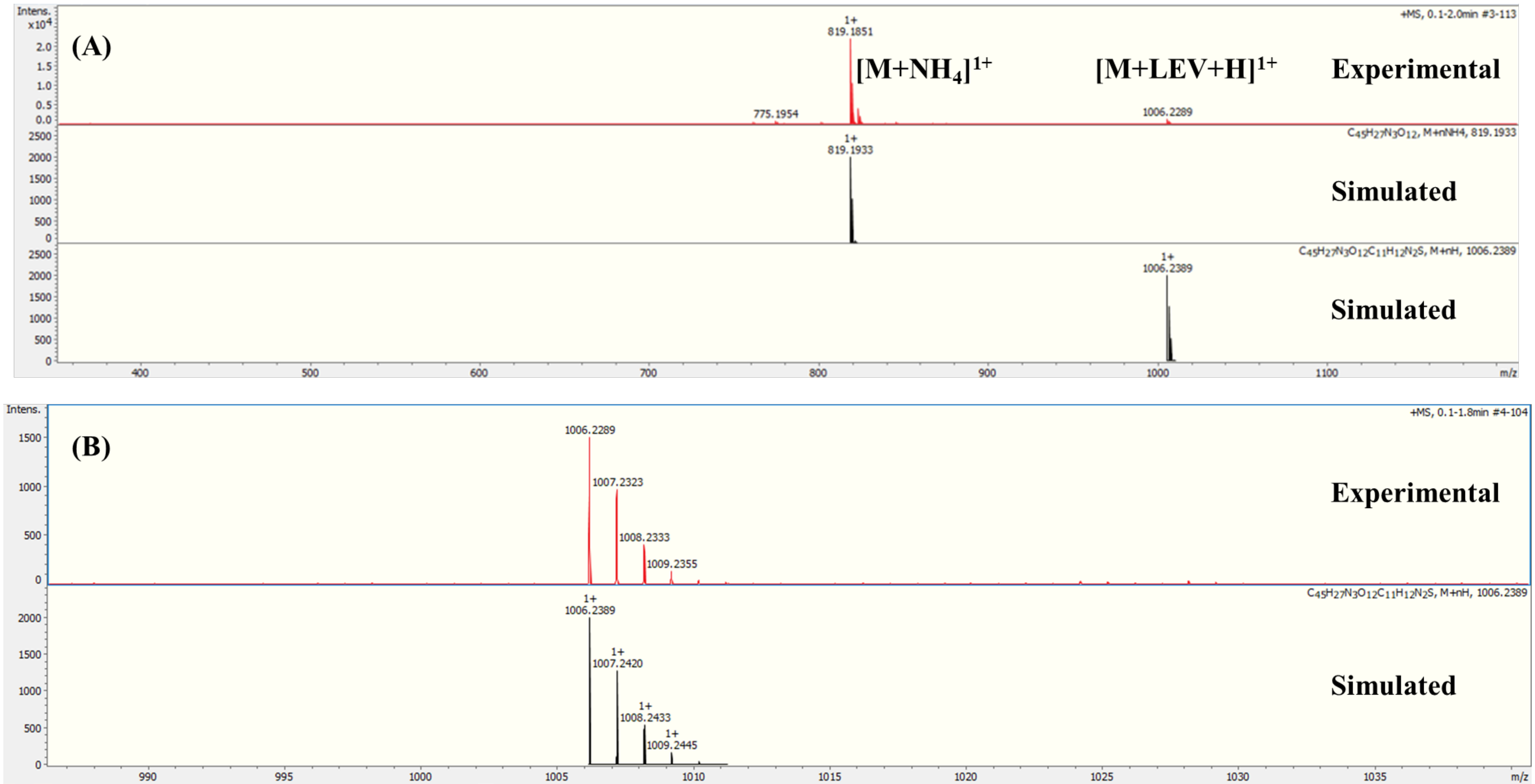

Figure S25. (A) Experimental and simulated electron-spray ionization (ESI) data for 50 μM solution of molecular basket $\mathbf{1}^{3-}$ and levamisole HCl (LEV^+) (i.e., equimolar ratio) in 25 mM ammonium bicarbonate buffer at pH = 6.8. (B) Experimental and simulated isotope distribution pattern for an ion with the mass corresponding to $[LEV\subset\mathbf{1}]^+$.

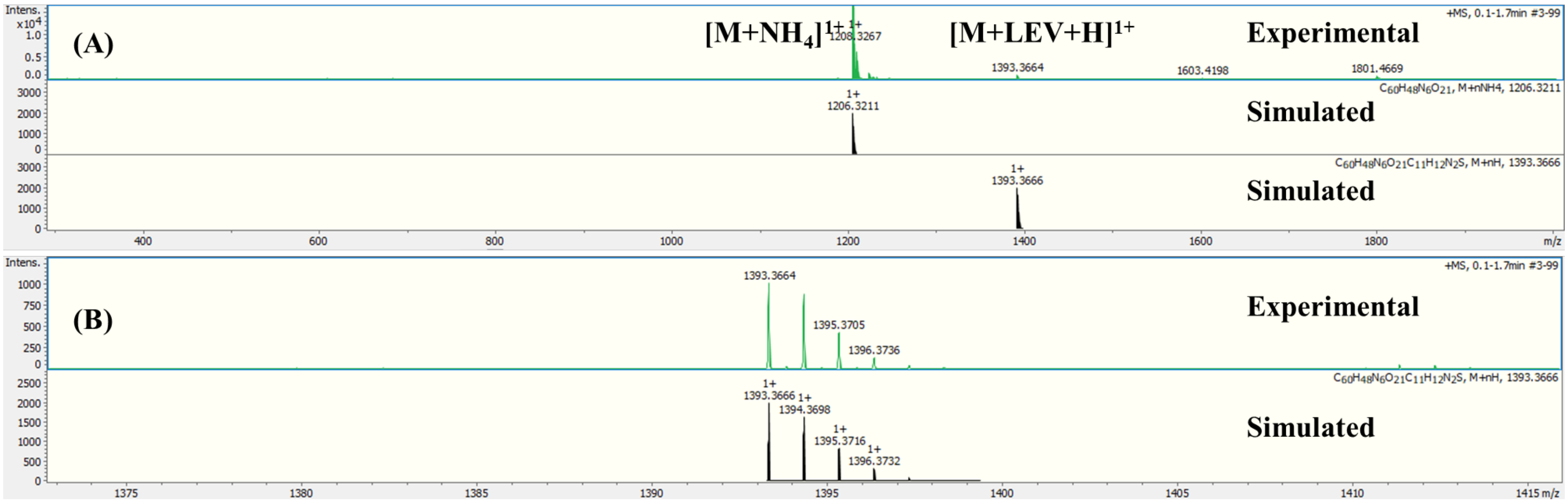

Figure S26. (A) Experimental and simulated electron-spray ionization (ESI) data for 50 µM solution of molecular basket $\mathbf{2}^{6-}$ and levamisole HCl (LEV^{+}) (i.e., equimolar ratio) in 25 mM ammonium bicarbonate buffer at pH = 6.8. (B) Experimental and simulated isotope distribution pattern from an ion with the mass corresponding to $[LEV\subset\mathbf{2}]^{+}$.

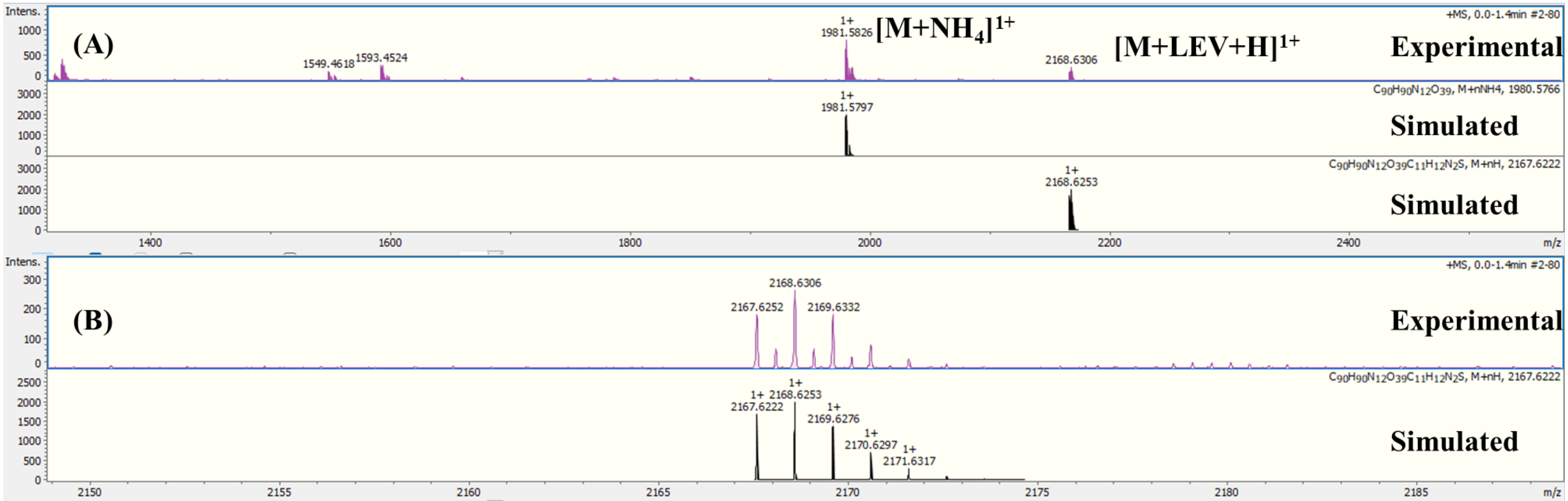

Figure S27. (A) Experimental and simulated electron-spray ionization (ESI) data for 50 µM solution of molecular basket $\mathbf{3}^{12-}$ and levamisole HCl (LEV^+) (i.e., equimolar ratio) in 25 mM ammonium bicarbonate buffer at pH = 6.8. (B) Experimental and simulated isotope distribution pattern from an ion with the mass corresponding to $[LEV\subset\mathbf{3}]^+$.

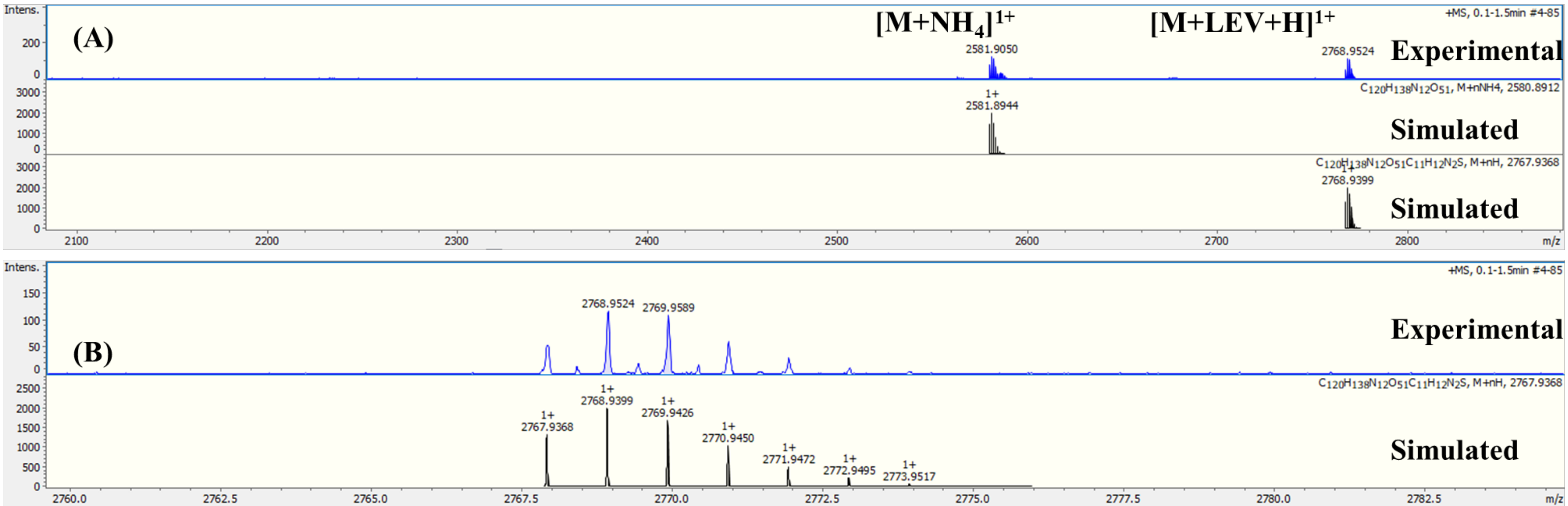

Figure S28. (A) Experimental and simulated electron-spray ionization (ESI) data for 50 µM solution of molecular basket $\mathbf{4}^{18-}$ and levamisole HCl (LEV^{+}) (i.e., equimolar ratio) in 25 mM ammonium bicarbonate buffer at pH = 6.8. (B) Experimental and simulated isotope distribution pattern from an ion with the mass corresponding to $[LEV \subset \mathbf{4}]^{+}$.

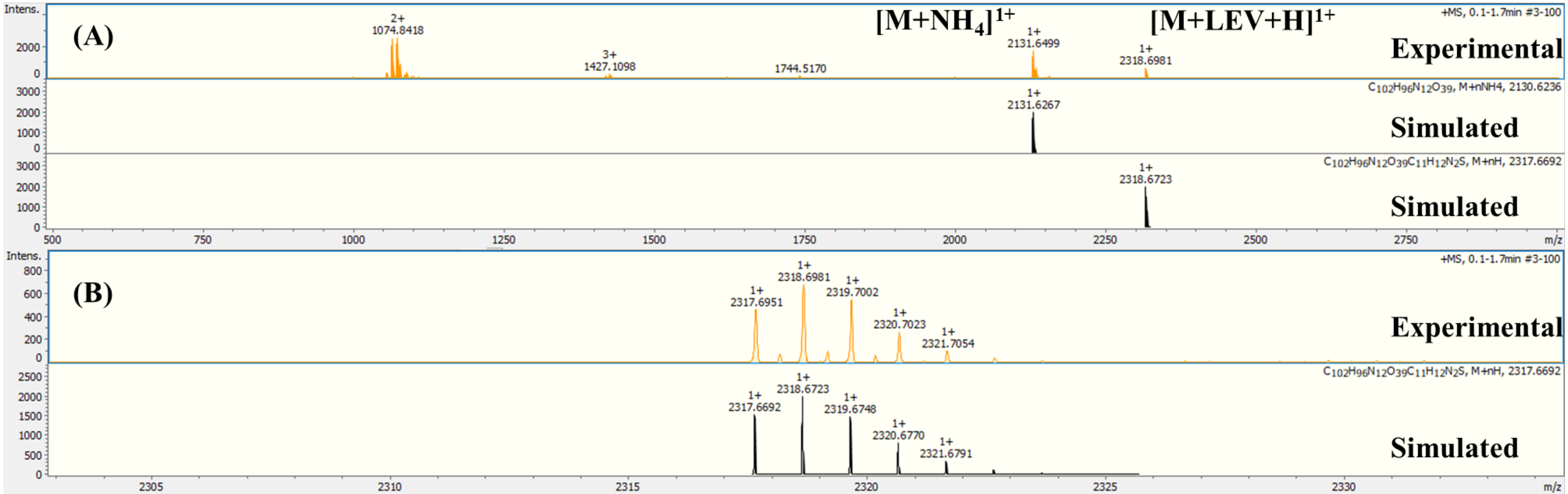

Figure S29. (A) Experimental and simulated electron-spray ionization (ESI) data for 50 µM solution of molecular basket $\mathbf{5}^{12-}$ and levamisole HCl (LEV^{+}) (i.e., equimolar ratio) in 25 mM ammonium bicarbonate buffer at pH = 6.8. (B) Experimental and simulated isotope distribution pattern from an ion with the mass corresponding to $[LEV\subset\mathbf{5}]^{+}$.

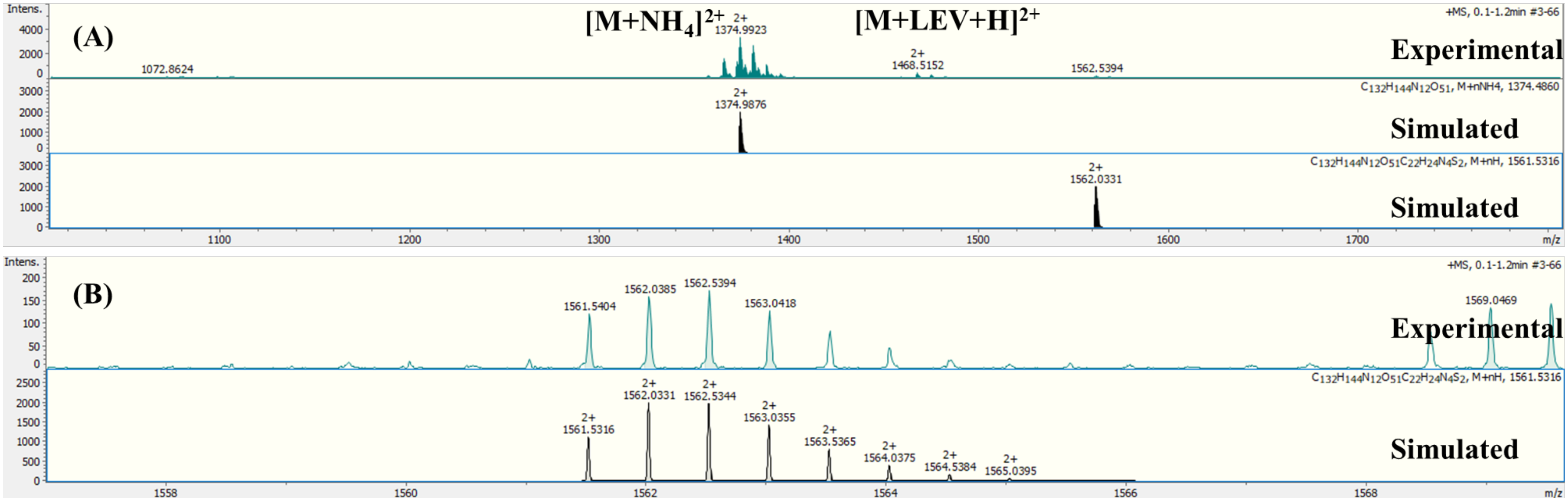

Figure S30. (A) Experimental and simulated electron-spray ionization (ESI) data for 50 µM solution of molecular basket $\mathbf{6}^{18-}$ and levamisole HCl (LEV^{+}) (i.e., equimolar ratio) in 25mM ammonium bicarbonate buffer at pH = 6.8. (B) Experimental and simulated isotope distribution pattern from an ion with the mass corresponding to $[LEV\subset\mathbf{6}]^{+}$.

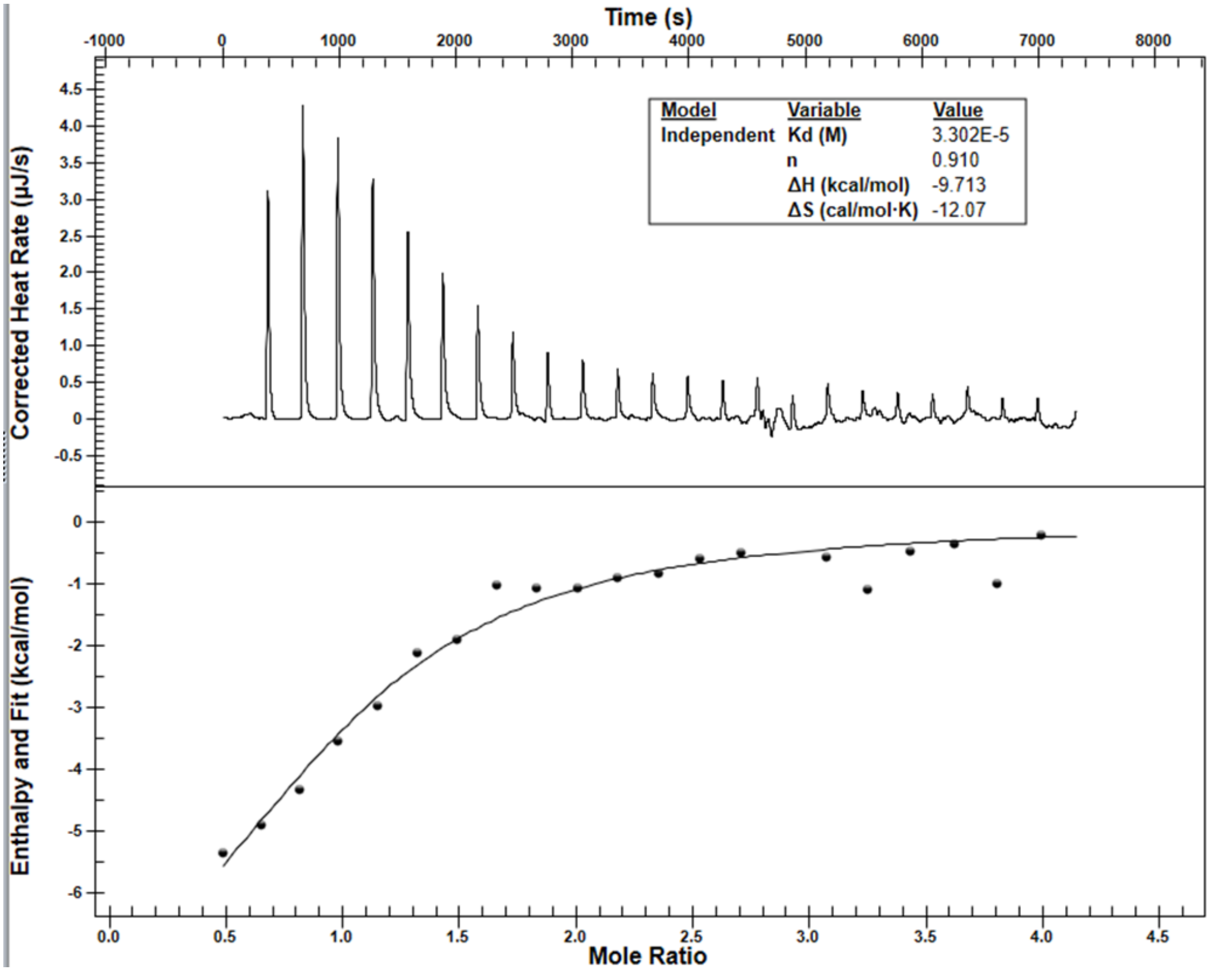

Figure S31. An incremental addition of 2.4 mM solution of LEV^+ from the syringe (50 μL per injection) to 300 μL of 0.1 mM of molecular basket $\mathbf{6}^{18-}$ (30 mM phosphate buffer at pH = 7.0 ± 0.1) was monitored with isothermal titration calorimetry (ITC). The corrected heat rate is shown on the top, while the standard enthalpy as a function of molar equivalents of LEV^+ along with the fitted curve (1:1 model of binding; solid line) is shown at the bottom.

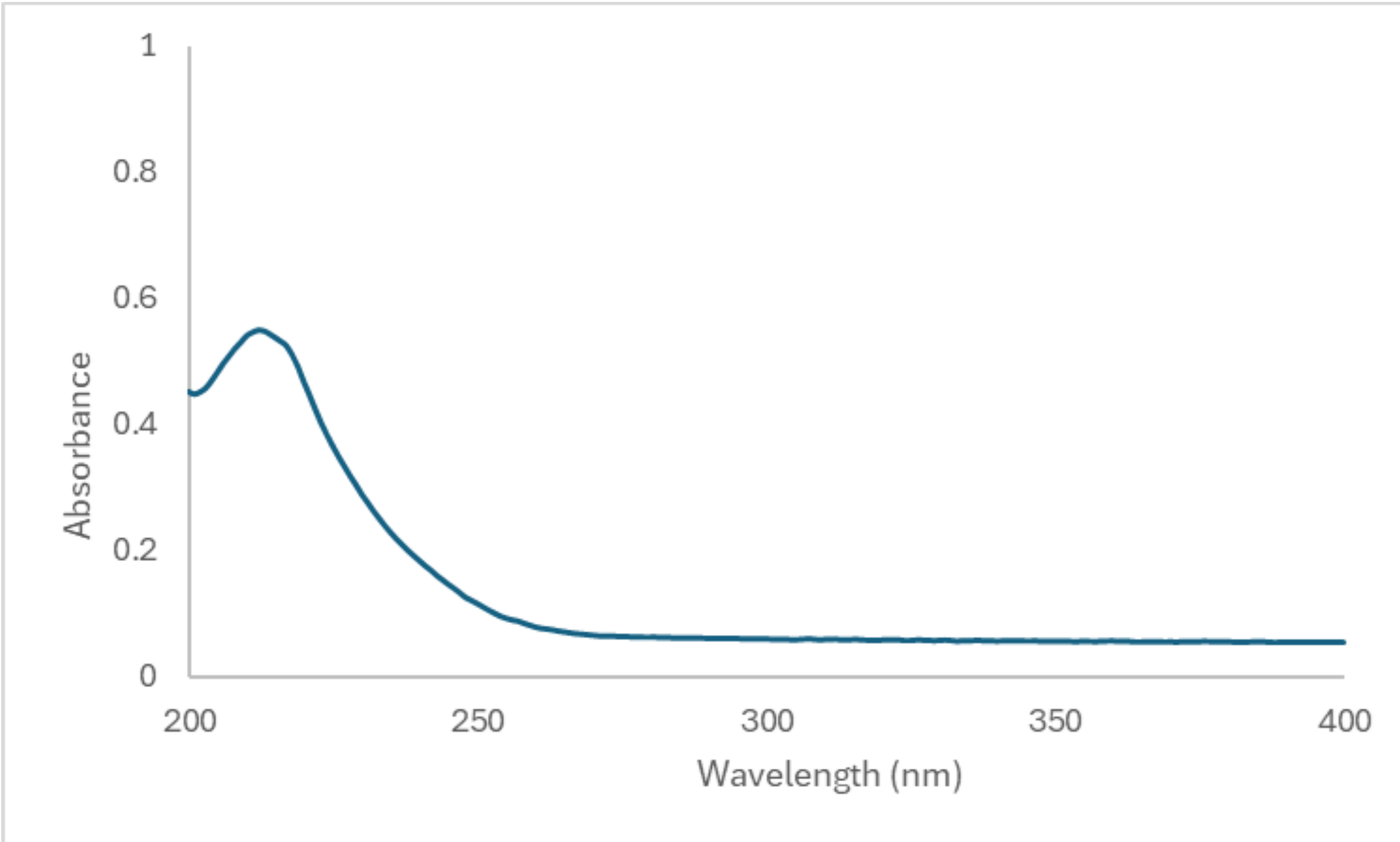

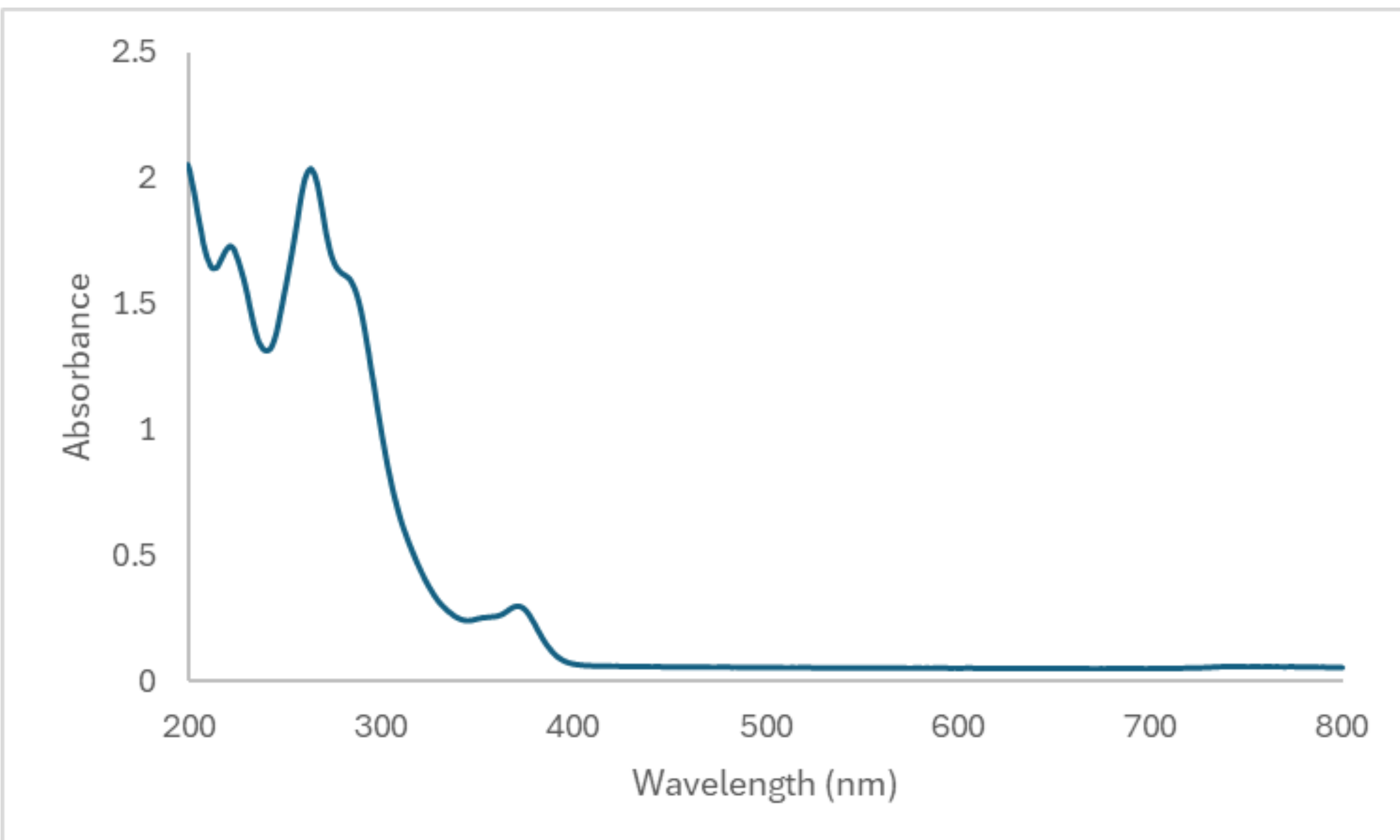

Figure S32. (Top) UV-Vis spectrum of 25 μM LEV^+ in 30 mM phosphate buffer pH 7.0 ± 0.1. (Bottom) UV-Vis spectrum of 25 μM basket $\mathbf{6}^{18-}$ in 30 mM phosphate buffer pH 7.0 ± 0.1.

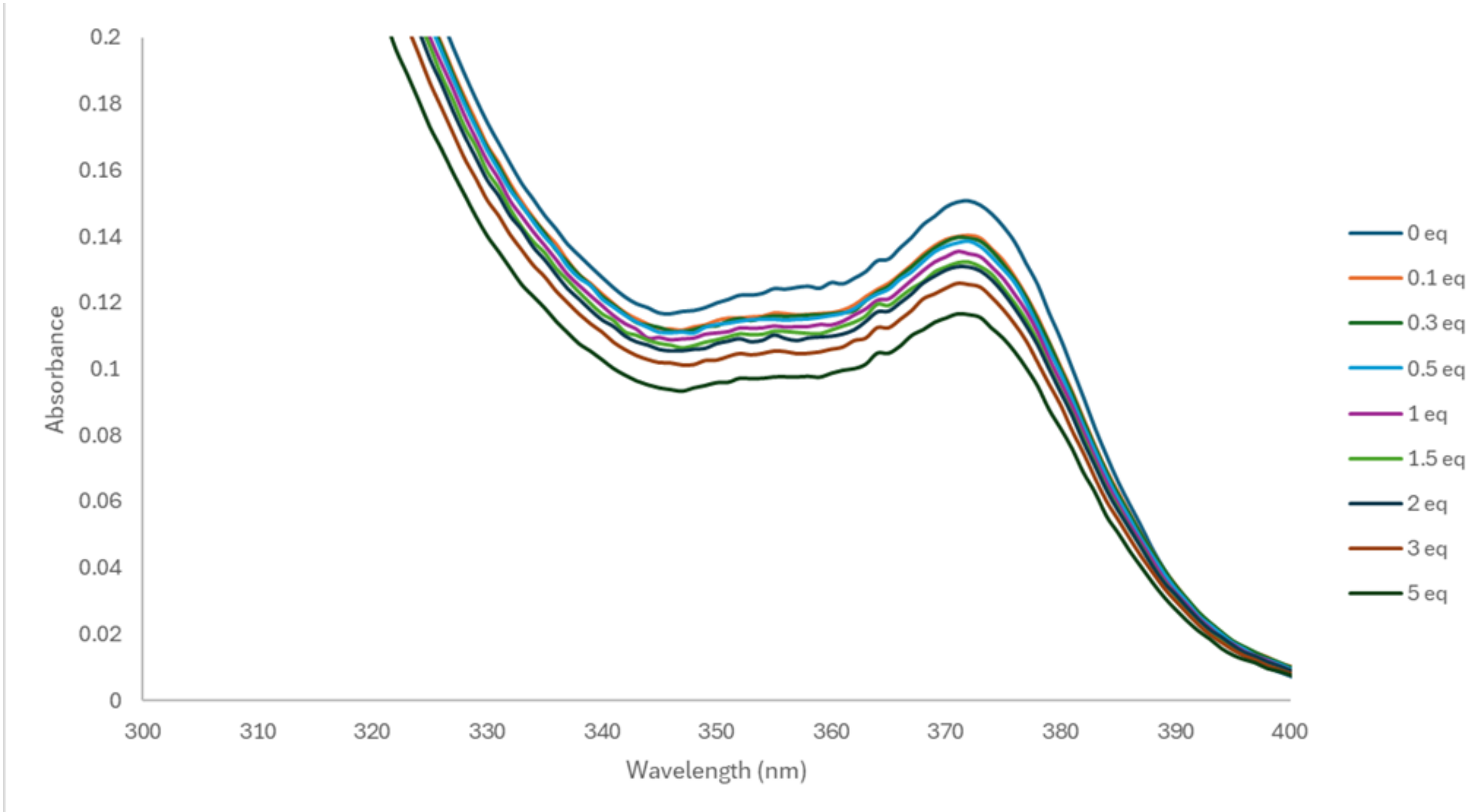

Figure S33. UV-Vis spectrum of basket **6**$^{18-}$ (50 µM in 30 mM phosphate buffer at pH 7.0 ± 0.1) recorded upon an incremental addition of 1.0 mM solution of LEV^{+} (30 mM phosphate buffer at pH = 7.0).

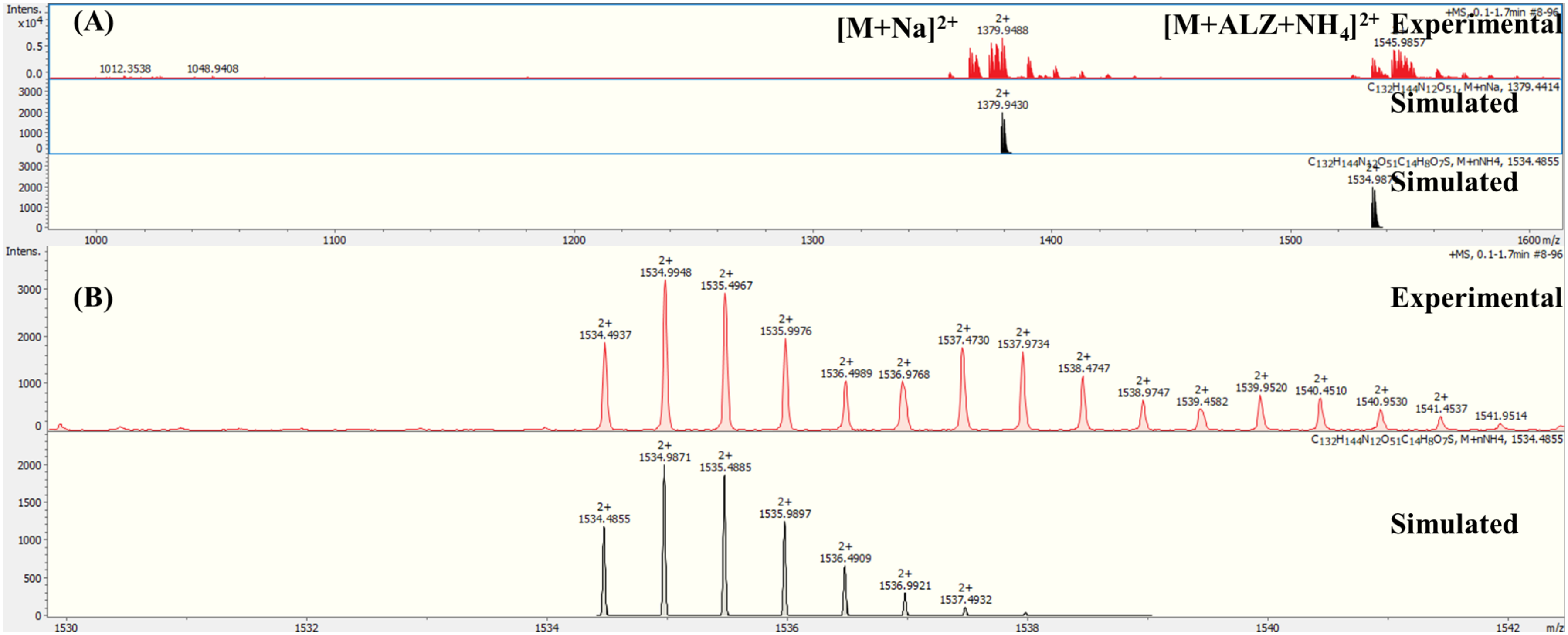

Figure S34. (A) Experimental and simulated electron-spray ionization (ESI) data for 50 µM solution of molecular basket **6**$^{18-}$ and Alizarin Red S (ALZ$^-$) (i.e., equimolar ratio) in 25 mM ammonium bicarbonate buffer at pH = 6.8. B: Experimental and simulated isotope distribution pattern from an ion with the mass corresponding to [ALZ⊂**6**]$^{2+}$.

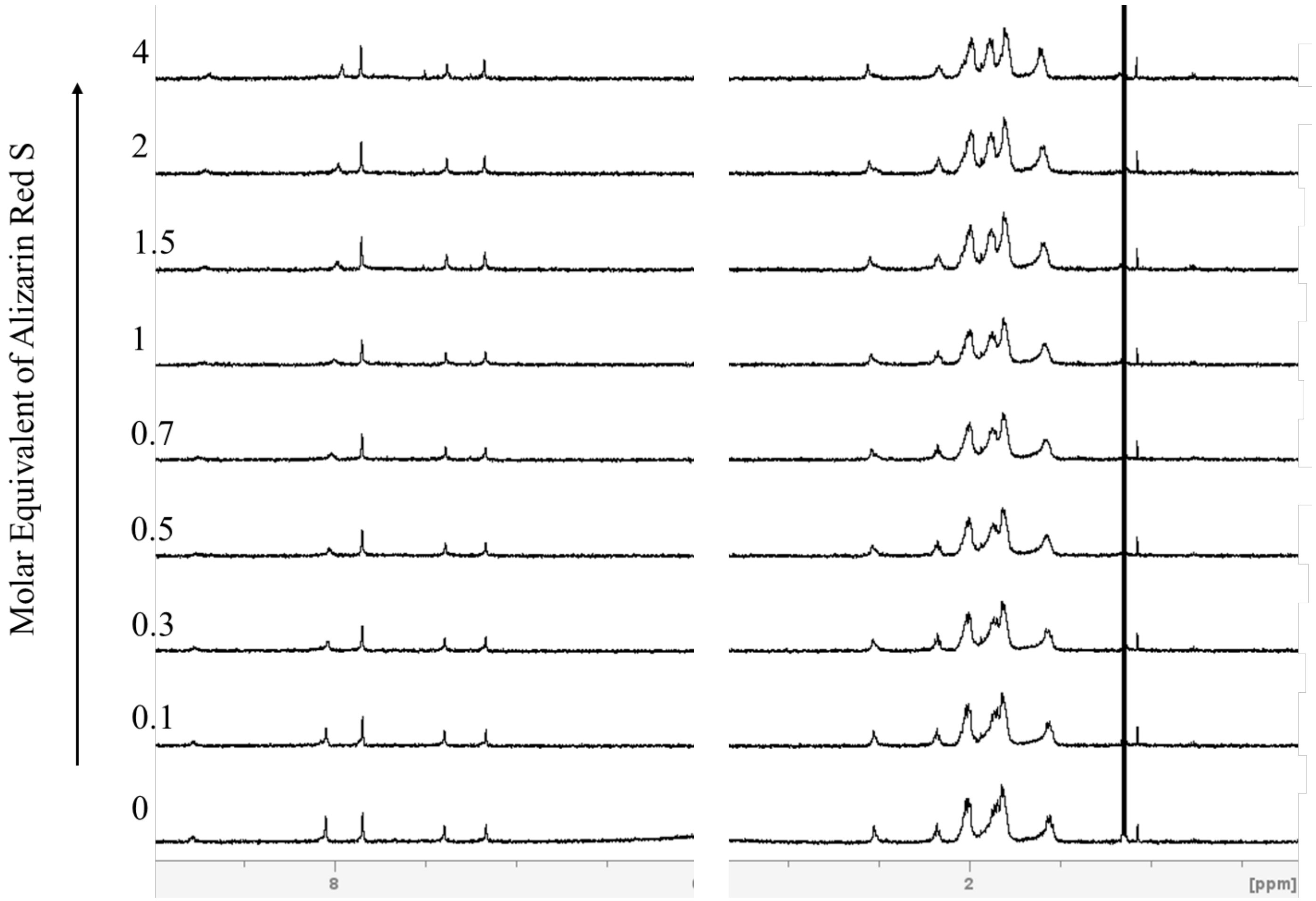

Figure S35. An incremental addition of 1.0 mM solution of ALZ^- to 100 μM basket $\mathbf{6}^{18-}$ in 30 mM phosphate buffer at pH = 7.0 (90% H_2O: 10% D_2O) was monitored with ^1H NMR spectroscopy (600 MHz, 298K). The number of molar equivalents of ALZ^- are shown on the left. Dimethyl malonic acid was used as internal standard (0.1 mM).

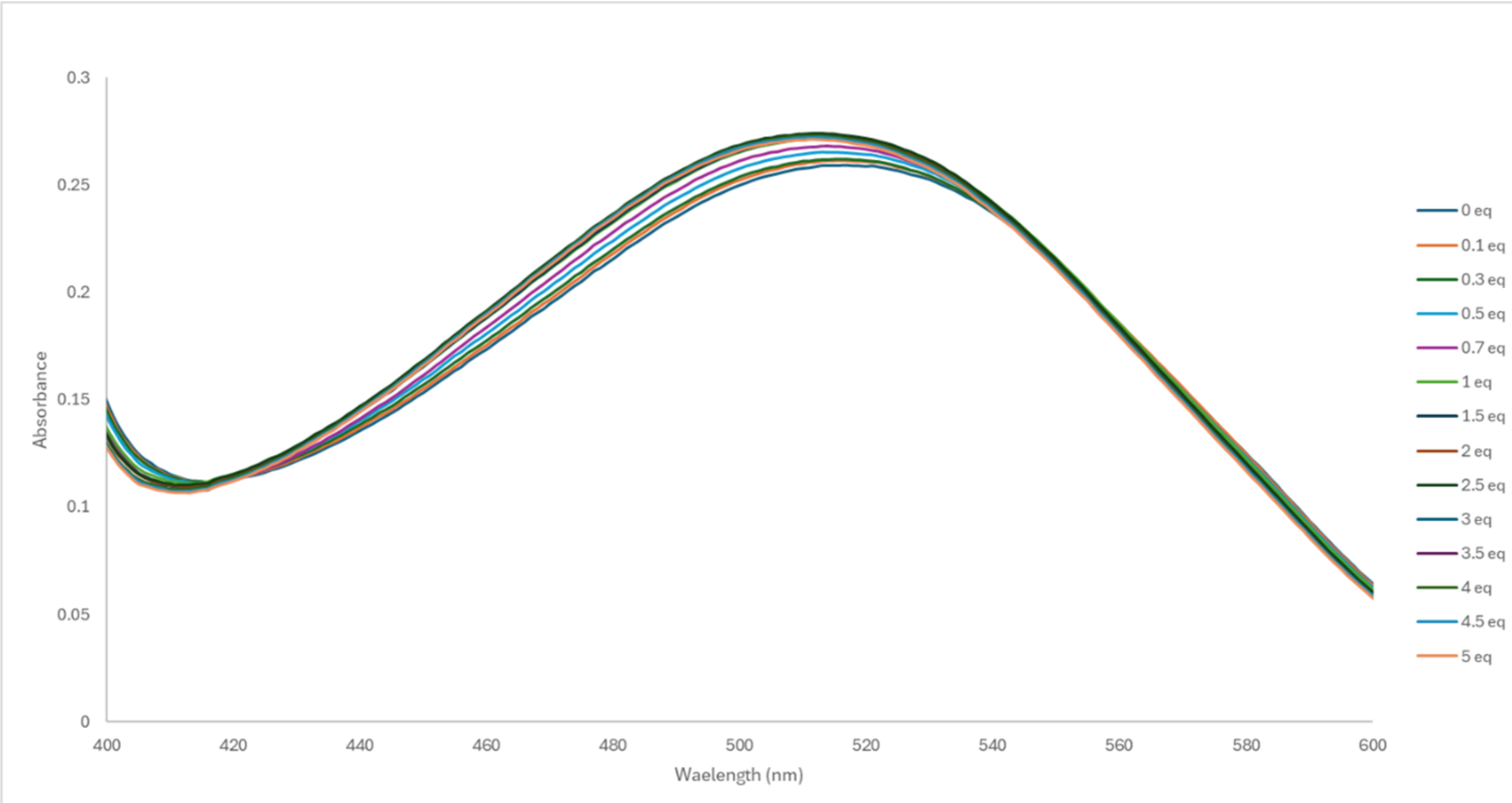

Figure S36. An incremental addition of 20 mM LEV^{+} to a solution of 100 μM basket $\mathbf{6}^{18-}$ and 50 μM ALZ^{-} in 30 mM phosphate buffer at pH = 7.0 was monitored with UV-Vis spectroscopy.

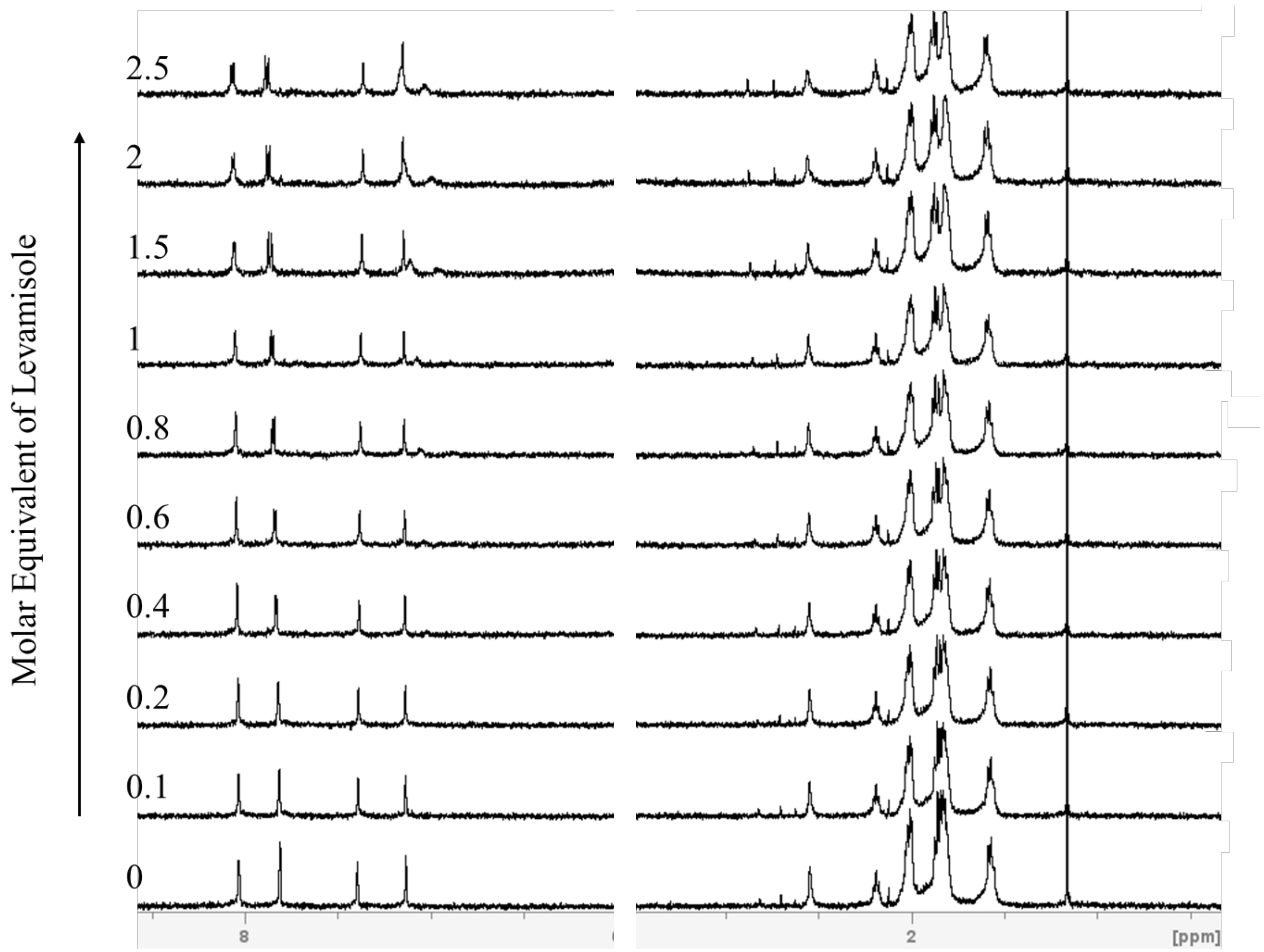

Figure S37. An incremental addition of 1 mM solution of LEV^+ to 0.1 mM basket $\mathbf{6}^{18-}$ in 100 mM borate buffer at pH = 8.0 (90% H_2O: 10% D_2O) was monitored with ^1H NMR spectroscopy (600 MHz, 298K). The number of molar equivalents of LEV^+ are shown on the left. Dimethyl malonic acid was used as internal standard (0.1 mM).

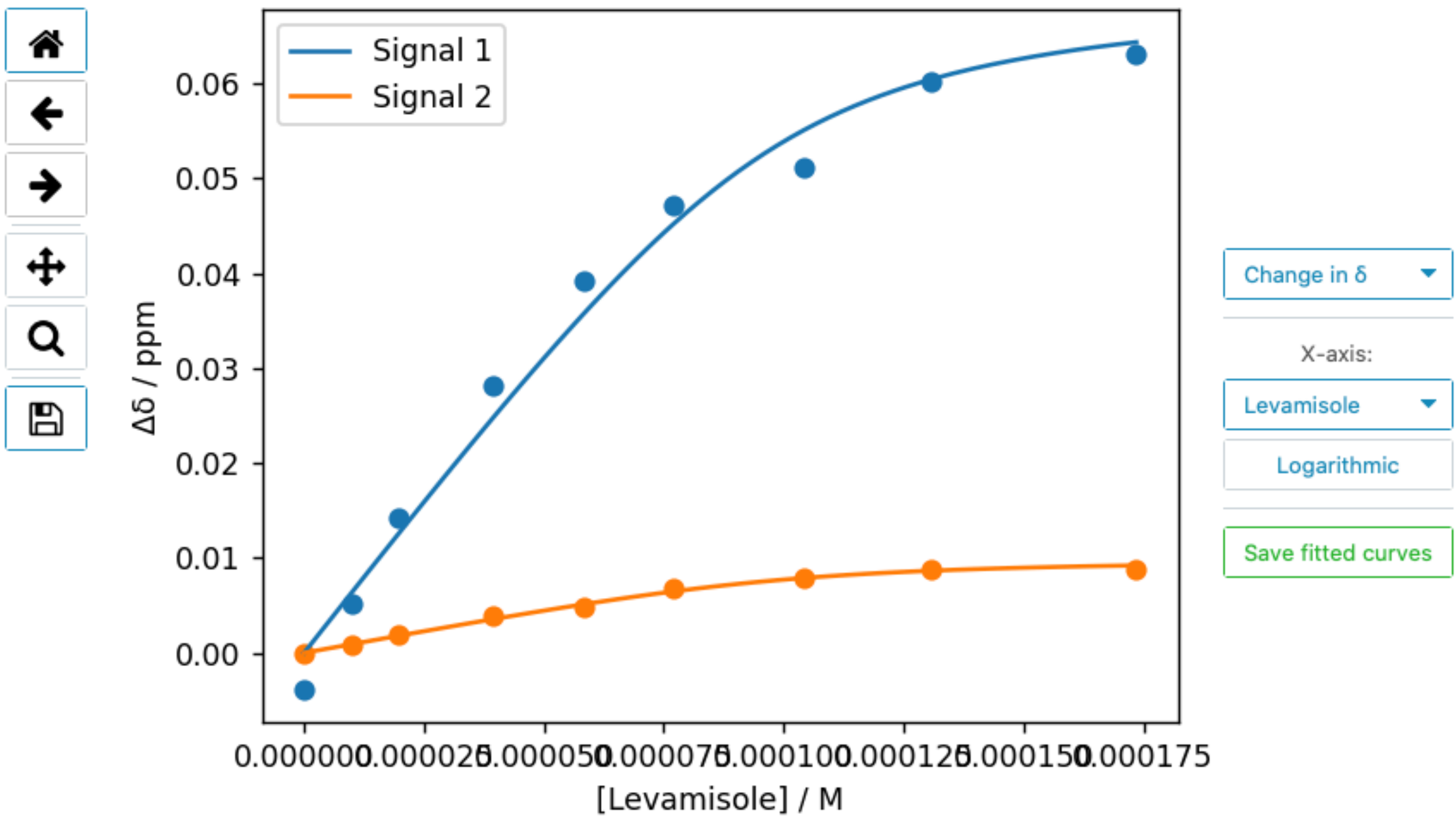

Figure S38. A change in ^{1}H NMR chemical shift of signal at 7.88 and 8.10 ppm from **6**$^{18-}$ (Figure S37) were fit to 1:1 model of complexation (Musketeer) giving (K_a = 129000 M^{-1}).

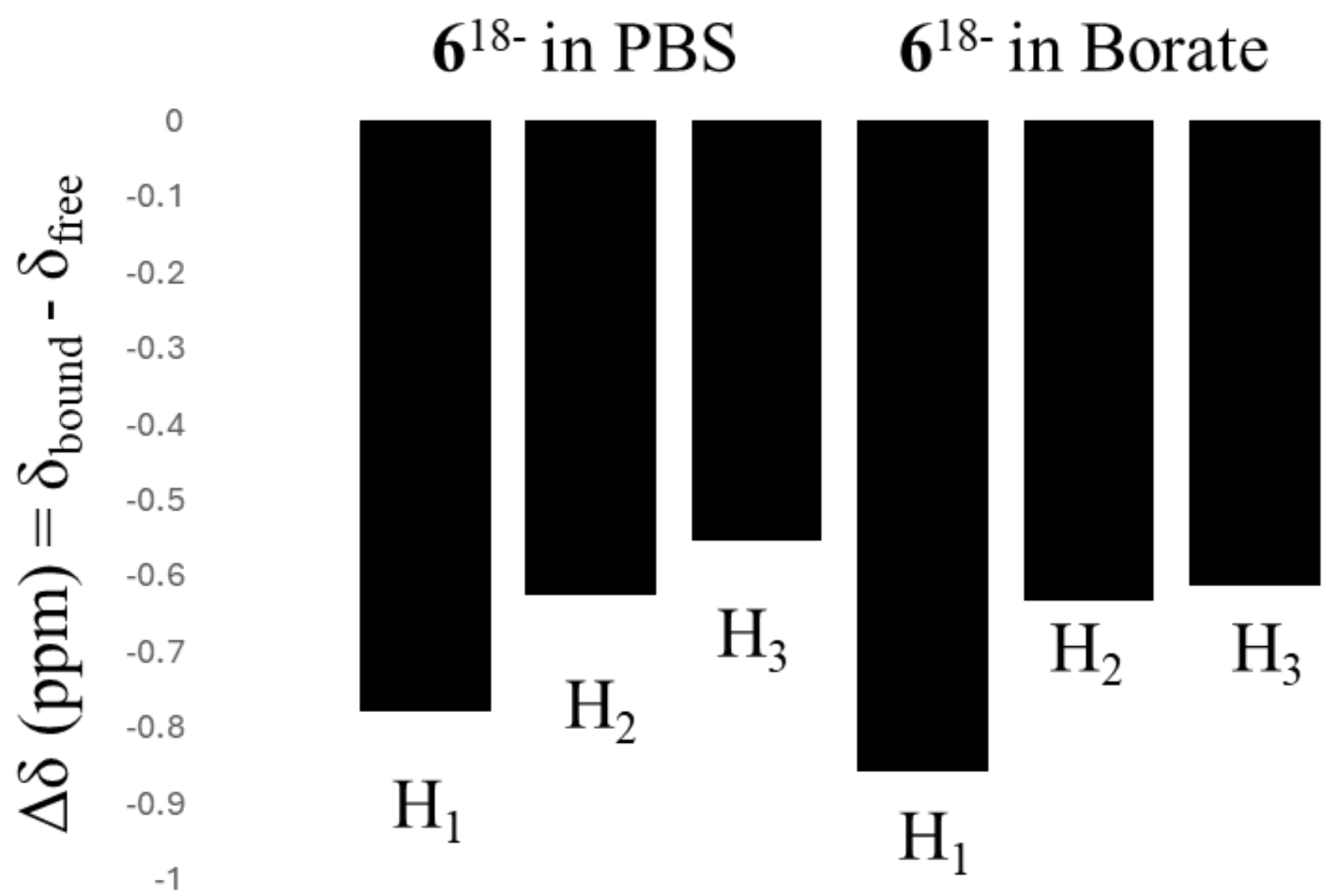

Figure S39. The observed change in ^{1}H NMR chemical shifts ($\Delta\delta = \delta_{bound} - \delta_{free}$) of protons H_1, H_2, and H_3 from LEV^+ after complexing basket **6**$^{18-}$ (left) in 30 mM phosphate buffer at pH = 7.0 and (right) 100 mM borate buffer at pH = 8.1; for further clarification, see Figure 3B.

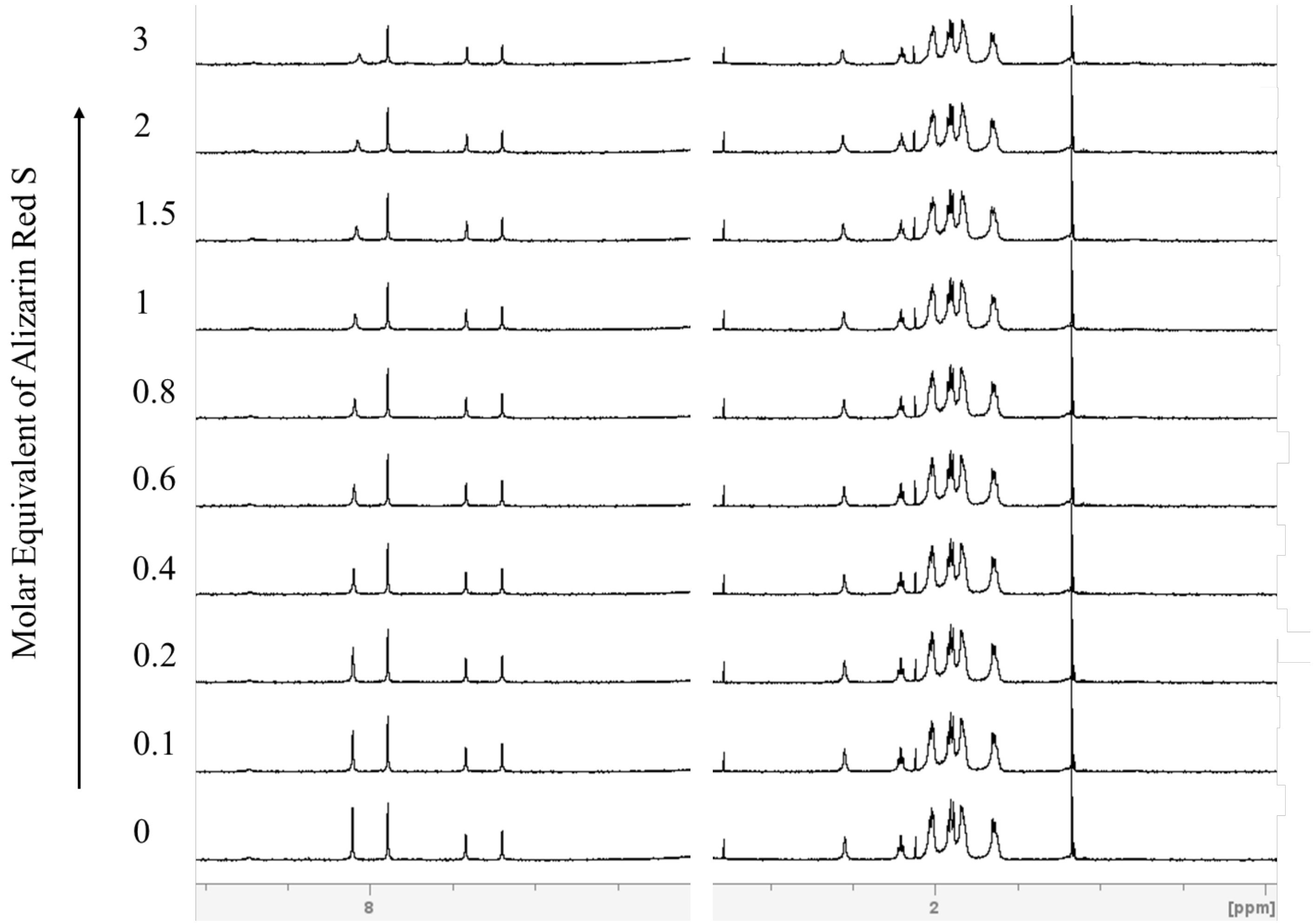

Figure S40. An incremental addition of 1.0 mM solution of ALZ^{-} to 0.05 mM basket $\mathbf{6}^{18-}$ in 100 mM borate buffer at pH = 8.1 (90% H_2O: 10% D_2O) was monitored with ^{1}H NMR spectroscopy (600 MHz, 298K). The number of molar equivalents of ALZ^{-} are shown on the left. Dimethyl malonic acid was used as internal standard (0.1 mM).

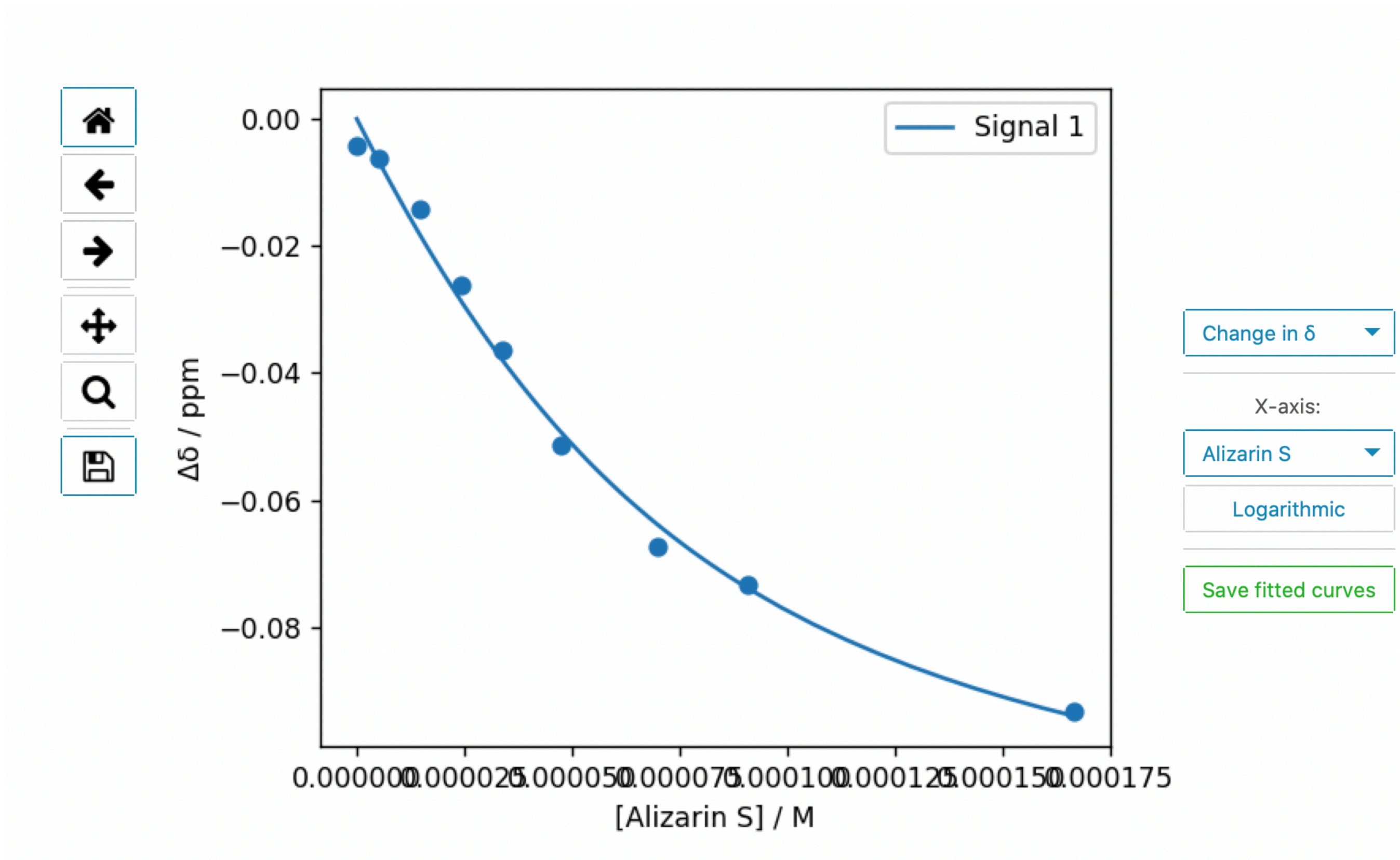

Figure S41. A change in ^{1}H NMR chemical shift of signal at 8.05 ppm from **6**$^{18-}$ (Figure S40) was fit to 1:1 model of complexation (Musketeer) giving (K_a = 22600 M^{-1}).

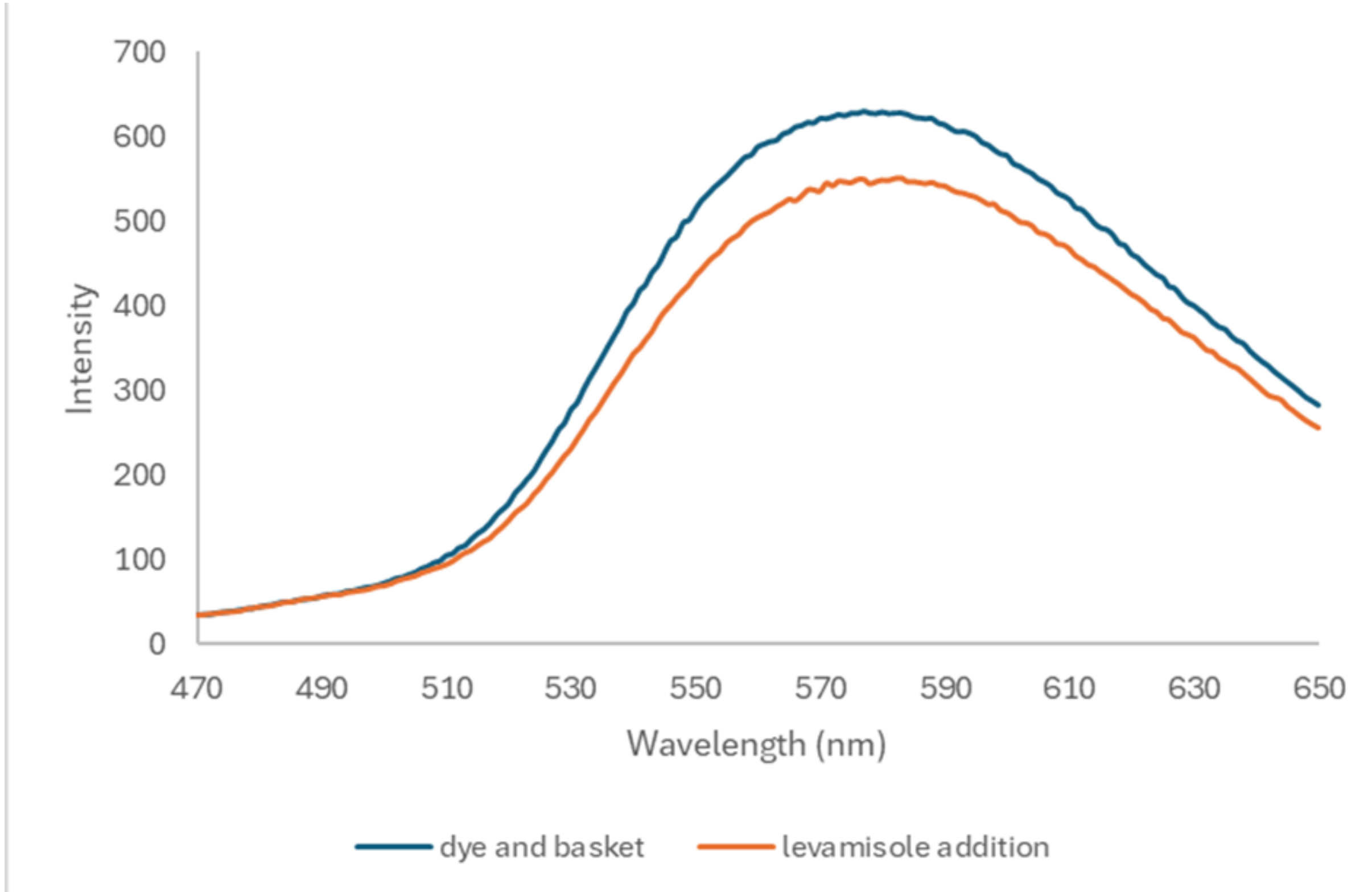

Figure S42. To a solution (2.0 mL) of 80 µM alizarin red s and 160 µM basket **6**$^{18-}$ in 100 mM borate buffer at pH = 8.1 (blue line), we added of 20 µL of 8.39 mM standard solution of LEV^+ (orange line). The fluorescence intensity of ALZ^- at 581 nm drops from 628 to 548 (blue to orange lines). The initial intensity of 628 was normalized with regard to the calibration curve to 685 (Figure 5B), therefore changing the reading value upon the addition of LEV^+ from 548 to 605. From the calibration curve in Figure 5B (main text), the fluorescence intensity of 605 corresponds to LEV^+ concentration of 78 µM which is close to its calculated concentration of 84 µM based on the added quantity of the drug.

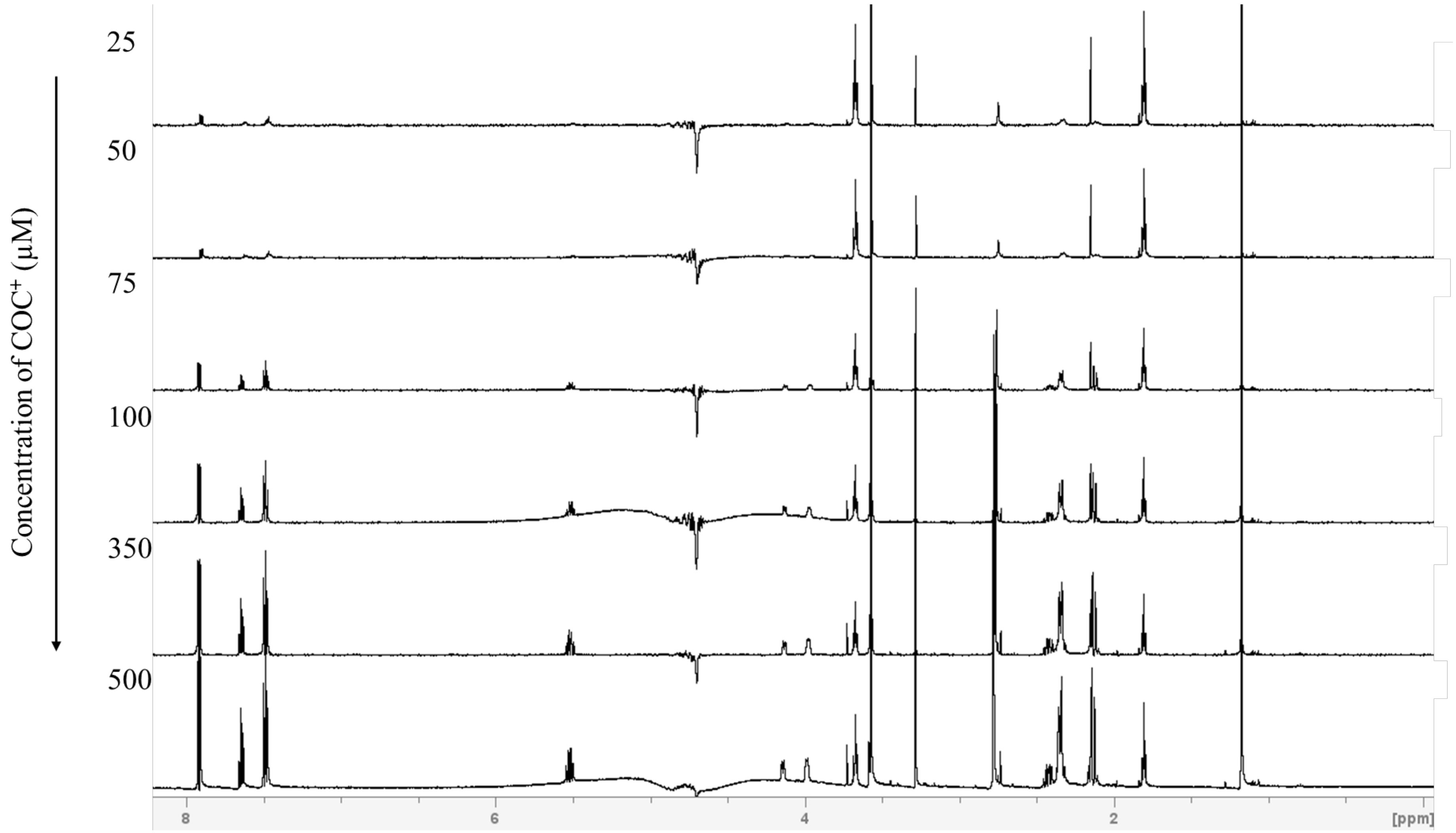

Figure S43. ^{1}H NMR spectra (600 MHz, 298 K; water suppression) of variable concentrations (25-500 μM) of cocaine COC^+ in 100 mM borate buffer at pH = 8.1 (10% D_2O). Note that resonances do not shift or become broaden suggesting the monomeric state of this drug.

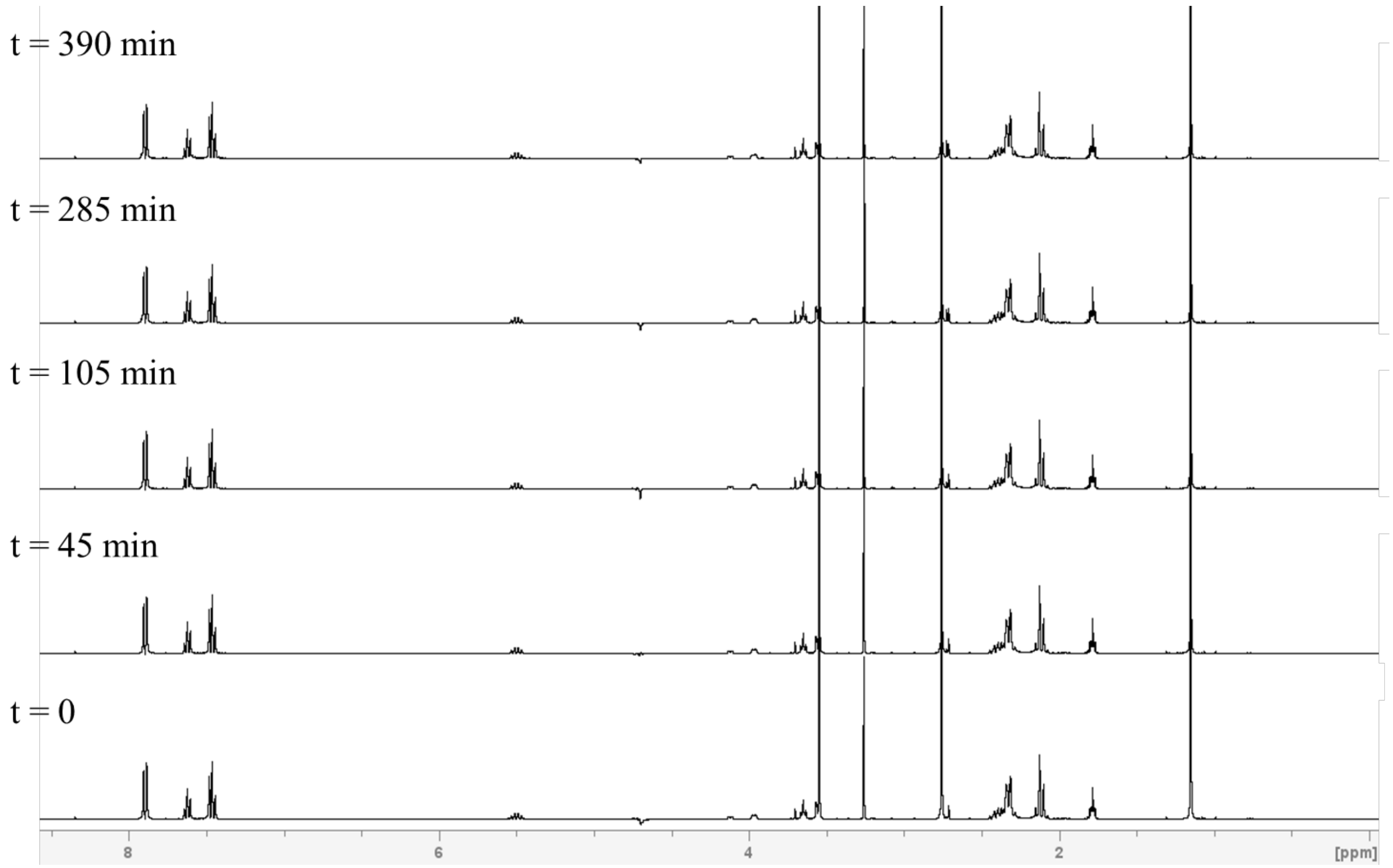

Figure S44. ^{1}H NMR spectra (600 MHz, 298 K; water suppression) of 100 µM solution of cocaine COC^{+} in 100 mM borate buffer at pH = 8.1 (10% D_2O) were recorded over 390 min. Note that there is no appearance of new signals over time therefore indicating chemical stability (i.e., ester hydrolysis) of the drug at pH = 8.1 for 390 min.

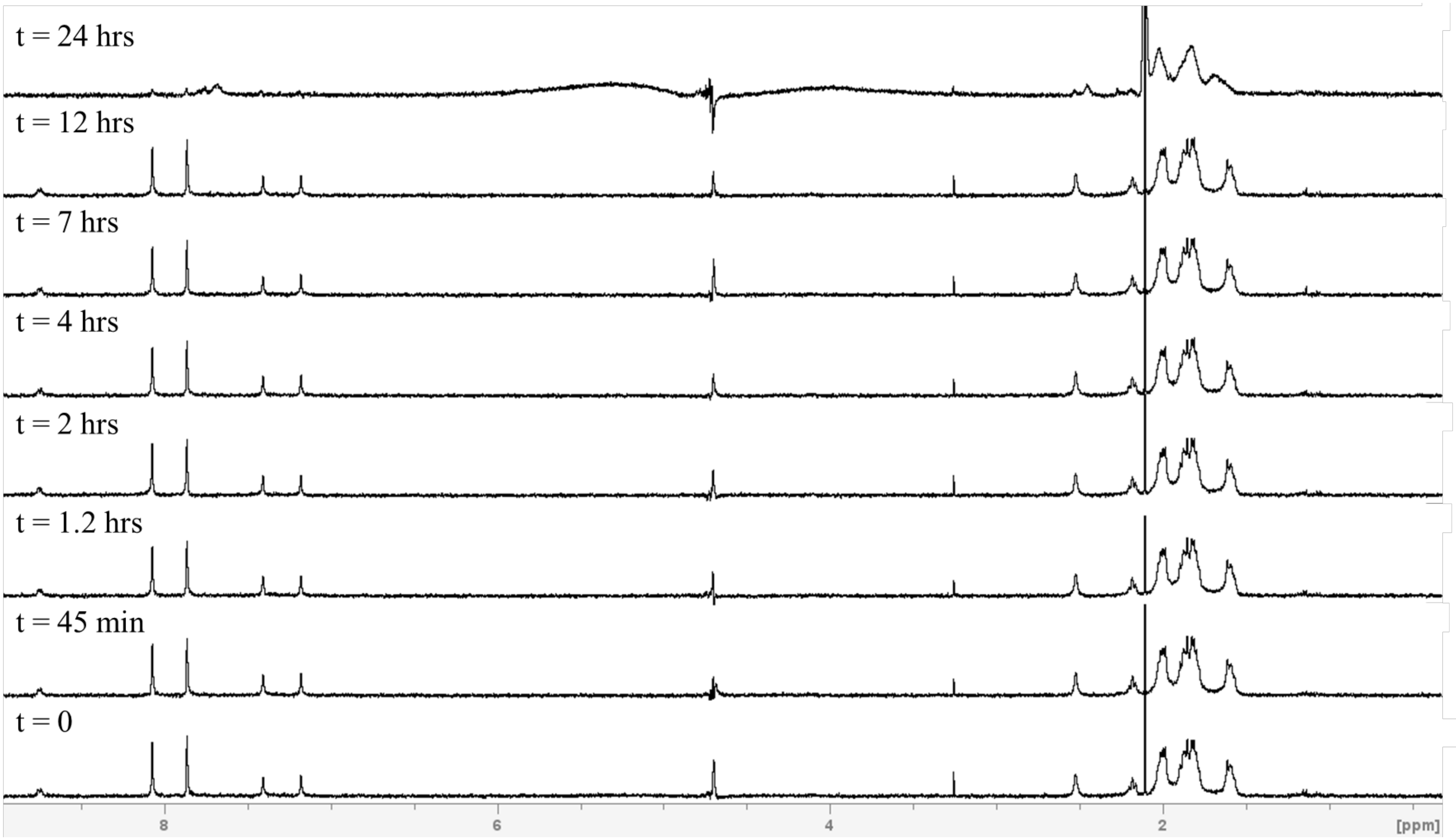

Figure S45. ^{1}H NMR spectra (600 MHz, 298 K; water suppression) of 100 µM solution of basket **6**$^{18-}$ in 100 mM borate buffer at pH = 8.1 (10% D_2O) were recorded over 24 h. Note that there is no appearance of new signals over 12 h therefore indicating chemical stability (i.e., imide hydrolysis) of the host at pH = 8.1 for 12 h.

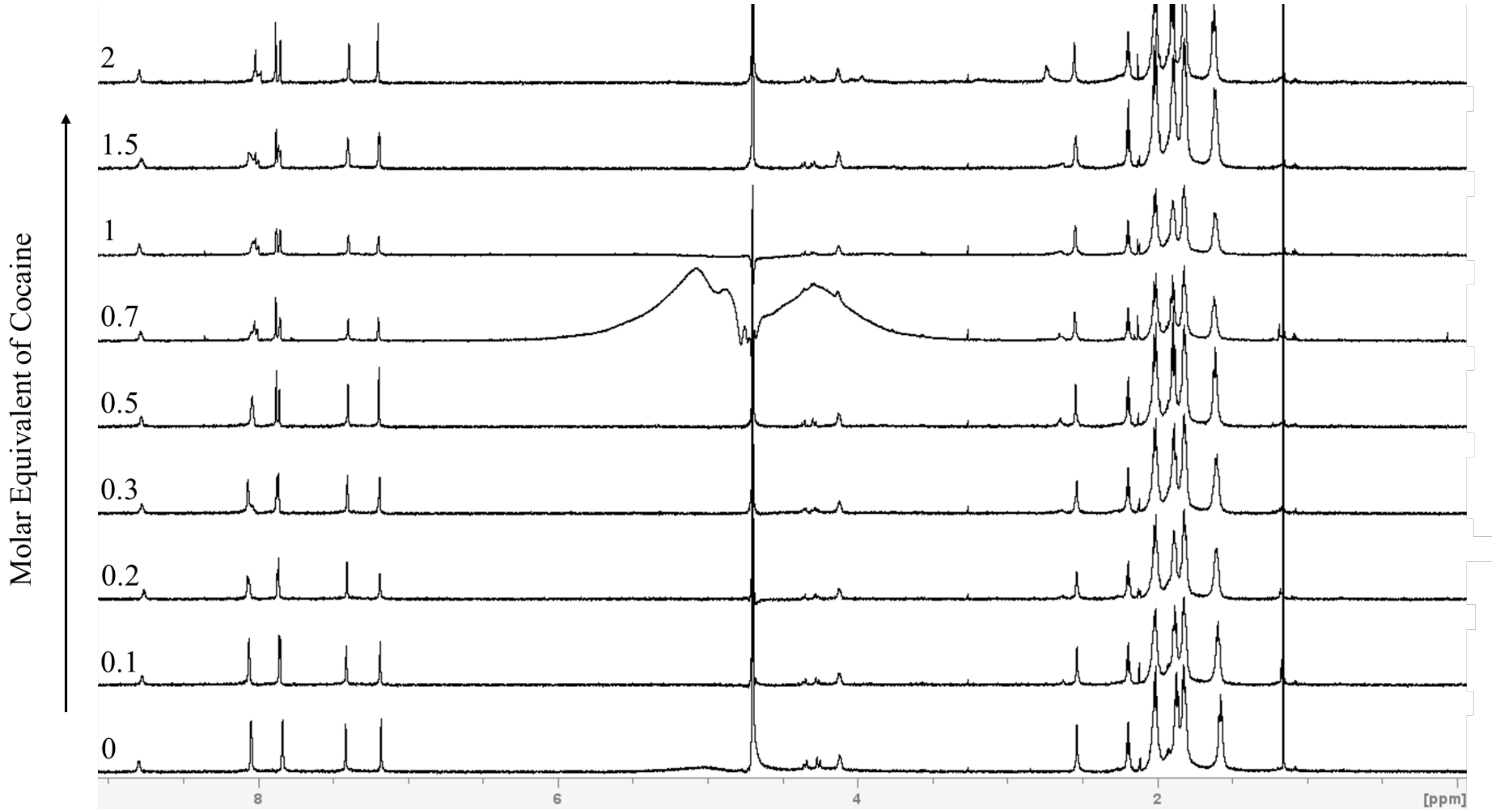

Figure S46. ^{1}H NMR spectra (850 MHz, 298 K; water suppression) of 50 µM basket **6**$^{18-}$ recorded upon an incremental addition of 250 mM solution of COC$^+$ (100 mM borate buffer at pH = 8.1). Resonances from the naphthalimide groups in **6**$^{18-}$ (H_A and H_B at circa 8 ppm) are magnetically perturbed more than others in this host.

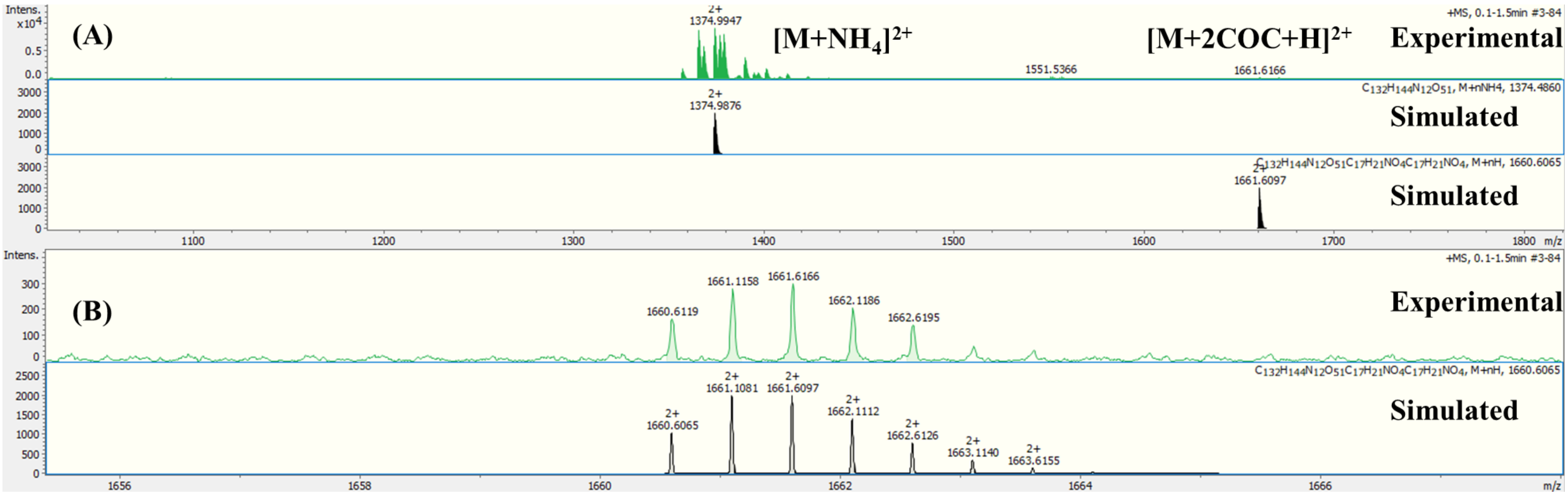

Figure S47. A: Experimental and simulated electron-spray ionization (ESI) data for 50 µM solution of molecular basket $\mathbf{6}^{18-}$ and Cocaine HCl (COC^+) (i.e., equimolar ratio) in 25 mM ammonium bicarbonate buffer at pH = 6.8. B: Experimental and simulated isotope distribution for an ion with the mass corresponding to $[COC\subset\mathbf{6}]^{2+}$.

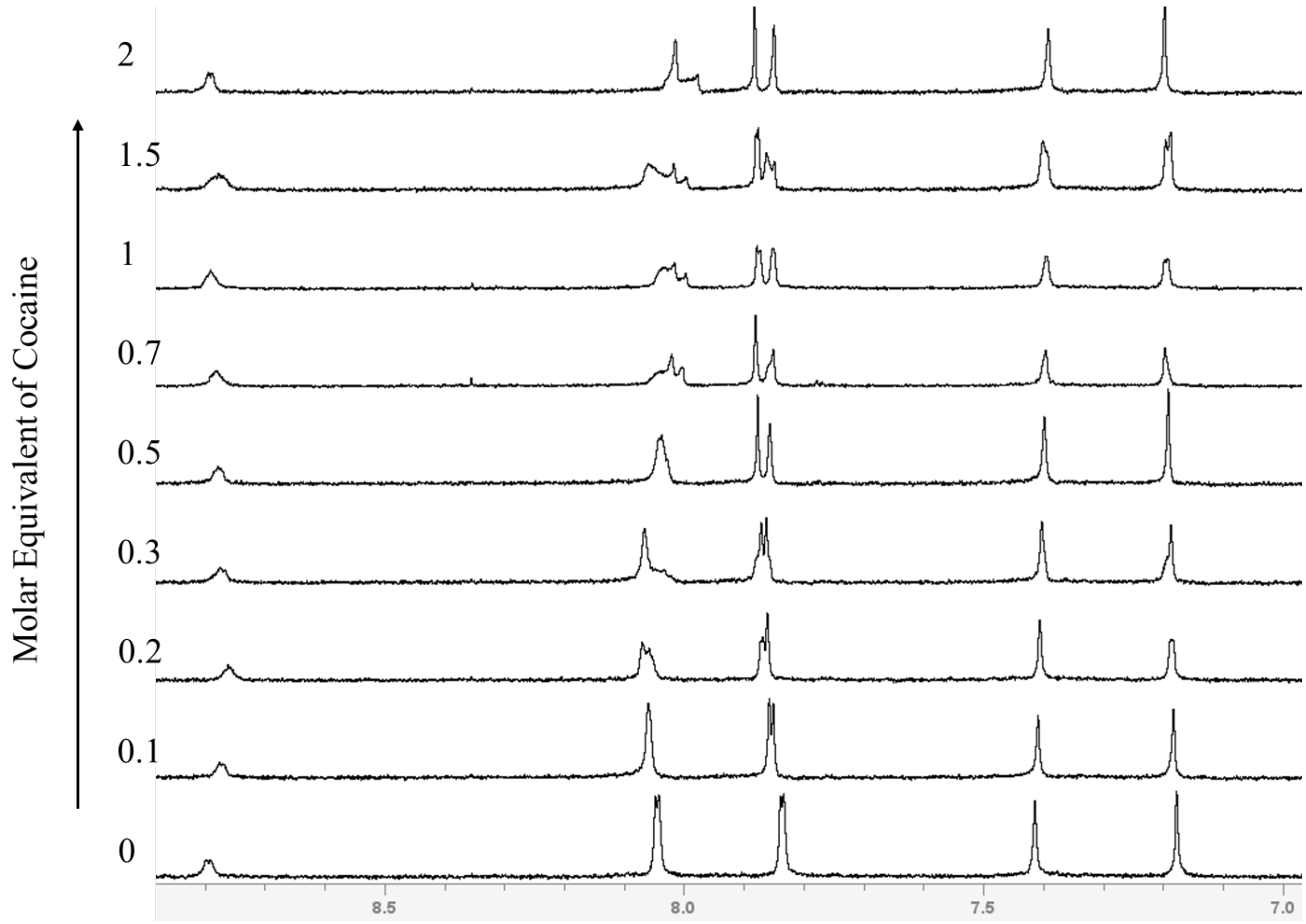

Figure S48. A segment of ^{1}H NMR spectrum (850 MHz, 298 K; water suppression) of 50 µM of basket **6**$^{18-}$ upon an incremental addition of 250 µM solution of COC^{+} in 100 mM borate buffer at pH = 8.1 (10% D_2O). It appears that there are three signals at circa 8 ppm, so we hypothesize that one could be from COC^{+} (at a lower field).

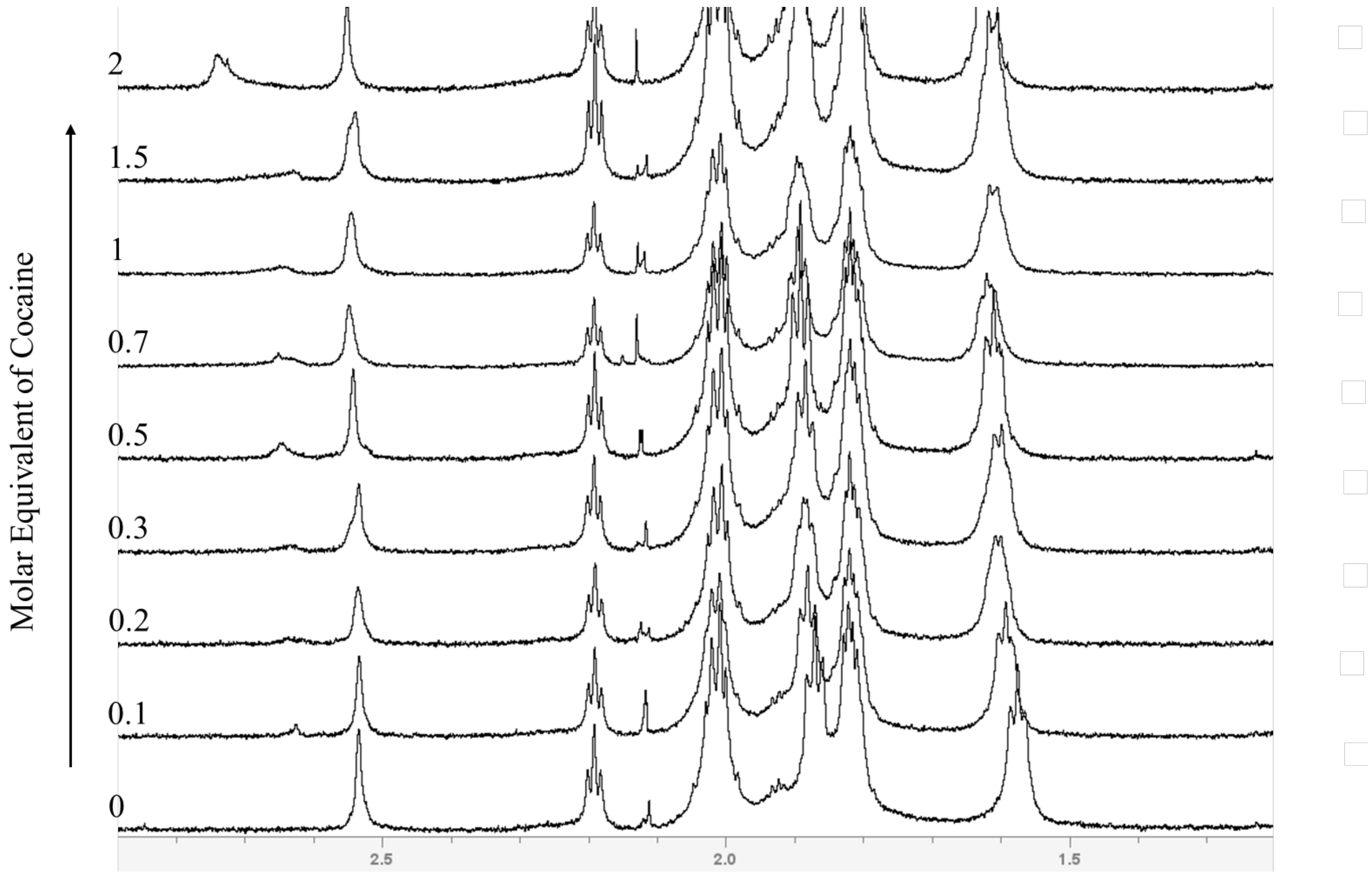

Figure S49. A segment of ^{1}H NMR spectrum (850 MHz, 298 K; water suppression) of 50 µM of basket $\mathbf{6}^{18-}$ upon an incremental addition of 250 µM solution of COC^{+} in 100 mM borate buffer at pH = 8.1 (10% D_2O). We hypothesize that a broadening signal at circa 2.6 ppm is from $^{+}N\text{-}CH_3$ group COC^{+} ($^{+}N\text{-}CH_3$ resonance from free COC^{+} is at 2.75 ppm; Figure S43).

Computational Studies

For computing docking positions of LEV$^+$ inside baskets $\mathbf{1}^{3-}$ to $\mathbf{6}^{18-}$, ([LEV$\subset$**1-6**]$^{n-}$, we completed Monte Carlo conformation searches using Maestro software (Schrodinger) and OPLS4 force field. For all six calculations, we used (a) implicit water solvent with (b) systematic torsional sampling method including intermediate torsional sampling. The maximum number of steps was set to 20000, with 200 steps per rotatable bond. The energy window for saving structures was 5 kcal/mol. The starting docking pose was, in each case, benzene group from LEV$^+$ inserted into basket's cavity (as the experimental results suggested). Figures S50-55 show most stable poses within 1 kcal/mol of steric energy.

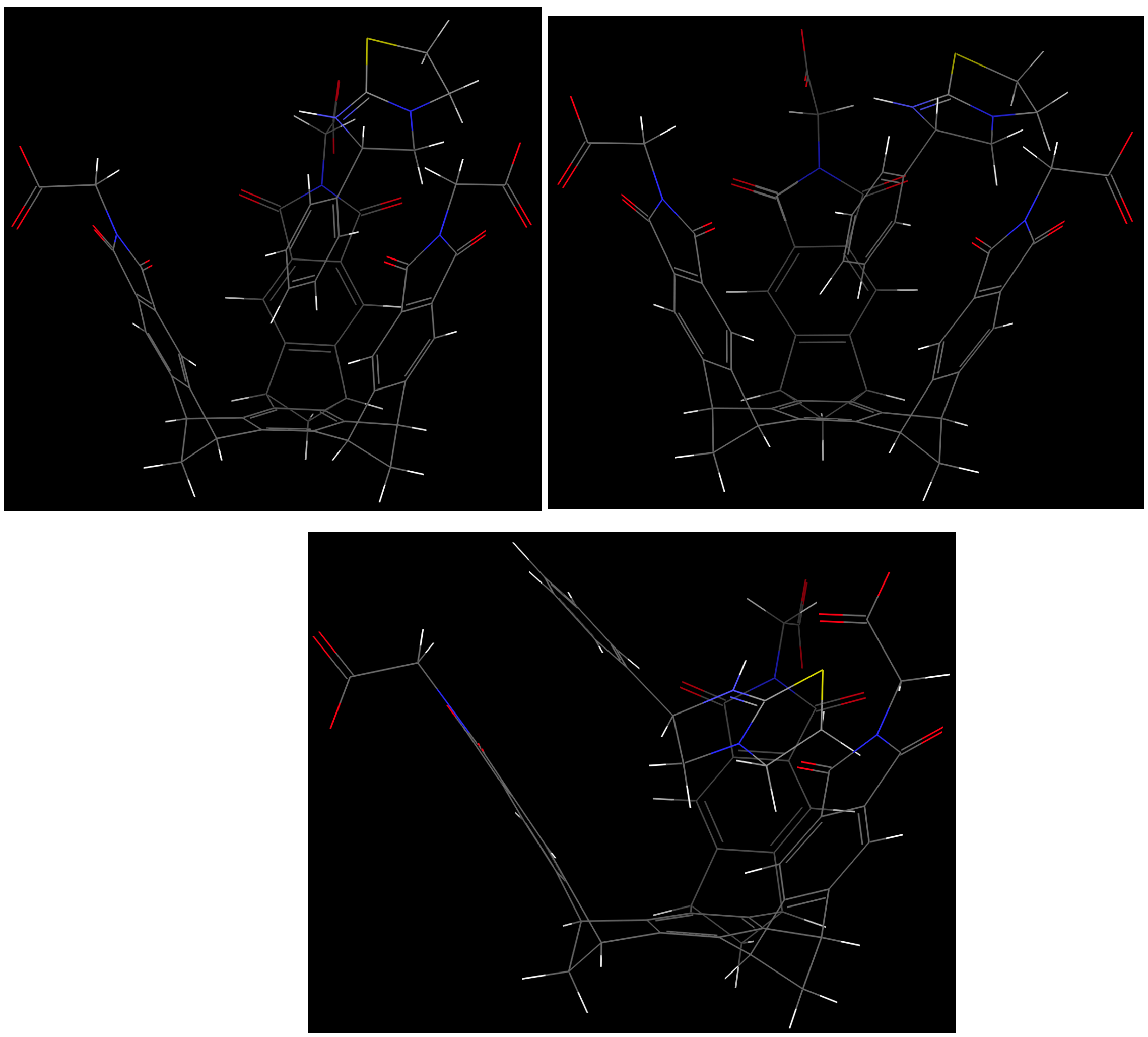

Figure S50. Three representative poses from computed [LEV$\subset$**1**]$^{2-}$ (OPLS 4) that are within 1 kcal/mol of the lowest steric energies are shown.

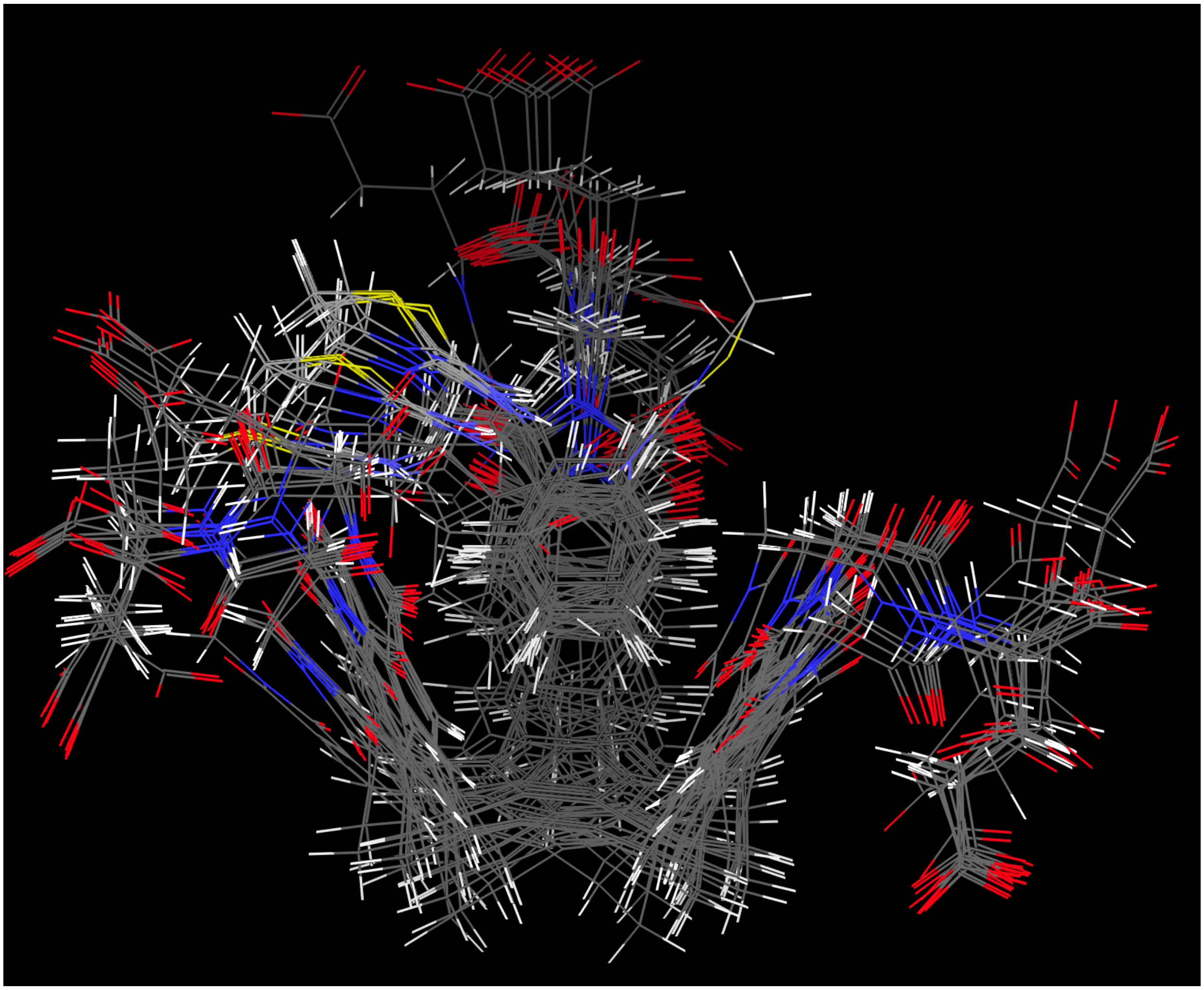

Figure S51. Sixteen most stable poses from computed [LEV$\subset$**2**]$^{5-}$ (OPLS 4) within 1 kcal/mol of steric energies are shown.

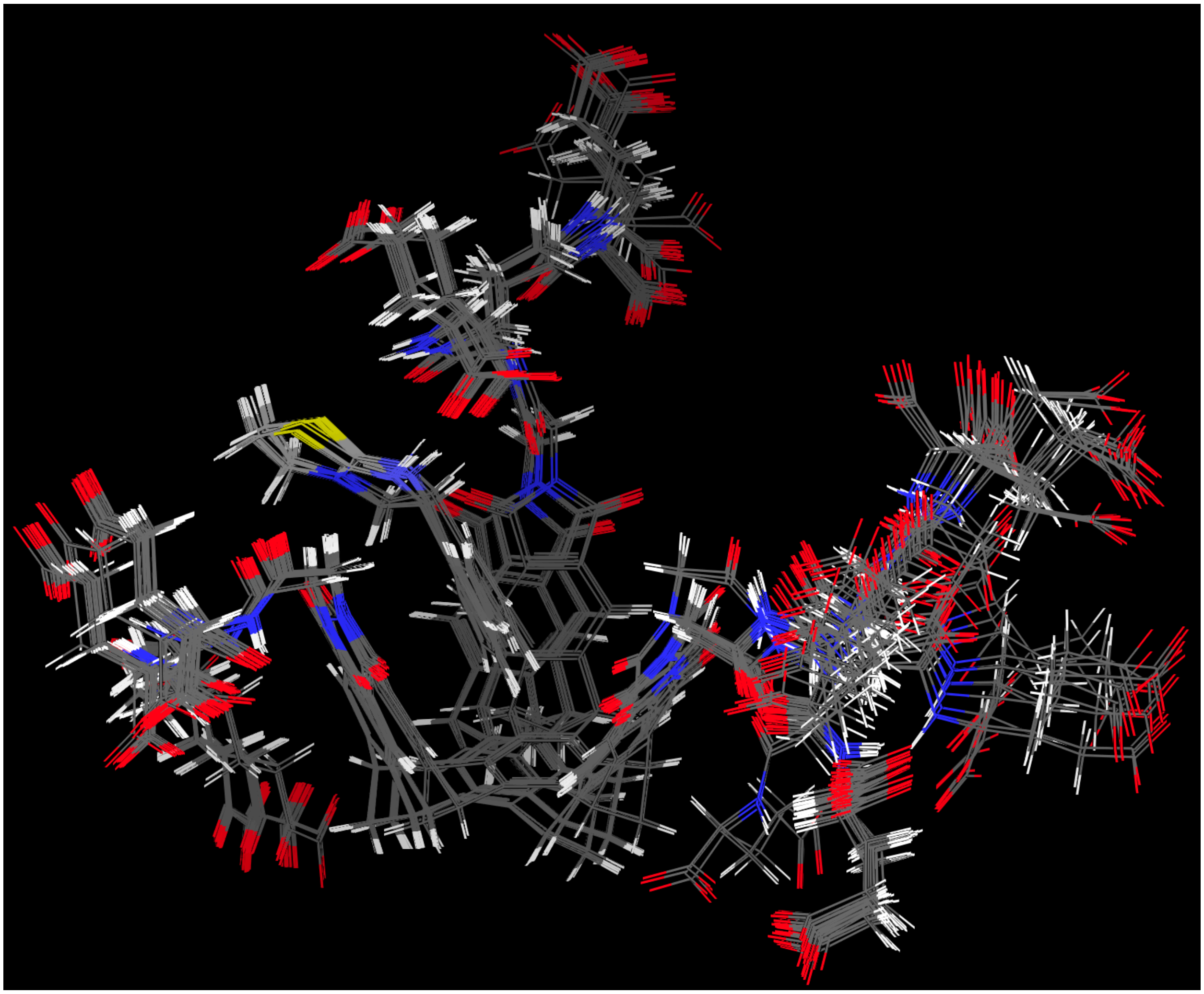

Figure S52. Thirty-seven most stable poses from computed [LEV⊂**3**]$^{11-}$ (OPLS 4) within 1 kcal/mol of steric energies are shown.

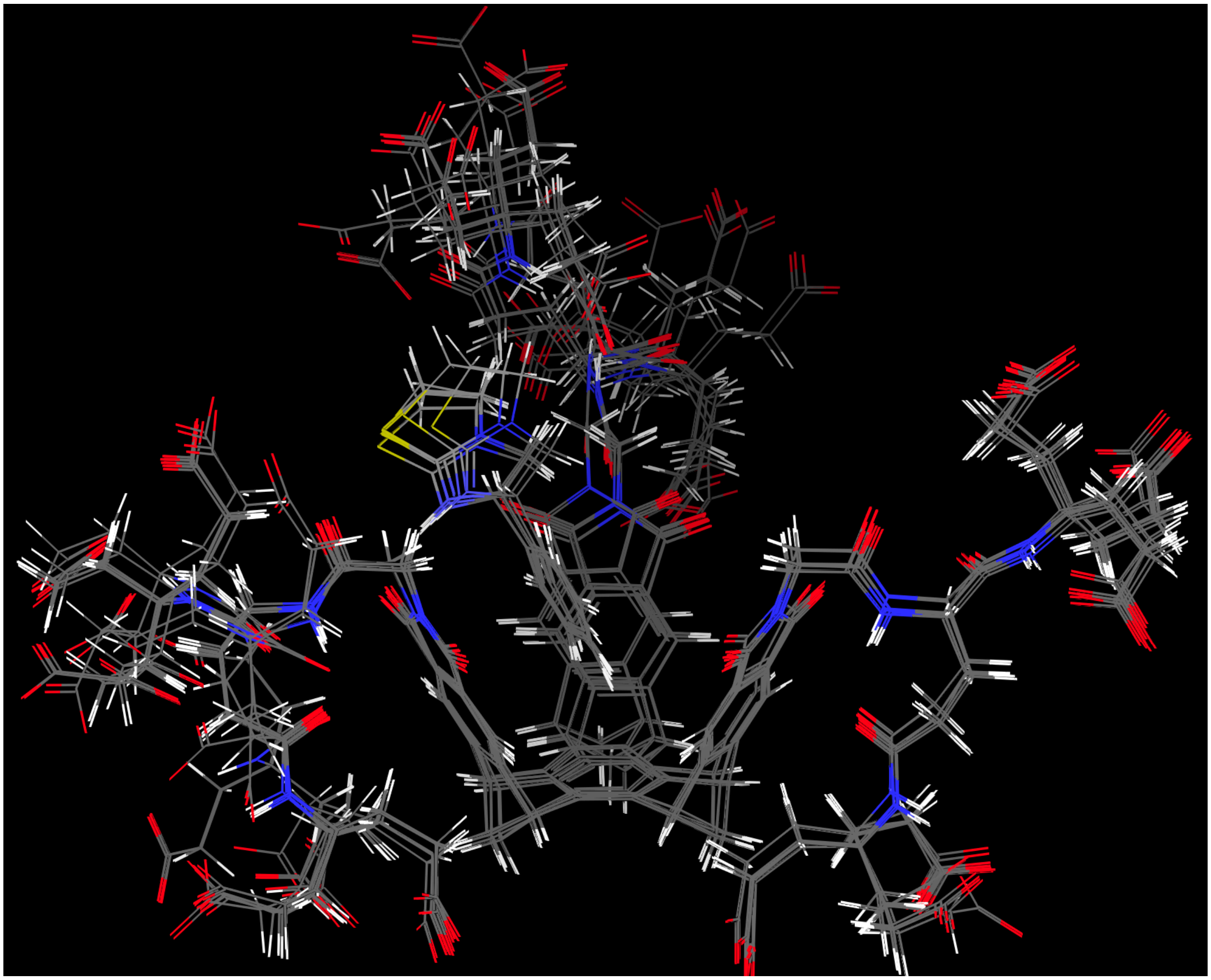

Figure S53. Eight most stable poses from computed [LEV⊂**4**]$^{17-}$ (OPLS 4) within 1 kcal/mol of steric energies are shown.

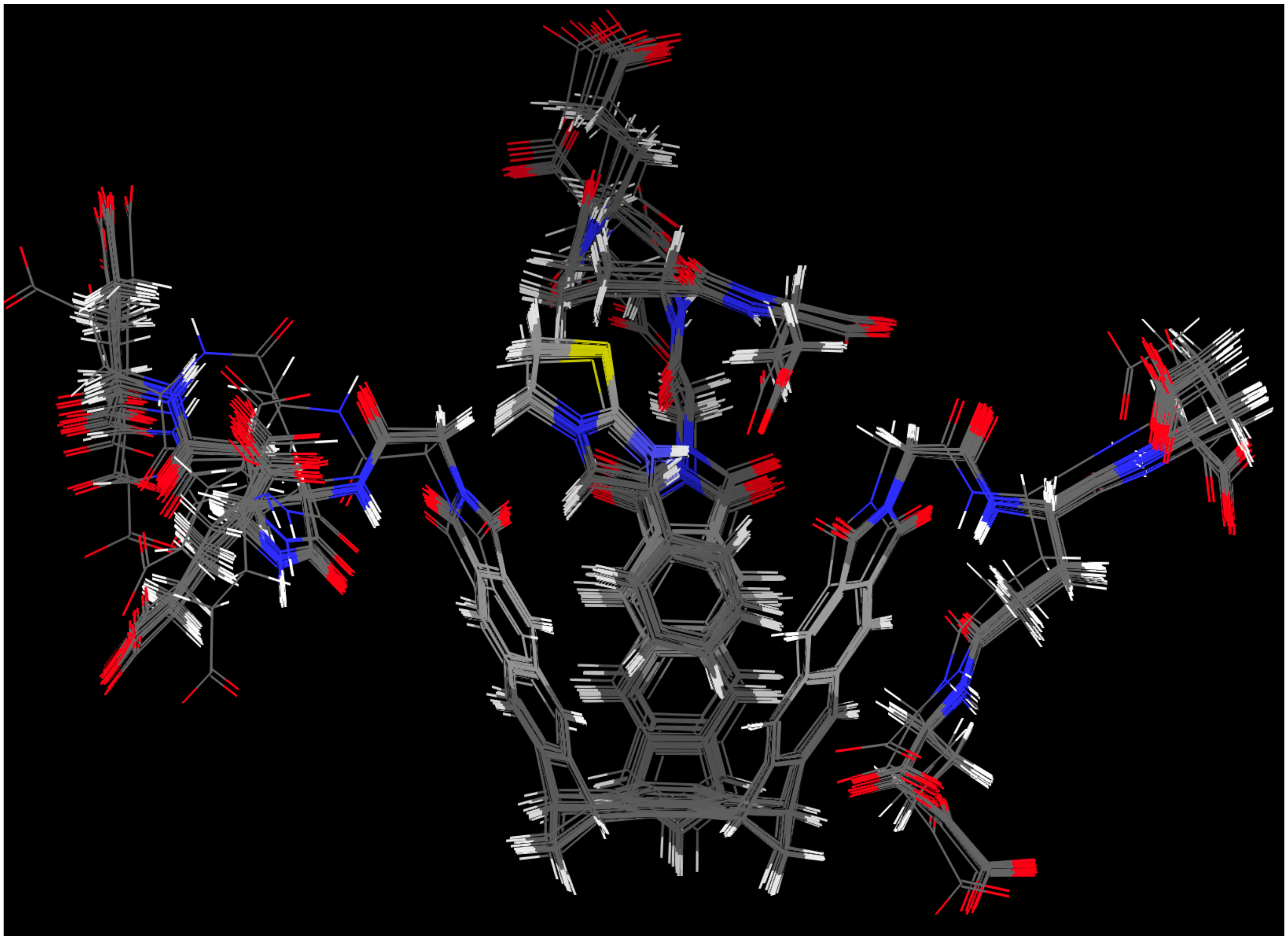

Figure S54. Eighteen most stable poses from computed [LEV⊂**5**]$^{11-}$ (OPLS 4) within 1 kcal/mol of steric energies are shown.

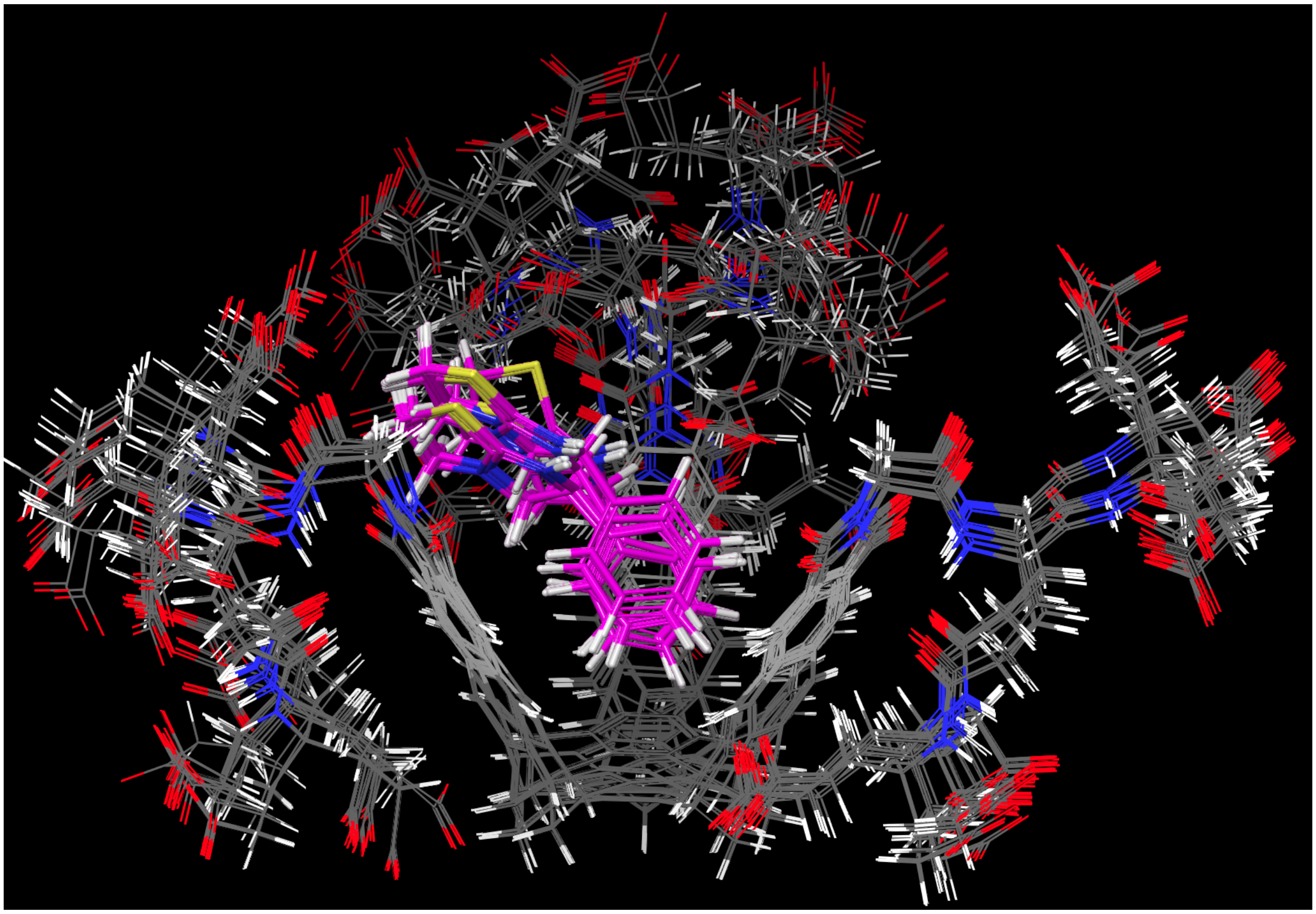

Figure S55. Twenty-four most stable poses from computed [LEV⊂**6**]$^{17-}$ (OPLS 4) within 1 kcal/mol of steric energies are shown.

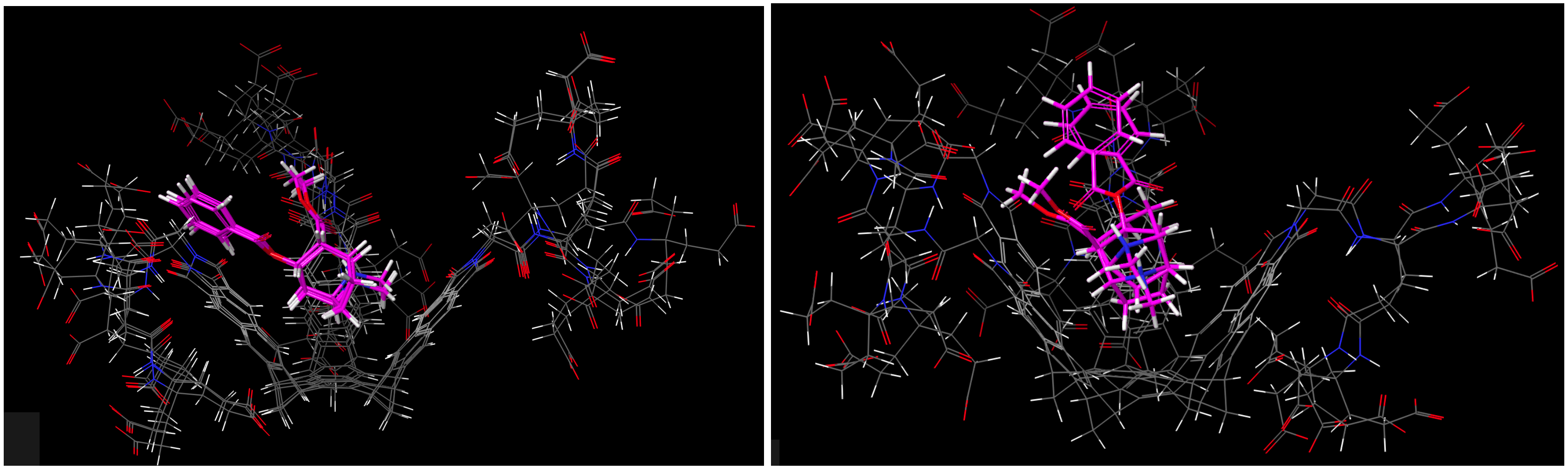

Figure S56. Six most stable binding poses from computed [COC⊂**6**]$^{17-}$ (OPLS 4, Monte Carlo) within 1 kcal/mol of steric energies are shown. For this computation (OPLS 4, water), we used torsional method of sampling with 10000 steps and 200 steps per rotatable bond. The starting geometry was the one in which benzene ring from cocaine occupies the basket's cavity.
